# *Ustilaginoidea virens*‐secreted effector Uv1809 suppresses rice immunity by enhancing OsSRT2‐mediated histone deacetylation

**DOI:** 10.1111/pbi.14174

**Published:** 2023-09-16

**Authors:** Xiaoyang Chen, Chen Liu, Hailin Wang, Qi Liu, Yaping Yue, Yuhang Duan, Zhaoyun Wang, Lu Zheng, Xiaolin Chen, Yaohui Wang, Junbin Huang, Qiutao Xu, Yuemin Pan

**Affiliations:** ^1^ Anhui Province Key Laboratory of Crop Integrated Pest Management Anhui Agricultural University Hefei China; ^2^ National Key Laboratory of Crop Genetic Improvement Huazhong Agricultural University Wuhan China; ^3^ The Key Lab of Plant Pathology of Hubei Province Huazhong Agricultural University Wuhan China; ^4^ State Key Laboratory of Agricultural Microbiology Huazhong Agricultural University Wuhan China; ^5^ Center for Excellence in Molecular Plant Sciences Chinese Academy of Sciences Shanghai China

**Keywords:** histone acetylation, immunity, OsSRT2, rice false smut, Uv1809

## Abstract

Rice false smut caused by *Ustilaginoidea virens* is a devastating rice (*Oryza sativa)* disease worldwide. However, the molecular mechanisms underlying *U*. *virens*–rice interactions are largely unknown. In this study, we identified a secreted protein, Uv1809, as a key virulence factor. Heterologous expression of *Uv1809* in rice enhanced susceptibility to rice false smut and bacterial blight. Host‐induced gene silencing of *Uv1809* in rice enhanced resistance to *U*. *virens*, suggesting that Uv1809 inhibits rice immunity and promotes infection by *U*. *virens*. Uv1809 suppresses rice immunity by targeting and enhancing rice histone deacetylase OsSRT2‐mediated histone deacetylation, thereby reducing H4K5ac and H4K8ac levels and interfering with the transcriptional activation of defence genes. CRISPR‐Cas9 edited *ossrt2* mutants showed no adverse effects in terms of growth and yield but displayed broad‐spectrum resistance to rice pathogens, revealing a potentially valuable genetic resource for breeding disease resistance. Our study provides insight into defence mechanisms against plant pathogens that inactivate plant immunity at the epigenetic level.

## Graphical abstract

The effector Uv1809 enhancing histone deacetylase OsSRT2‐modulated deacetylation, thereby reducing histone H4K5/8 acetylation levels in rice plants and interfering with defense gene activation.

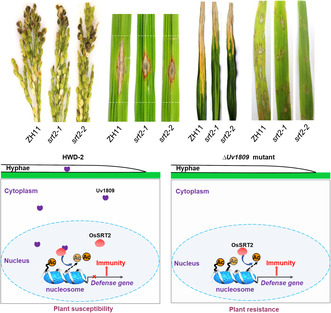

## Introduction

Plants are constantly under attack from phytopathogens in the environment. In response to these pathogens, plants have evolved a unique innate immune system (Dou and Zhou, 2012). The plant immune system consists of two layers: PTI (PAMP‐triggered immunity) triggered by PAMPs (pathogen‐associated molecular patterns) and ETI (effector‐triggered immunity) triggered by effector proteins (Dangl *et al*., 2013; Wang *et al*., 2020a). PTI is an early defence response in plants that can effectively prevent infection from a variety of phytopathogens (Dangl *et al*., 2013). PTI is activated by pattern recognition receptors (PRRs) that specifically recognize PAMPs (Couto and Zipfel, 2016). However, phytopathogens secrete virulence effectors into host cells that can block the recognition of PAMPs by PRRs, interfere with host PTI, and inhibit basal defence to successfully infect the host via effector‐triggered susceptibility (Dodds and Rathjen, 2010; Jiang *et al*., 2020; Jones and Dangl, 2006). Several host protein kinases, transcription factors, enzymes, and protein complexes involved in plant basal immunity have been identified as targets of fungal effectors, which interfere with these immune‐related target proteins and complexes using different strategies to suppress plant immunity and promote infection (Darino *et al*., 2021; Du *et al*., 2021; Fukada *et al*., 2021; Li *et al*., 2018; Tariqjaveed *et al*., 2021; Yang *et al*., 2021).

Epigenetic modifications such as histone methylation and acetylation affect the chromatin state and transcriptional regulation and are common in most organisms (Alvarez *et al*., 2010; Berger, 2007). Histone acetylation (Kac), one of the best‐studied post‐translational modifications (PTMs), plays an important role in regulating protein function and gene expression (Xu *et al*., 2021). Histone deacetylases (HDACs) and acetyltransferases (HATs) maintain the homeostasis of acetyl groups on lysine residues of histone proteins. HATs and HDACs are also involved in regulating plant immunity. In *Arabidopsis thaliana* (Arabidopsis), the histone acetyltransferase ELP3 (ELONGATOR COMPLEX SUBUNIT 3) positively regulates plant immunity by promoting the expression of defence‐related genes (DeFraia *et al*., 2013). The histone deacetylase HDA19 activates the jasmonic acid/ethylene (JA/ET) signalling pathway by promoting the expression of *ERF1* (*ETHYLENE RESPONSE FACTOR 1*) to enhance resistance against black spot disease. In addition, HDA19 interacts with the transcription factors WRKY38 and WRKY62 and inhibits their transcriptional activity, thereby positively regulating salicylic acid (SA) synthesis and enhancing disease resistance (Choi *et al*., 2012; Kim *et al*., 2008). HDA19 regulates PR (pathogen resistance) gene expression by mediating histone acetylation (Zhou *et al*., 2005). Similarly, HDA6 mediates histone acetylation to repress the expression of PR genes and inhibit SA biosynthesis by directly controlling the expression of *CBP60g* (*CAM‐BINDING PROTEIN 60‐LIKE* *G* ) and *SARD1* (*SAR DEFICIENT 1*) during pathogen infection (Wang *et al*., 2017; Wu *et al*., 2021a). The NAD^+^‐dependent Sirtuin family histone deacetylase SRT2 (SIRTUIN 2) represses the expression of the SA biosynthesis genes *SID2* (*SALICYLIC ACID INDUCTION DEFICIENT 2*), *PAD4* (*PHYTOALEXIN DEFICIENT 4*) and *EDS5* (*ENHANCED DISEASE SUSCEPTIBILITY 5*), and SRT2 negatively regulates plant resistance to *Pseudomonas syringae* (Wang *et al*., 2010). In the SA signalling pathway, HAC1/5 (HISTONE ACETYLTRANSFERASE OF THE CBP FAMILY 1/5) is recruited by a TGACG‐binding (TGA) transcription factor to form a coactivator complex with NPR1 (NONEXPRESSER OF PR GENES 1) that activates PR gene transcription via histone acetylation‐mediated epigenetic reprogramming (Jin *et al*., 2018). In rice (*Oryza sativa*), HDT701 (Histone Deacetylase 701) negatively regulates resistance to rice blast by modulating histone H4 acetylation to affect the expression of disease‐resistance genes (Ding *et al*., 2012). The histone deacetylases HDA705 and HDA701 negatively regulate resistance to rice blast, rice false smut (RFS), and bacterial blight (Chen *et al*., 2021, 2022a). These previous studies indicate that HATs and HDACs modulate histone acetylation to affect plant immunity.

RFS, one of the most devastating diseases affecting rice production worldwide, is caused by *Ustilaginoidea virens* infection (Fan *et al*., 2020; Sun *et al*., 2020). RFS not only reduces yield but also threatens animal and human health by producing cyclic peptide mycotoxins (Hu *et al*., 2019). Over 421 effectors are predicted to exist in *U*. *virens* based on genomic analysis (Zhang *et al*., 2014, 2021). Recent studies have demonstrated that a number of these effectors play critical roles in infection, including SCRE1, SCRE4, SCRE6, SGP1, UvCBP1, UvPr1a, and UvSec117 (Chen *et al*., 2022a,b; Li *et al*., 2021a; Qiu *et al*., 2022; Song *et al*., 2021; Yang *et al*., 2022; Zhang et al., 2020; Zheng *et al*., 2022). For example, the effector SCRE6 acts as a tyrosine phosphatase that dephosphorylates the negative defence regulator OsMPK6 (MAP Kinase) to enhance its accumulation, thereby inhibiting rice immunity (Zheng *et al*., 2022). However, knowledge about the molecular mechanisms underlying the suppression of rice immunity by individual *U*. *virens* effectors is limited.

In this study, we identified Uv1809 as a key effector targeting the rice histone deacetylase OsSRT2, which is a negative regulator of innate immunity against rice pathogens. Uv1809 enhanced OsSRT2‐mediated histone deacetylation, thereby reducing H4K5ac and H4K8ac levels in the host and interfering with the activation of defence gene transcription. Our study reveals a novel counter‐defence mechanism whereby plant pathogens secrete effectors to interfere with host epigenetic modifications and thereby inactivate host defence responses.

## Results

### Uv1809 is required for full virulence of *U*. *virens*

**Figure 1:**
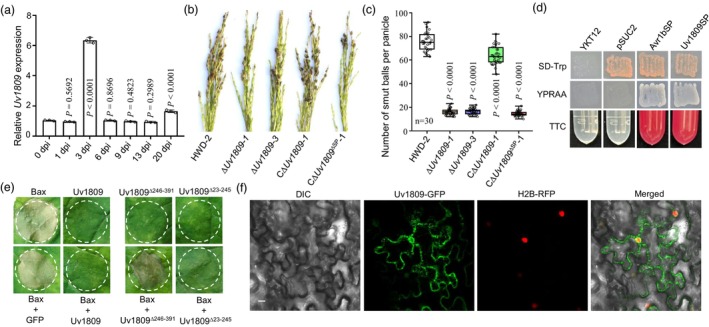
Uv1809 is a key virulence effector. (a) RT‐qPCR analysis of the expression of *Uv1809* in different infection stages on rice spikelets (1–20 dpi). Data are presented as mean ± SD (*n* = 3). The *P* values were determined by unpaired *t*‐tests compared with 0 dpi. (b) Virulence assays of the wild‐type HWD‐2, Δ*Uv1809* mutants, CΔ*Uv1809‐1* and C*∆Uv1809* ^*∆SP*^ *‐1* strains on rice cultivar Wanxian98 at 21 dpi. (c) Number of rice smut balls per panicle. Data were collected from three independent experiments for each treatment. The *P* values were determined by unpaired *t*‐tests compared with the wide‐type strain HWD‐2. (d) The yeast YTK12 and its transformants expressing the empty vector pSUC2 (negative control) or pSUC2‐*Uv1809SP* and pSUC2‐*Avr1SP* (positive control) were tested for growth on SD‐Trp or YPRAA medium and invertase activity in TTC medium. (e) Transient expression of Uv1809 suppressed programmed cell death of *Nicotiana benthamiana* leaves triggered by Bax. Representative leaves were photographed at 4 dpi. (f) Subcellular localization of Uv1809‐GFP in *N*. *benthamiana* leaves. *N*. *benthamiana* histone H2B protein was used as a nuclear localization marker protein. DIC, differential interference contrast; GFP, green fluorescent protein. Scale bar = 20 μm.

Based on transcriptome data from rice spikelets infected by *U*. *virens* (Tang *et al*., 2021), we found that *Uv8b_ 1809* (renamed *Uv1809* in this work) was significantly upregulated in the early stage of infection. RT‐qPCR analysis suggested that *Uv1809* expression is significantly upregulated at 3 days post inoculation (dpi) (Figure 1a), suggesting that *Uv1809* plays an important role in the interaction between *U*. *virens* and rice. *Uv1809* encodes a 391‐aa protein, which contains a signal peptide (SP) and is functionally annotated as a hypothetical protein (Figure S1a). Phylogenetic tree analysis showed that Uv1809 homologues are widely distributed in fungi, but no homologous proteins were identified in animals or plants (Figure S1b). Notably, the C end of *Uv1809* contains numerous repeat sequences (Figure S1c). Therefore, we generated *Uv1809* knockout mutants (∆*Uv1809‐1* and ∆*Uv1809‐3*) and the complementation strain C∆*Uv1809‐1* using *Agrobacterium tumefaciens*‐mediated transformation (ATMT). Transformants were identified by PCR analysis and Southern blot (Figure S2a–d). Pathogenicity in the susceptible rice cv. ‘Wanxian‐98’ was tested, and the virulence of *∆Uv1809* mutants was significantly reduced compared to the wild‐type strain HWD‐2 and the complementation strain (Figure 1b, c). Compared with the wild‐type strain HWD‐2 and the complementation strain, ∆*Uv1809* mutants displayed no significant differences in mycelial growth rate and conidiation (Figure S3a–c). These results show that *Uv1809* is required for full virulence of *U*. *virens*.

### The signal peptide of Uv1809 is essential for its secretion and function

To investigate the functionality of the SP of Uv1809, we used a yeast secretion system to verify the secretion activity of the protein (Jacobs *et al*., 1997). The sequence encoding the 22‐aa SP was ligated with the pSUC2 vector and then transformed into yeast strain YKT12. *Uv1809SP* and positive control *Avr1bSP* transformants could grow on YPRAA, but the negative control *pSUC2* transformants and the untransformed YTK12 strain could not grow on YPRAA medium. The *Uv1809SP* and *Avr1bSP* transformants also secreted invertase and turned the TTC solution red, while the negative control *pSUC2* transformants and the untransformed YTK12 strain could not change the colour of the TTC solution (Figure 1d). These results indicate that the SP of Uv1809 is a functional secretion signal, and Uv1809 is a secreted protein in *U*. *virens*.

To assess whether the SP of Uv1809 is related to the virulence of *U*. *virens*, we then generated a *Uv1809* construct lacking the SP (*Uv1809* ^ΔSP^) and introduced it into the ∆*Uv1809‐1* mutant. The resulting C∆*Uv1809* ^ΔSP^ *‐1* transformant strain showed a similar virulence to that of the ∆*Uv1809‐1* mutant and exhibited significantly reduced virulence compared to the wild‐type strain HWD‐2 and the complementation strain (Figure 1b, c). These results indicate that the SP of Uv1809 plays a key role in the virulence of *U*. *virens*.

### Uv1809 suppresses Bax‐induced cell death in *Nicotiana benthamiana*

To characterize the role of Uv1809 in host immune responses, a pVX‐*Uv1809* construct was generated and introduced into *Agrobacterium* strain EHA105. In *N*. *benthamiana* leaves infiltrated with *Agrobacterium* expressing *Uv1809* alone, no cell death was observed (Figure 1e). Under the same conditions, cell death was observed on *N*. *benthamiana* leaves infiltrated with *Agrobacterium* expressing the mouse proapoptotic protein Bax. However, the cell death induced by Bax was suppressed when Uv1809 and Bax proteins were co‐expressed (Figure 1e). As a negative control, expression of the empty vector pVX (GFP) and Bax did not abolish Bax‐dependent cell death (Figure 1e). To delineate the region within Uv1809 that mediates the inhibition of Bax‐induced cell death, we generated constructs expressing either the N‐terminal (Uv1809^Δ246–391^) or C‐terminal (Uv1809^Δ23–245^) portion of Uv1809 and co‐infiltrated them with *Bax* in *N*. *benthamiana*. Only the construct encoding the C‐terminal portion of Uv1809 (Uv1809^Δ23–245^) showed an ability to suppress Bax‐induced cell death comparable to full‐length Uv1809 (Figure 1e). These results suggest that Uv1809 suppresses the cell death induced by Bax, possibly by interfering with the plant immune response.

### Uv1809 is an intracellular effector

To further determine the subcellular localization of Uv1809 in *N*. *benthamiana* leaves, the pCNG‐*Uv1809* construct was generated and introduced into *Agrobacterium* strain EHA105. In *N*. *benthamiana* leaves infiltrated with *Agrobacterium* expressing the Uv1809‐GFP fusion protein, fluorescence microscopy detected GFP fluorescence in the nucleus and the cytoplasm, and the fluorescence signal will gather in some positions (Figure 1f, Figure S4), which is different from the localization of Uv1809^ΔSP^‐GFP and empty GFP (Figure S4). After plasmolysis, the GFP fluorescence signal in the intracellular, not in apoplastic (Figure S4). These results suggests that Uv1809 is an intracellular effector.

To investigate whether Uv1809 is secreted and translocated into plant cells, Uv1809 carrying a nuclear localization signal (NLS) was ectopically expressed as a fusion with GFP in *Magnaporthe oryzae* strain P131 driven by the RP27 promoter. The engineered *M*. *oryzae* strains were inoculated onto detached barley leaves. Green fluorescence was detectable inside the invasive hyphae of GFP‐labelled *M*. *oryzae* by microscopy at 30 h after inoculation (Figure S5). The majority of barley epidermal cells infected by Uv1809‐GFP‐NLS‐transformed *M*. *oryzae* exhibited green fluorescence in the nuclei at 30 h after inoculation (Figure S5). When barley leaf sheath was inoculated with the engineered *M*. *oryzae* strain ectopically expressing Uv1809‐GFP, green fluorescence was clearly observed to accumulate in the BIC at 30 h post inoculation (hpi). In contrast, green fluorescence was only discernible in invasive hyphae after barley sheaths were infected by GFP‐expressing *M*. *oryzae* (Figure S5). These results indicate that Uv1809 can be secreted into plant cells.

Since Uv1809‐GFP was present in the nucleus, we looked for nuclear localization sequences (NLSs) in Uv1809 using the tool NLStradamus (https://www.novopro.cn/tools/nls‐signal‐prediction.html) (Nguyen *et al*., 2009). Uv1809 contained one potential NLS at aa 336–367 (KDSKDNKDSKANKDSKDNKDSRAVNKPEGKAAR). Accordingly, we expressed the Uv1809^336–367^‐GFP fusion protein in *N*. *benthamiana* leaves, the subcellular localization result showed the putative NLS was functional which succeeded in localizing GFP to the nucleus (Figure S4). We also tested the subcellular localization of Uv1809^Δ246–391^ and Uv1809^Δ23–245^ when transgenically expressed in *N*. *benthamiana* leaves. The Uv1809^Δ246–391^‐GFP variant showed an almost complete loss of nuclear signal, while the Uv1809^Δ23–245^‐GFP variant displayed strong GFP fluorescence in the nucleus (Figure S4). These results indicate that aa 246–391 of Uv1809 plays an important role in regulating the nuclear localization of Uv1809.

### Heterologous expression of *Uv1809* increases susceptibility to rice pathogens

**Figure 2:**
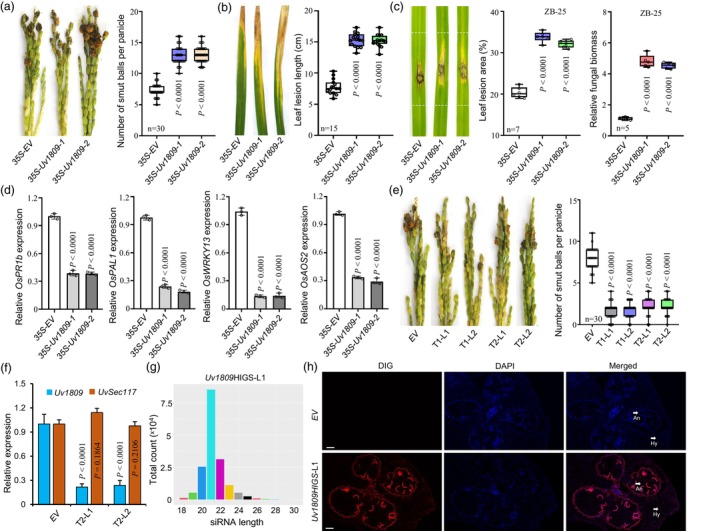
Heterologous overexpression of *Uv1809* increases susceptibility to rice pathogens and HIGS of *Uv1809* enhances rice resistance against *Ustilaginoidea virens*. (a) Left: Resistance assays of *35S‐Uv1809* and *35S‐EV* transgenic rice lines to *U*. *virens* strain HWD‐2 infection at 25 dpi. Right: Mean number of rice smut balls measured in resistance assays. (b) Left: Disease symptoms at 14 dpi of *35S‐Uv1809* and *35S‐EV* transgenic rice lines after inoculation with *Xoo* PXO99. Right: Mean lesion lengths at 14 dpi on the leaves of *35S‐Uv1809* and *35S‐EV* transgenic rice lines after inoculation with *Xoo* PXO99. (c) Disease symptoms (Left), leaf lesion area (Middle) and the relative fungal biomass (Right) of *35S‐Uv1809* and *35S‐EV* transgenic rice lines after spot‐inoculation with *Magnaporthe oryzae* ZB‐25 at 7 dpi. Relative fungal biomass was determined using quantitative reverse transcription (RT‐qPCR) for *M*. *oryzae Pot2* and normalized to rice *OsUBQ1*. The leaf lesion area was measured using Image J software. (d) RT‐qPCR analysis of defence‐related genes at 1 dpi in *35S‐EV* and *35S‐Uv1809* transgenic rice lines inoculated with *U*. *virens*. (e) Left: Resistance assays of *Uv1809*HIGS and *EV* transgenic rice lines against *U*. *virens* strain HWD‐2 at 25 dpi. Right: Mean number of rice smut balls measured in resistance assays. (f) Relative mRNA expression of *Uv1809* of *U*. *virens* during infection in the T2 transgenic rice lines at 6 dpi. (g) Length distribution and abundance of siRNAs targeting *Uv1809*HIGS*‐*L1 in T2 transgenic rice plants. (h) Visualization of siRNAs targeting *Uv1809* in infected *Uv1809*HIGS*‐*L1 rice spikelets at 6 dpi by FISH using a specific probe. An, anther; hy, *U*. *virens* hyphae. Scale bar = 20 μm. All data are presented as mean ± SD (*n* = 3 unless otherwise indicated) and analysed by Fisher's least significant difference (LSD) test. The *P* values were determined by unpaired *t*‐tests compared with the *35‐EV* or *EV*.

To further determine how Uv1809 suppresses plant immune responses, we generated transgenic rice lines heterologously expressing *Uv1809*. These *35S‐Uv1809* transgenic rice lines grew similarly to the empty vector control transgenic rice lines (*35S‐EV*) (Figure S6). However, two independent *35S‐Uv1809* transgenic rice lines had more rice smut balls than the *35S‐EV* transgenic rice lines after inoculation with *U*. *virens* strain HWD‐2 (Figure 2a). Likewise, *35S‐Uv1809* transgenic rice lines were more susceptible to infection by *Xoo* strain PXO99, with lesion lengths in *35S‐Uv1809* transgenic rice lines being about two times longer than those in *35S‐EV* transgenic rice lines at 14 dpi (Figure 2b). We then evaluated the *35S‐Uv1809* transgenic rice lines for resistance against the rice blast fungus *M*. *oryzae* strain ZB‐25. The *35S‐Uv1809* transgenic rice lines were more susceptible to *M*. *oryzae* than the *35S‐EV* transgenic rice lines at 7 dpi (Figure 2c). We also found that the expression of defence‐related genes *OsPR1b*, *OsPAL1*, *OsWRKY13*, and *OsAOS2* was significantly downregulated in the *35S‐Uv1809* transgenic rice plants (Figure 2d), suggesting that *Uv1809* leads to the suppression of the immune response. Taken together, these results indicate that heterologous expression of *Uv1809* inhibits the host's immune response, thereby enhancing the susceptibility of rice plants to pathogens.

### Engineering RFS‐resistant rice via HIGS of *Uv1809*

We employed host‐induced gene silencing (HIGS) by generating transgenic *japonica* rice cv. *Nipponbare* plants expressing an RNA interference (RNAi) construct against the *Uv1809* transcript (*Uv1809*HIGS; Figure S7). At 25 dpi with *U*. *virens*, we scored an average of 7 smut balls per panicle on the spikelets of rice plants transformed with the empty vector (*EV*). By contrast, two independent *Uv1809*HIGS transgenic rice lines displayed strong resistance to RFS, with an average of 1 to 3 smut balls per panicle (Figure 2e). To confirm that the resistance to RFS seen in infected *Uv1809*HIGS transgenic plants is caused by *in planta* silencing of *Uv1809*, we collected infected spikelets from T_2_ transgenic rice lines (*Uv1809*HIGS and *EV* control) at 6 dpi and quantified *Uv1809* and *UvSec117* transcript levels by RT‐qPCR. Indeed, relative *Uv1809* transcript levels were much lower in the two *Uv1809*HIGS transgenic rice lines than in the *EV* control lines (Figure 2f), while the relative transcript level of the control effector encoding gene *UvSec117* was no significant change; this result shows that the *Uv1809*HIGS transgenic plants resistance to RFS is caused by *in planta* silencing of *Uv1809*.

To ascertain whether silencing of *Uv1809* in infecting *U*. *virens* is mediated by small interfering RNAs (siRNAs) generated by the *Uv1809*HIGS transgenic rice lines, we sequenced small RNAs in the *Uv1809*HIGS‐L1 transgenic rice lines. The sequencing data indicated that siRNAs mapping to *Uv1809* were highly abundant in *Uv1809*HIGS‐L1 transgenic rice lines, accounting for 0.83% of all small RNAs detected in these lines. The siRNAs matching the *Uv1809* transcript had a size distribution of between 18 and 30 nucleotides, with 21‐nucleotide siRNAs being the most abundant (Figure 2g). In fluorescence *in situ* hybridization (FISH) assays, fluorescence signal was observed both in rice flower tissue and *U*. *virens* infection hyphae in the infected *Uv1809*HIGS‐L1 transgenic rice lines at 6 dpi, whereas no fluorescence signal was detected in the *EV* control lines (Figure 2h). This result supports the notion that the *Uv1809*‐RNAi vector produces effective siRNAs against *Uv1809* in the *Uv1809*HIGS*‐L1* transgenic rice lines that are then translocated to fungal cells during infection to reduce *Uv1809* transcript levels in invading *U*. *virens* hyphae. Together, these results demonstrate that silencing of *Uv1809* in transgenic rice plants promotes resistance to *U*. *virens*, indicating that Uv1809 is a key virulence effector during infection.

### Uv1809 interacts with the rice histone deacetylase OsSRT2

**Figure 3:**
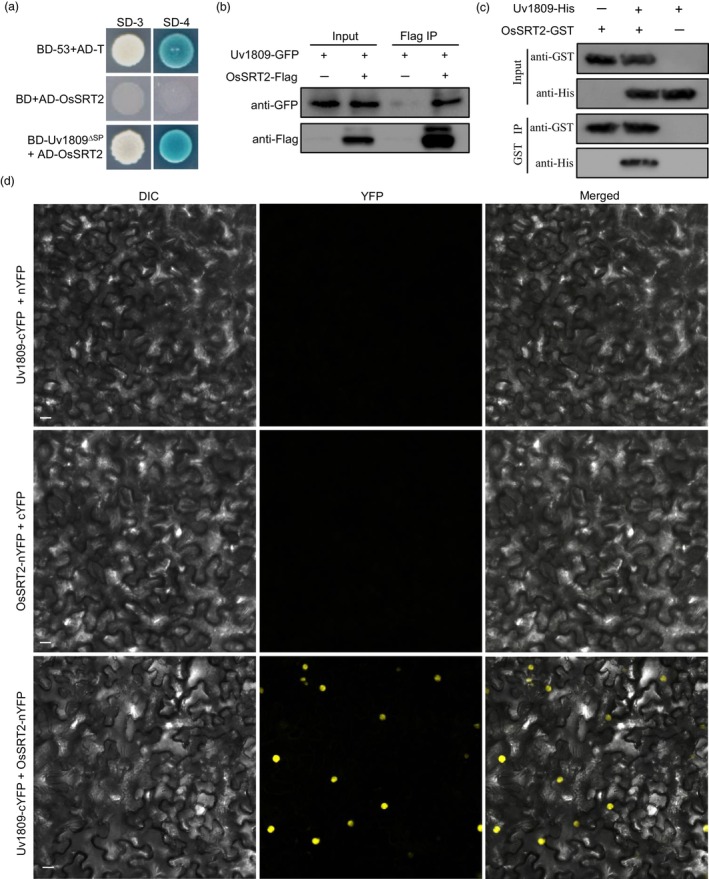
Uv1809 physically interacts with OsSRT2. (a) Y2H analysis of the interaction between Uv1809 and OsSRT2. The interaction between BD‐53 and AD‐T was taken as the positive control, BD and AD‐OsSRT2 was taken as the negative control. SD‐3, SD‐Trp‐Leu‐His; SD‐4, SD‐Trp‐Leu‐His‐Ade; BD, pGBKT7; AD, pGADT7. (b) *In vivo* Co‐IP of Uv1809 interacts with OsSRT2. Co‐IP was performed on extracts of *Nicotiana benthamiana* leaves by co‐expression of OsSRT2‐Flag and Uv1809‐GFP. Uv1809‐GFP and OsSRT2‐Flag were detected by western blotting using anti‐GFP and anti‐Flag antibodies, respectively. (c) A GST pull‐down assay was used to detect the interaction between Uv1809^∆SP^‐His and OsSRT2‐GST. Uv1809 and OsSRT2 were fused to His and GST tags, respectively, and expressed in *Escherichia coli*. OsSRT2‐GST or GST‐bound resin was incubated with *E*. *coli* crude extracts containing Uv1809^∆SP^‐His and analysed by western blotting. Uv1809^∆SP^‐His and OsSRT2‐GST were detected using anti‐His and anti‐GST antibodies, respectively. (d) BiFC assays for the interaction between Uv1809 and OsSRT2. *N*. *benthamiana* leaves were infiltrated with a mixture of *Agrobacterium tumefaciens* strains co‐expressing the OsSRT2‐nYFP and Uv1809‐cYFP constructs. YFP signals were observed at 2 dpi. Infiltration with *Agrobacterium* co‐expressing the OsSRT2‐nYFP and cYFP, nYFP and Uv1809‐cYFP constructs were used as the negative control. No YFP signals was observed in these negative controls. Scale bar = 20 μm.

To further characterize its function during plant infection, we performed a yeast two‐hybrid screen with Uv1809 as bait against a cDNA library constructed from RNA extracted from *U*. *virens*‐infected rice spikelets, leading to the identification of 25 putative Uv1809‐interacting proteins (Table S1). We showed that the rice histone deacetylase OsSRT2 is a target of Uv1809 and confirmed the interaction between Uv1809 and full‐length OsSRT2 by yeast two‐hybrid (Figure 3a). To validate the interaction *in vivo*, we next performed a co‐immunoprecipitation (Co‐IP) assay on *N*. *benthamiana* leaves transiently co‐infiltrated with *OsSRT2‐Flag* and *Uv1809‐GFP*. In this assay, Uv1809 was immunoprecipitated by OsSRT2 (Figure 3b). Next, we performed *in vitro* pull‐down assays with recombinant OsSRT2‐GST (glutathione S‐transferase) and Uv1809^ΔSP^‐His (His tag) proteins purified from *E*. *coli*. We detected Uv1809^ΔSP^‐His from protein samples pulled down with OsSRT2‐GST loaded onto glutathione beads (Figure 3c), suggesting that Uv1809^ΔSP^‐His and OsSRT2‐GST interact *in vitro*. Finally, we transiently co‐infiltrated *N*. *benthamiana* leaves with *Uv1809‐cYFP* and *OsSRT2‐nYFP* fusion constructs for bimolecular fluorescence complementation (BiFC) analysis. We observed YFP fluorescence in the nucleus of *N*. *benthamiana* leaf epidermal cells (Figure 3d). We then attempted to delineate the interaction interface by testing two Uv1809 fragments (Uv1809^23–245^ and Uv1809^246–391^) for interaction with OsSRT2. Only Uv1809^246–391^ fragments interacted with OsSRT2 in Y2H, Co‐IP, and BiFC assays (Figure S8a–c). These results suggest that Uv1809 interacts with OsSRT2 in the nucleus of plant cells, Uv1809^246–391^ is key region for interacting with OsSRT2.

### OsSRT2 negatively regulates broad‐spectrum resistance against rice pathogens

To explore the function of OsSRT2 in resistance against *U*. *virens*, we determined the transcript levels of *OsSRT2* during infection by RT‐qPCR. *OsSRT2* transcript levels were significantly upregulated during infection, peaking at 6 dpi (Figure S9a). This result suggests that *OsSRT2* expression is induced by *U*. *virens* infection and that *OsSRT2* might negatively regulate rice resistance to *U*. *virens*.

**Figure 4:**
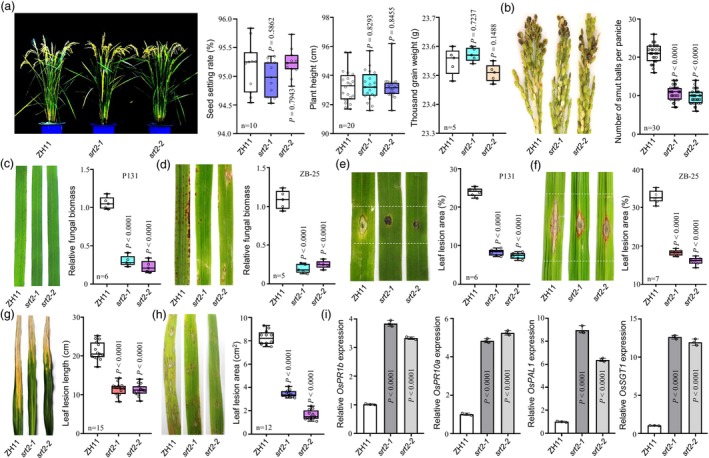
*OsSRT2* negatively regulates rice broad‐spectrum resistance against rice pathogens. (a) Morphology and agronomic traits of the wild‐type ZH11 and *ossrt2* mutants rice lines at mature stage following growth in field conditions. (b) Left: Resistance assays of ZH11 and *ossrt2* mutants rice lines against *Ustilaginoidea virens* HWD‐2 at 25 dpi. Right: Numbers of rice smut balls were calculated in resistance assays. (c) Left: Disease symptoms of ZH11 and *ossrt2* mutants rice lines after spray inoculation with *Magnaporthe oryzae* P131 at 7 dpi. Right: Relative fungal biomass was determined using RT‐qPCR for the *M*. *oryzae Pot2* gene normalized to rice *OsUBQ1*. (d) Disease symptoms (Left) and the relative fungal biomass (Right) of ZH11 and *ossrt2* mutants rice lines after spray inoculation with *M*. *oryzae* ZB‐25 at 7 dpi. (e) Disease symptoms (Left) and leaf lesion area (Right) of ZH11 and *ossrt2* mutants rice lines were spot inoculated with spore suspensions of *M*. *oryzae* P131 at 7 dpi. (f) Disease symptoms (Left) and leaf lesion area (Right) of ZH11 and *ossrt2* mutants rice lines after spot‐inoculation with *M*. *oryzae* ZB‐25 at 7 dpi. (g) Left: Disease symptoms of ZH11 and *ossrt2* mutants rice lines after inoculated with *Xoo* PXO99 at 14 dpi. Right: Lesion lengths on rice leaves of ZH11 and *ossrt2* mutants rice lines after inoculated with *Xoo* PXO99 at 14 dpi. (h) Disease symptoms at 3 dpi of ZH11 and *ossrt2* mutants rice lines after inoculation with *Rhizoctonia solani* HG81. Right: Leaf lesions on ZH11 and *ossrt2* mutants rice lines after inoculation with *R*. *solani* HG81 at 3 dpi. (i) RT‐qPCR analysis of defence‐related genes at 1 dpi in ZH11 and *ossrt2* mutants rice lines inoculated with *U*. *virens*. Data are presented as mean ± SD (*n* = 3 unless otherwise indicated). The *P* values were determined by unpaired *t*‐tests compared with the wild‐type ZH11.

To examine OsSRT2 localization in plant cells, we assessed the subcellular localization of an OsSRT2‐GFP fusion protein in *N*. *benthamiana* leaves. We determined that OsSRT2 is localized to the nucleus (Figure S9b). To explore the function of OsSRT2 in resistance against rice pathogens, we generated *OsSRT2* knockout mutants (*ossrt2*) using Clustered Regularly Interspaced Short Palindromic Repeats/CRISPR Associated Protein 9 (CRISPR/Cas9) gene editing (Figure S10). The morphology of *ossrt2* rice lines was similar to that of wild‐type ZH11 rice plants (Figure 4a, Figure S11). After inoculation with a mycelium/spore suspension of *U*. *virens* strain HWD‐2, *ossrt2* rice lines produced far fewer smut balls than ZH11 lines (Figure 4b). We then evaluated the *ossrt2* rice lines for resistance against the rice blast fungus *M*. *oryzae* strain P131 or ZB‐25. The *ossrt2* rice lines were more resistant to *M*. *oryzae* than the ZH11 lines (Figure 4c–f). Inoculation of *ossrt2* and ZH11 rice lines with *Xoo* strain PX099 using the scissor‐clipping method produced similar results, with lesions being ~75% shorter on the *ossrt2* rice lines than on the ZH11 lines at 14 dpi (Figure 4g). Likewise, *ossrt2* rice lines were more resistant to infection by *R*. *solani* strain HG81 than ZH11 lines at 3 dpi (Figure 4h). Interestingly, we found that the transcription levels of defence‐related genes (*OsPR1b*, *OsPR10a*, *OsPAL1*, and *OsSGT1*) were significantly increased in the *ossrt2* plants after inoculation of *U*. *virens* at 1 dpi (Figure 4i), indicating that the upregulation of these genes is associated with *OsSRT2*. Overall, these results indicate that *OsSRT2* negatively regulates rice resistance to multiple diseases.

### Uv1809 increases the deacetylation activity of OsSRT2

**Figure 5:**
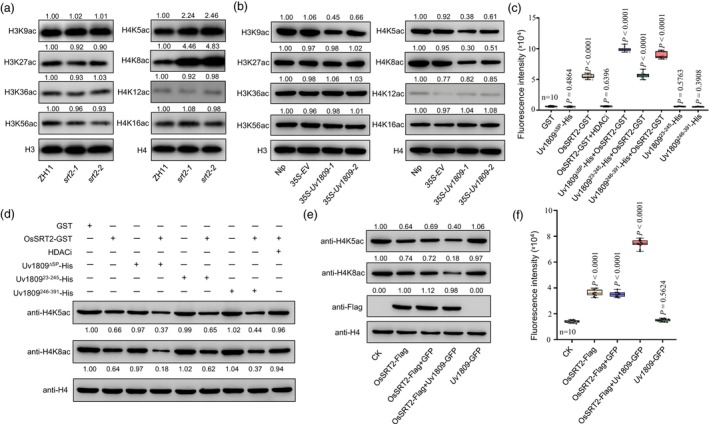
Uv1809 modulates histone deacetylation activity of OsSRT2. (a) The levels of H3K9ac, H3K27ac, H3K36ac, H3K56ac, H4K5ac, H4K8ac, H4K12ac and H4K16ac in the wild‐type ZH11 and *ossrt2* mutants rice spikelets were detected by western blotting. Relative quantified signals of each band are indicated with the first ZH11 loading set as 1.00. (b) Relative the levels of H3K9ac, H3K27ac, H3K36ac, H3K56ac, H4K5ac, H4K8ac, H4K12ac and H4K16ac in Nip, *35S‐EV* and *35S‐Uv1809* transgenic rice plants as detected by western blotting. (c) *In vitro* lysine deacetylation activity of OsSRT2 by fluorometric assays. A HDAC Assay Kit (Fluorescent) (Active Motif) was used to determine HDAC activity of purified GST, Uv1809^∆SP^‐His, Uv1809^23–245^‐His, Uv1809^246–391^‐His, OsSRT2‐GST, OsSRT2‐GST and Uv1809^∆SP^‐His, OsSRT2‐GST and Uv1809^23–245^‐His, OsSRT2‐GST and Uv1809^246–391^‐His, HADCs inhibitor Nicotinamide and OsSRT2‐GST proteins. (d) *In vitro* histone H4K5 and H4K8 deacetylation activity of OsSRT2 by immunoblotting. For *in vitro* deacetylation assay, 20 μL rice histone protein, 2 μL the purified GST, Uv1809^∆SP^‐His, Uv1809^23–245^‐His, Uv1809^246–391^‐His, OsSRT2‐GST, OsSRT2‐GST and Uv1809^∆SP^‐His, OsSRT2‐GST and Uv1809^246–391^‐His, OsSRT2‐GST and Uv1809^23–245^‐His, OsSRT2‐GST and HDACi proteins were incubated in 20 μL reaction buffer at 30 °C for 4 h. The reaction products were analysed by western blotting with anti‐H4K5ac and anti‐H4K8ac antibodies. (e) *In vivo* H4K5ac and H4K8ac levels when OsSRT2 and Uv1809 co‐expression in *Nicotiana benthamiana* leaves. OsSRT2‐Flag, GFP, Uv1809‐GFP, Uv1809‐GFP and OsSRT2‐Flag, GFP and OsSRT2‐Flag fusion proteins were expression/co‐expression in *N*. *benthamiana* leaves as detected with anti‐H4K5ac and anti‐H4K8ac antibodies. (f) *In vitro* lysine deacetylation activity of nuclear proteins from (e) by fluorometric assays.

Rice HDACs, including OsHDT701, regulate histone H3 or H4 acetylation levels (Ding *et al*., 2012). We therefore examined whether OsSRT2 affected histone deacetylation. Using histones isolated from the spikelets of *ossrt2* and ZH11 rice lines, we performed an immunoblot analysis using various anti‐H3ac and anti‐H4ac antibodies. We established that levels of H4K5ac and H4K8ac are higher in the *ossrt2* mutant than in ZH11, while levels of other lysine sites on H3 tested were not increased (Figure 5a). Thus, OsSRT2 mainly regulates histone acetylation levels of H4K5 and H4K8 in rice spikelets. We also used various anti‐H3ac and anti‐H4ac antibodies to detect the levels of H3ac and H4ac in *35S‐EV* and *35S‐Uv1809* transgenic rice spikelets. H3K9ac, H4K5ac, and H4K8ac levels in *35S‐Uv1809* transgenic rice lines were markedly lower than in *35S‐EV* transgenic rice lines and Nip rice plants (Figure 5b), this result indicated that Uv1809 might repress the H4K5ac and H4K8ac of rice by affecting OsSRT2. To test this hypothesis, we measured the activity of recombinant OsSRT2‐GST alone or in the presence of Uv1809^ΔSP^‐His, Uv1809^23–245^‐His, or Uv1809^246–391^‐His. First, we confirmed the *in vitro* deacetylation activity of OsSRT2‐GST using a fluorometric assay. Notably, deacetylation activity in the reaction increased when OsSRT2‐GST was incubated with Uv1809^ΔSP^‐His or Uv1809^246–391^‐His, but not when it was incubated with Uv1809^23–245^‐His or HDACi (Figure 5c). This rise in activity was not associated with the effector itself, as recombinant Uv1809^ΔSP^‐His, Uv1809^23–245^‐His, and Uv1809^246–391^‐His showed no deacetylation activity themselves (Figure 5c). We independently observed decreased levels of H4K5 and H4K8 deacetylation activity *in vitro* by immunoblot analysis of recombinant OsSRT2‐GST incubated with purified rice histones. Furthermore, OsSRT2‐mediated deacetylation of H4K5ac and H4K8ac was enhanced by co‐incubation with recombinant Uv1809^ΔSP^‐His or Uv1809^246–391^‐His (Figure 5d). In order to test the effect of Uv1809 on the histone deacetylase activity of OsSRT2 in *planta*, we transiently expressed OsSRT2‐Flag, OsSRT2‐Flag and Uv1809‐GFP, OsSRT2‐Flag and GFP, and Uv1809‐GFP in *N*. *benthamiana* leaves. The nuclear proteins were then extracted and the H4K5 and H4K8 acetylation levels were evaluated. As shown in Figure 5e, OsSRT2‐Flag and Uv1809‐GFP co‐expression reduced the acetylation levels of histones H4K5 and H4K8. In contrast, Uv1809‐GFP or GFP alone did not reduce the acetylation levels of H4K5 and H4K8 (Figure 5e). Accordingly, OsSRT2‐Flag and Uv1809‐GFP co‐expression increased the deacetylation activity of these nuclear proteins and were determined using a fluorometric assay (Figure 5f). Taken together, these results indicate that Uv1809 enhances OsSRT2‐mediated histone deacetylation *in vitro* and *in vivo*.

### OsSRT2 suppresses rice immunity by modulating H4K5ac‐ and H4K8ac‐marked genes

**Figure 6:**
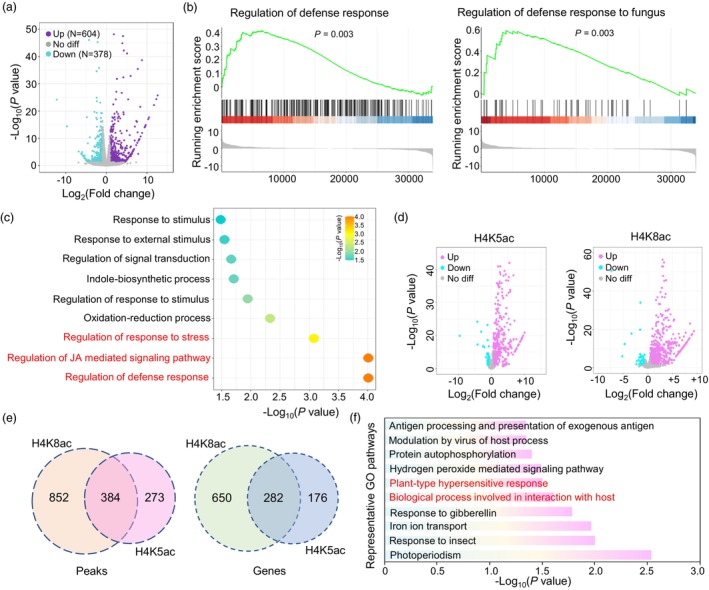
RNA‐seq and ChIP‐seq data analysis of *ossrt2* and wild‐type rice spikelets. (a) Volcano plots of differential transcript levels in *ossrt2* relative to wild type. Purple plots represent upregulated genes (Fold change > 2, *P* value < 0.05); cyan plots represent downregulated genes (Fold change > 2, *P* value < 0.05); grey plots represent genes with no significant difference. (b) Representative GSEA enriched pathways in *ossrt2*. (c) GO pathway analysis of the genes (*n* = 604) that were upregulated in *ossrt2* mutants. (d) Peaks with H4K5ac and H4K8ac level changes in *ossrt2* compared with wild type. Peaks with reduced H4K5ac or H4K8ac in the mutant are shown in cyan, and those with gained H4K5ac or H4K8ac are in pink (Fold change > 1.5, *P* < 0.05). (e) Venn diagrams of upregulated H4K5 and H4K8 acetylation peaks (left) or genes (right) in *ossrt2*. (f) GO pathways found in both H4K5ac and H4K8ac significantly upregulated genes in *ossrt2* versus wild type.

To identify genes that could be regulated by OsSRT2 in rice spikelets, we performed RNA sequencing (RNA‐Seq) to identify genes that were differentially expressed in *ossrt2* mutant rice spikelets relative to wild‐type ZH11 rice spikelets. Three biological replicates were performed per sample (Figure S12a). A total of 604 and 378 genes were upregulated and downregulated, respectively, to >2‐fold (*P* < 0.05) in *ossrt2* compared to wild type (Figure 6a; Table S2).

Gene set enrichment analysis (GSEA) was performed to investigate pathways associated with the *ossrt2* mutation (Figure 6b). Regulation of defence response to fungus and regulation of defence response pathways were significantly enriched in our dataset, suggesting that genes in these pathways were upregulated in *ossrt2*. Furthermore, gene ontology (GO) pathway enrichment of significantly upregulated genes in *ossrt2* also revealed that regulation of defence response, regulation of jasmonic acid‐mediated signalling pathway, and regulation of response to stimulus pathways were enriched (Figure 6c). To confirm the gene expression patterns, we selected eight genes for RT‐qPCR analysis. The expression pattern of each upregulated or downregulated gene was consistent with the RNA‐Seq data (Figure S13). Collectively, these results indicate that mutation of *OsSRT2* might promote defence pathways in rice spikelets and confer the mutants with resistance to pathogen infections.

To further investigate the gene regulation mechanisms of OsSRT2 during infection, we performed ChIP‐seq to compare genome‐wide profiles of H4K5ac and H4K8ac in rice spikelets from wild‐type and *ossrt2* plants. For each ChIP‐seq experiment, two biological replicates (Figures S12b, S14) were carried out. From the two replicates, 44 392 (38 761 marked genes) H4K5ac and 44 352 (38 628 marked genes) H4K8ac peaks were identified in wild‐type rice spikelets. These two histone modifications showed a similar genomic distribution and were highly enriched at the transcription start site (Figure S15a, b). The genomic patterns of H4K5ac and H4K8ac were comparable (Figure S15c, d) and showed a moderately significant connection with gene transcription (Figure S15e). We identified a total of 657 peaks (458 genes) with elevated (>1.5‐fold, *P* < 0.05) H4K5ac levels and 316 peaks (249 genes) with reduced H4K5ac levels (>1.5‐fold, *P* < 0.05) in the mutant (Figure 6d, Table S3). Comparative analysis of H4K8ac revealed that a total of 1236 peaks (932 genes) showed a significant increase in H4K8ac depositions in *ossrt2*, whereas only 74 peaks (61 genes) showed a substantial decrease in H4K8ac depositions (>1.5‐fold, *P* < 0.05) in *srt2* (Figure 6d, Table S4). These findings were consistent with the immunoblotting estimates of H4K5ac and H4K8ac levels in *ossrt2* shown in Figure 5a.

To investigate whether H4K5ac and H4K8ac co‐regulate a subset of genes in the *ossrt2* mutant, the overlap of H4K5ac and H4K8ac peaks (genes) was analysed. The results showed that 58.4% (61.2%) of the peaks (genes) with upregulated H4K5ac overlapped with those of H4K8ac in *ossrt2* (Figure 6e), indicating that the *ossrt2* mutation had a similar effect on both H4K5ac and H4K8ac deposition. To identify the metabolic pathways regulated by H4K5 and H4K8 hyper‐acetylated genes (*N* = 282) in *ossrt2*, GO pathway enrichment analysis was performed. We identified 62 GO terms as being associated with H4K5 and H4K8 hyper‐acetylated genes, and these terms were involved with a variety of biological processes, including defence‐related pathways such as plant‐type hypersensitive response and modulation by virus of host process (Figure 6f, Table S5).

**Figure 7:**
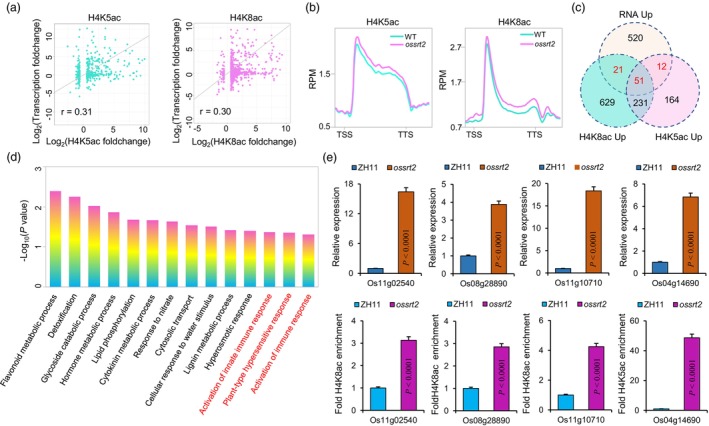
Role of H4K5ac and H4K8ac in gene expression regulation in *ossrt2* rice spikelets. (a) Correlation analysis of expression changes and H4K5ac or H4K8ac changes in *ossrt2* versus wild type. Person Correlation Coefficient was shown. (b) Metaplots of H4K5 or H4K8 acetylation ChIP‐seq reads in transcriptionally upregulated genes. TSS, transcriptional start site. TES, transcriptional end site, RPM, reads count per million mapped reads. (c) Venn diagrams of H4K5 and H4K8ac hyper‐acetylated genes, and transcriptionally upregulated genes in *ossrt2*. (d) Representative GO pathways of H4K5ac, H4K8ac, and transcriptionally upregulated genes (*N* = 84) in *ossrt2*. (e) RT‐qPCR and ChIP‐qPCR analysis of H4K5ac‐ or H4K8ac‐marked defence‐related genes in ZH11 and *ossrt2* rice spikelet*s*. Asterisks indicate statistically significant differences compared to ZH11 at *P* < 0.05.

To investigate the relationship between the transcriptome and histone acetylation in rice spikelets, we first examined the correlation between the dynamic changes in H4K5/8ac and gene transcription in *ossrt2* mutants. As expected, a moderate level of positive correlation (*r* = 0.30/0.31) between H4K5/8ac and gene expression changes was observed in the mutant (Figure 7a), which was in line with their moderately significant connection with gene transcription (Figure S15e). Furthermore, those upregulated genes displayed concurrent increases in both H4K5ac and H4K8ac in the *ossrt2* mutant (Figure 7b) compared to the wild type. To identify the genes regulated by H4K5/8ac hyper‐acetylation and transcriptional upregulation in the mutant, we analysed the overlap between H4K5/8ac hyper‐acetylation and transcriptionally upregulated genes. As shown in our Venn diagram, 84 genes were both transcriptionally upregulated and H4K5/8ac hyper‐acetylated in *ossrt2* mutants (Figure 7c, Table S6). To establish the pathways in which these genes were enriched, we performed GO pathway analysis and identified 56 GO terms. Among the biological processes highlighted by GO analysis, plant defence‐related pathways were found to be significant, including hyperosmotic response, activation of innate immune response, and plant‐type hypersensitive response (Figure 7d, Table S7). We analysed precipitated chromatin fragments by ChIP‐qPCR with primer sets designed to amplify the promoter regions of selected genes. Compared to the wild type, H4K5ac and H4K8ac levels were significantly increased at the promoters of these tested genes in the *ossrt2* mutant. Therefore, increased H4K5ac or H4K8ac levels on histones at the promoter regions of rice defence‐related genes lead to more active transcription (Figure 7e). Collectively, our results imply that the deacetylation of H4K5/8 by OsSRT2 suppresses the transcription of plant defence‐related genes, leading to a decrease in rice immunity.

## Discussion

**Figure 8:**
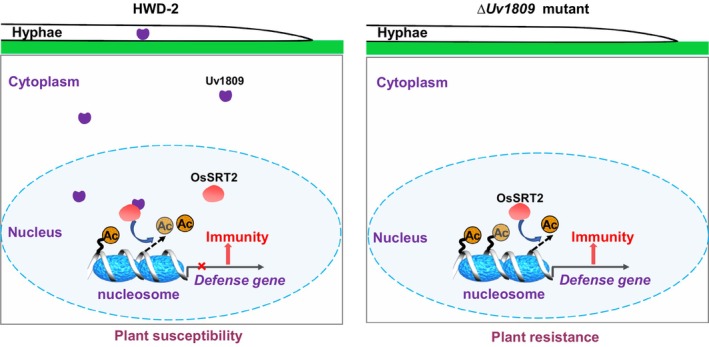
A working model illustrating how Uv1809 manipulates histone deacetylase OsSRT2 to suppress rice immunity during *Ustilaginoidea virens* infection. During infection, *U*. *virens* effector Uv1809 is secreted and translocated into host cells, and then physically interacts with OsSRT2. Uv1809 disrupts host immunity by recruiting enhancing OsSRT2‐modulated deacetylation, thereby reducing the levels of H4K5ac and H4K8ac in rice plants and interfering with defence gene activation.

Previous studies have revealed that acetylation plays an essential role in plant defences during plant–pathogen interactions (Gómez‐Díaz *et al*., 2012; Parker *et al*., 2022). Recent studies have shown that fungal effectors can regulate plant immunity by interacting with HATs and HDACs, thereby affecting histone acetylation to regulate the expression of defence genes and interfere with plant immunity (Kong *et al*., 2017; Li *et al*., 2018). In this study, we showed that the secreted effector Uv1809 from *U*. *virens* physically interacts with histone deacetylase OsSRT2, enhancing its histone deacetylase activity and reducing H4K5 and H4K8 acetylation levels at the promoter of defence‐related genes. Subsequently, these lower H4K5 and H4K8 acetylation levels result in lower expression of defence genes and compromise rice resistance to pathogens (Figure 8). Interestingly, we established that Uv1809^246–391^ localizes to the nucleus and contains numerous repetitive sequences. This region of Uv1809 is key for inhibiting Bax‐induced cell death, interacting with OsSRT2, and enhancing its enzyme activity. Thus, the function of Uv1809^246–391^ in the nucleus merits further analysis.

The use of plant immunity to improve disease resistance is the most cost‐effective and environmentally safe means of ensuring food security. In general, to exploit plant immunity, key genes involved in immune regulation need to be identified. In recent years, the development of cutting‐edge omics analysis and CRISPR/Cas9‐mediated gene editing technology has promoted the discovery of disease‐resistance genes and allowed for the editing of disease‐susceptibility genes, thus accelerating the development of sustainable and robust agricultural production. In rice, 18 HDACs have been identified, including 14 RPD3/HDA1 (Reduced Potassium Dependence 3/Histone Deacetylase 1) members, two SIR2 (Silent Information Regulator 2) members, and two HD (Histone Deacetylase 2) members (Chen *et al*., 2020a). Among them, HDT701, HDA701, and HDA705 have been reported to negatively regulate rice blast, RFS, and/or bacterial blight disease resistance (Chen *et al*., 2021, 2022a; Ding *et al*., 2012). Sirtuin‐like deacetylases are crucial regulators of a variety of biological processes, such as metabolism, organ development, and plant responses to oxidative and abiotic stress (Tang *et al*., 2022; Zhang *et al*., 2017, 2018). Here, we showed that this novel SRT2 negatively regulates broad‐spectrum resistance against multiple rice pathogens, making it a prime candidate gene for breeding programs aimed at increasing resistance to rice pathogens.

In the *osssrt2* mutants, some defence‐related genes were upregulated (Figure S13), including those encoding transcription factors, receptor‐like kinases, and disease‐resistance proteins. Overexpression of the transcription factor OsMYB30 (Os09g26170) enhances resistance to rice blast, while knockdown of OsMYB30 decreases resistance to rice blast, indicating that OsMYB30 positively regulates immune responses (Li *et al*., 2020). Overexpression of the transcription factor OsWRKY89 (Os11g02520) led to increased salicylic acid levels, which enhanced resistance to rice blast (Wang *et al*., 2007). OsPR1b (Os01g28450), a key PR gene in rice, was significantly upregulated in the *osssrt2* mutant (Mitsuhara *et al*., 2008). A respiratory burst oxidase homologue (Rboh) functions in the production of reactive oxygen species, and OsRbohD (Os05g38980) participates in the immune response process (Yoshiaki *et al*., 2005). Thus, genome editing of *OsSRT2* directly or indirectly upregulates the expression of key defence‐related genes (such as *OsMYB30*, *OsWRKY89*, *OsRbohD*, and *OsPR1b*) to enhance resistance to rice pathogens.

Chitin is a major component of the fungal cell wall and acts as a PAMP elicitor that triggers PTI in several plant species (Tariqjaveed *et al*., 2021). PAMP elicitors trigger a series of defence responses, including ROS generation and the expression of defence‐related genes. Interestingly, we found that the ROS generation was significantly increased after chitin treatment in the *ossrt2* plants relative to ZH11 plants (Figure S16), *OsRbohD* was significantly upregulated in the *osssrt2* mutant, revealing that *OsSRT2* affects ROS generation by regulating the expression of *OsRbohD*. OsSGT1 functions through the RAR1‐SGT1‐HSP90 cytosolic defensome complex and mediates accumulation of the ROS generated by NADPH oxidases (Azevedo *et al*., 2002). So, we speculate that the *OsSRT2* function on key PTI‐related components. Intriguingly, the expression of defence‐related genes *OsPR1b*, *OsPAL1*, *OsWRKY13*, and *OsAOS2* was significantly downregulated in the *35S‐Uv1809* transgenic rice plants during infection by *U*. *virens*. Accordingly, the transcription levels of defence‐related genes (*OsPR1b*, *OsPR10a*, *OsPAL1*, and *OsSGT1*) were significantly increased in the *ossrt2* plants. Our data support the hypothesis that Uv1809 suppresses the activation of defence‐related genes by affecting OsSRT2 during infection of *U*. *virens*, thereby compromising plant immunity.

In this study, we established that OsSRT2 suppresses rice immunity by modulating H4K5ac‐ and H4K8ac‐marked genes. Protein phosphorylation by protein kinases transmits defence signals to downstream proteins, such as WRKY transcription factors, which have been extensively characterized in plant defence responses (Eulgem and Somssich, 2007; Lin *et al*., 2022; Park *et al*., 2012; Wani *et al*., 2021). We also discovered that the transcription and histone acetylation levels of defence‐related genes, such as WRKY50 (Os11g02540) and protein kinases (Os11g10710 and Os08g28890), were increased in *srt2* mutant plants, implying that SRT2 may regulate plant resistance to pathogens through a specific group of defence‐related genes. The systemic acquired resistance (SAR) is effective against a broad range of pathogens, which is accompanied by the de‐novo synthesis of PR proteins. In Arabidopsis, AtWRKY50 as an activator of *PR1* gene expression, *PR1* is a SA inducible marker gene for SAR (Hussain *et al*., 2018). These findings show that SRT2 negatively regulates innate immunity in rice by modulating the levels of histone H4 acetylation in defence‐related genes.

Chromatin structure and transcriptional activity of genes are regulated by PTMs on histones such as methylation, acetylation, ubiquitination, and phosphorylation (Alvarez *et al*., 2010). Based on previous studies, PTMs of histones, including 2‐hydroxyisobutyrylation and crotonylation, also play important roles in gene expression (Chen *et al*., 2021). OsSRT2 is an “eraser” of these new PTMs, which might regulate the expression of defence‐related genes. In Arabidopsis, the acetylation level of different histone sites was not significantly different between *srt2‐1* mutants and wild‐type plants, but AtSRT2 can specifically deacetylate lysine residues at H4K8ac, H3K14ac, and H3K9ac loci *in vitro* (Tang *et al*., 2022). Interestingly, except H4K5ac and H4K18ac, heterologous expression of Uv1809 also strongly reduced the level of H3K9ac in rice (Figure 5b). Given that Sirtuin has been reported to possess H3K9ac deacetylation activity in other species (Tang *et al*., 2022), it's plausible that this activity might not be as robust in rice panicle. Nevertheless, it's not excluded that Uv1809 might potentially modulate other histone deacetylases to some extent. However, during infection by *U*. *virens*, OsSRT2 may regulate other histone acetylation sites to modulate the expression of defence‐related genes, thereby negatively regulating rice immunity. Regardless, the insights gleaned from our study indicate that Uv1809 specifically enhances the activity of OsSRT2, which predominantly targets the H4K5ac and H4K8ac histone marks in rice panicle tissue.

The development of durable and broad‐spectrum resistance is an economical and sustainable approach to control crop diseases for agricultural production. Recently, the RNAi‐based approach host‐induced gene silencing (HIGS) was developed as an alternative strategy to control fungal diseases. In this strategy, siRNAs targeting selected genes of the invading pathogen are produced by transgenic host plants to silence fungal genes during infection (Chen *et al*., 2022c; Li *et al*., 2021a,b; Machado *et al*., 2018; Nowara *et al*., 2010). For example, HIGS of *FgChs3b*, *FgCYP51*, *FgSGE1*, *FgFGP1*, or *FgSTE12* conferred resistance to *Fusarium* head blight fungus *Fusarium graminearum* in wheat (*Triticum aestivum*) (Cheng *et al*., 2015; Koch *et al*., 2013; Wang *et al*., 2020a,b). Similarly, HIGS of *PsCPK1* or *PsFUZ7* enhanced wheat resistance to the stripe rust fungus *Puccinia striiformis* f. sp. *tritici* (Qi *et al*., 2018; Zhu *et al*., 2017). Other successful examples of engineered resistance have also been demonstrated in rice against rice blast or RFS (Chen *et al*., 2022c; Guo *et al*., 2019). Our study now adds HIGS against *Uv1809* as an effective strategy to reduce the severity of *U*. *virens* infection in rice, providing a reference for the development of stable transgenic plants using a HIGS‐based strategy to enhance rice resistance.

In summary, we identified an important secreted effector, Uv1809, which interacts with the histone deacetylase OsSRT2. Uv1809 enhances OsSRT2‐mediated histone deacetylation, thus modulating the expression of defence‐related genes in the host. Our study demonstrates that a fungal effector from *U*. *virens* targets a HDAC to suppress plant immunity. Strikingly, stable transgenic rice lines expressing *Uv1809*‐silencing RNAs and *ossrt2* mutant rice lines showed increased broad‐spectrum resistance to *U*. *virens* and other rice pathogens, pointing to novel strategies for protecting rice crops.

## Experimental procedures

### Plant materials and bacterial and fungal strains

*Oryza sativa* cvs. ‘Wanxian‐98’, ‘Zhonghua 11’ [ZH11] and ‘Nipponbare’, and *Nicotiana benthamiana* plants were grown in a glasshouse. Transgenic rice plants with host‐induced gene silencing (HIGS) of *Uv1809* or heterologous expression of *Uv1809* (*35S‐Uv1809*) or the empty vector (*35S‐EV*) were generated in the Nipponbare background. The *ossrt2* knockout rice lines were generated in the ZH11 background using CRISPR/Cas9 technology. Rice transformation was performed by Wuhan Tianwen Biotechnology Co., Ltd (Wuhan, China).

Rice plants were inoculated with the fungal strains *Magnaporthe oryzae* P131 or ZB‐25, *Xanthomonas oryzae* pv. *oryzae* (*Xoo*) PXO99, *Rhizoctonia solani* HG81, and *U*. *virens* HWD‐2.

### Fungal inoculation assays

Rice plants at the booting stage were inoculated with *U*. *virens* HWD‐2 mycelial/spore suspensions (1 × 10^6^ conidia/mL) using a syringe, and the number of false smut balls was counted 21–25 dpi (Chen *et al*., 2020b). For spray‐inoculations, the leaves of four‐week‐old rice plants were sprayed with *M*. *oryzae* P131 or ZB‐25 conidial suspensions (1 × 10^6^ conidia/mL) in 0.025% (v/v) Tween 20, and the disease lesions were examined at 7 dpi. For spot‐inoculations, the leaves of 4‐week‐old plants were punctured with a needle, and a droplet of 10 μL *M*. *oryzae* P131 or ZB‐25 conidial suspension (1 × 10^5^ conidia/mL) was placed at each wound site. Rice plants were inoculated with *Xoo* PXO99 at the booting stage using the leaf clipping method, and the lesion lengths were scored at 14 dpi. The leaves of four‐week‐old rice plants were inoculated with *R*. *solani* HG81, and the lesion areas were scored at 3 dpi and calculated using ImageJ software. All inoculation experiments were repeated three times.

### Gene deletion and complementation in *Uv1809*

*U*. *virens* transformation was carried out as previously described (Chen *et al*., 2020b). Briefly, about 1.2‐kb of the downstream and upstream flanking sequences of *Uv1809* were ligated with the knockout vector pGKO. An approximately 3.5‐kb complementation fragment was cloned into the complementation vector pNeo3300III. The EHA105 Agrobacterium strain carrying the pGKO‐*Uv1809*, pNeo3300III‐*Uv1809* ^*∆SP*^, or pNeo3300III‐*Uv1809* vectors was transformed with the ATMT method. Transformed strains were confirmed by PCR analysis and Southern blot. Southern blot was performed using the Amersham Gene Images Alkphos Direct Labelling and Detection System (GE Healthcare, Little Chalfont, UK).

### Small RNA sequencing and FISH assays

Total RNA was extracted from the infected spikelets of *Uv1809*HIGS‐L1 transgenic rice lines and then used for small RNA‐Seq on an Illumina HiSeq platform by Wuhan IGENEBOOK Biotechnology Co., Ltd (Wuhan, China). Unique reads were mapped to the corresponding target fragments of *Uv1809*. For FISH assays, a 21‐bp oligonucleotide probe (GTCAATCTCAGCCCTGGCTCG), which was the most common siRNA sequence identified by small RNA‐Seq, was labelled using digoxin (DIG). Infected spikelets at 6 dpi were placed in fixative solution (Servicebio, Wuhan, China) for 6 h and embedded in paraffin. Paraffin sections were dewaxed with a xylene and rehydrated with a gradient alcohol, then digested with protease K (20 μg/mL) at 37 °C for 30 min. After washing with PBS, the prehybridization solution was added to the mixture and incubated at 37 °C for 1 h. After incubation, sections were poured the prehybridization solution, dropped the hybridization solution containing DIG‐labelled probe and hybridized at 37 °C for 16 h. After hybridization, it was washed and sealed, and then, anti‐digoxin labelled peroxidase (anti‐DIG‐HRP) was added to incubate at 37 °C for 1 h, then wash three times with PBS and add Alexa Fluor 594 Tyramide (ThermoFisher Scientific, MA, USA). The fluorescence of the DIG‐labelled siRNA was analysed with a Zeiss LSM 510 Meta confocal microscope (LSCM) (Carl Zeiss, Jena, Germany). DAPI (4′‐6‐diamidino‐2‐phenylindole) was used for nuclear staining.

### Agrobacterium‐mediated infiltration assays and confocal microscopy

The cDNA sequence of *Uv1809* was ligated with pVX to obtain the recombinant vector pVX‐*Uv1809*. pVX‐*Uv1809*, pVX‐*Bax*, and empty pVX were electroporated into *Agrobacterium tumefaciens* (Agrobacterium) strain EHA105. Agrobacterium cultures containing either the pVX‐*Uv1809* or pVX vectors were re‐suspended in infiltration buffer (10 mm MgCl_2_, 0.5 mm MES and 0.2 mm acetylacetone) and infiltrated into the abaxial side of 4‐week‐old *N*. *benthamiana* leaves. 24 h later, Agrobacterium carrying the pVX‐*Bax* vector was infiltrated at the same position, and programmed cell death (PCD) was assessed at 4 dpi. For subcellular localization assays, Agrobacterium carrying the variant constructs pCNG‐*Uv1809* or pCNG‐*OsSRT2* was infiltrated into *N*. *benthamiana* leaves, and the fluorescence signal of the infiltrated areas was analysed with a LSCM at 2 dpi. After *N*. *benthamiana* leaves were treated with 1 m NaCl for 10 min, plasmolysis was observed.

### Uv1809 secretion assay

The signal peptide (SP) of Uv1809 was validated using a yeast invertase secretion assay (Jacobs *et al*., 1997). Briefly, the 66‐bp SP sequence was ligated with the pSUC2 vector. The constructs pSUC2‐*Uv1809SP*, pSUC2‐*Avr1bSP* (positive control) and pSUC2 (negative control) were individually transformed into yeast (*Saccharomyces cerevisiae*) strain YTK12. Transformants were used for invertase secretion assays in SD‐Trp or YPRAA medium. The enzymatic activity of invertase was detected by reducing TTC (2,3,5‐triphenyltetrazolium chloride) to the insoluble red precipitated TPF (1,3,5‐triphenylformazan).

### Yeast two‐hybrid (Y2H) assay

The cDNA sequence of *Uv1809* ^*∆SP*^ (without the SP) was ligated with pGBKT7 to generate the bait vector pGBKT7‐*Uv1809* ^*∆SP*^. The mRNA from rice spikelets was used to construct a cDNA library in the prey vector pGADT7. Library screening for Uv1809 interactors was carried out as described previously (Chen *et al*., 2020b). For interaction analysis of Uv1809 and OsSRT2 in yeast, the vectors pGBKT7‐*Uv1809* ^*∆SP*^ and pGADT7‐*OsSRT2*, pGBKT7‐*Uv1809* ^*23*–*245*^ and pGADT7‐*OsSRT2*, or pGBKT7‐*Uv1809* ^*246*–*391*^ and pGADT7‐*OsSRT2* were co‐transformed into the Y2H Gold strain. Positive clones were selected on SD‐Trp‐Leu‐His medium, and further confirmed on SD‐Trp‐Leu‐His‐Ade medium containing X‐α‐Gal.

### Co‐immunoprecipitation (Co‐IP) assays

The cDNA sequences of *Uv1809* and *OsSRT2* were ligated with the vectors pCNG (GFP tags) and pCNF (Flag tags) and then introduced individually into Agrobacterium strain EHA105. Agrobacterium carrying the pCNG‐*Uv1809* and pCNF‐*OsSRT2* vectors were co‐infiltrated into *N*. *benthamiana* leaves. Total proteins were extracted from *N*. *benthamiana* leaves at 2 dpi and incubated with Anti‐Flag M2 affinity gel (Yeasen Biotech, Shanghai, China). Proteins eluted from gels were analysed by immunoblotting with anti‐GFP or anti‐Flag antibodies (ABclonal, Wuhan, China).

### GST pull‐down assays

The cDNA sequences of *Uv1809* ^*∆SP*^ and *OsSRT2* were ligated with the vectors pET32a (His tags) and pGEX4T‐2 (GST tags) and then introduced individually into *Escherichia coli* BL21(DE3) cells. The OsSRT2‐GST fusion protein was extracted from *E*. *coli* cells and incubated with 100 μL glutathione‐agarose beads (Yeasen Biotech, Shanghai, China) at 4 °C for 4 h with shaking. After centrifugation at 4 °C, the beads were collected and washed with phosphate buffered saline (PBS) three times. Beads were then incubated with recombinant Uv1809^∆SP^‐His protein at 4 °C for 2 h with shaking and then washed with PBS three times. Beads were boiled for 5 min at 100 °C in 40 μL SDS sample loading buffer, and the proteins were analysed by immunoblotting with anti‐His and anti‐GST antibodies (ABclonal).

### Bimolecular fluorescence complementation assays

The cDNA sequences of *Uv1809* and *OsSRT2* were ligated with the BiFC vectors pCAMBIA1301‐nYFP and pCAMBIA1301‐cYFP, respectively. The resulting constructs encoding the Uv1809‐cYFP and OsSRT2‐nYFP fusion proteins were co‐infiltrated into *N*. *benthamiana* leaves, and the fluorescence signal of the infiltrated areas was analysed with a LSCM at 2 dpi.

### Immunoblot analysis

Proteins were separated by 12% SDS‐PAGE and transferred onto a polyvinylidene fluoride (PVDF) membrane (Merck Millipore, Burlington, MA) using wet transfer at 80 V for 90 min with a BioRad electroblotting apparatus. Membranes were blocked in Tris buffered saline with 0.1% Tween 20 (TBST) containing 5% (w/v) non‐fat dry milk at room temperature for 2 h. Primary antibodies used were anti‐GFP, anti‐Flag, anti‐His, or anti‐GST (ABclonal). The membrane was incubated with primary antibodies in TBST with 5% non‐fat dry milk at room temperature for 2 h with shaking and washed six times (5 min each) with TBST. Next, the membrane was incubated with goat anti‐mouse (ABclonal) secondary antibody in TBST with 5% non‐fat dry milk at room temperature for 1.5 h with shaking. The membrane was washed six times (5 min each) with TBST, and the signals were detected using Pierce ECL Western blotting substrate (Thermo Fisher Scientific) in a ChemiDoc XRS+ system (BioRad, Hercules, USA).

### Histone acetylation assays

Rice spikelets were used to extract histone‐enriched proteins as described previously (Lu *et al*., 2018). Histones were analysed by immunoblotting using anti‐H3 (PTM‐1001), anti‐H3K9ac (PTM‐112), anti‐H3K27ac (PTM‐116), anti‐H3K36ac (PTM‐117), anti‐H3K56ac (PTM‐118), anti‐H4 (PTM‐1004), anti‐H4K5ac (PTM‐119), anti‐H4K8ac (PTM‐120), anti‐H4K16ac (PTM‐122) antibodies (PTM BioLabs, Hangzhou, China), and anti‐H4K12ac (Millipore, MA, USA, 04‐119). ImageJ software was used for quantification of the band intensities from the immunoblots.

### Protein purification and deacetylation assay

Uv1809^∆SP^‐His, Uv1809^23–245^‐His, Uv1809^246–391^‐His, and OsSRT2‐GST fusion proteins were produced in *E*. *coil* BL21 (DE3) cells by induction with 0.1 m isopropyl β‐D‐1‐thiogalactopyranoside (IPTG). The cells were then harvested by centrifugation and subjected to sonication. Recombinant Uv1809‐His and OsSRT2‐GST proteins were purified with HisPur™ Ni‐NTA Resin and GSTrap™ Resin (Thermo Scientific), respectively. For *in vitro* deacetylation assays, histone‐enriched proteins from rice spikelets were incubated with recombinant OsSRT2‐GST, GST, Uv1809^∆SP^‐His, Uv1809^23–245^‐His, or Uv1809^246–391^‐His in a reaction buffer (1 mm DTT, 2.7 mm KCl, 137 mm NaCl, 1 mm MgCl_2_ and 50 mm Tris–HCl pH, 8.5). The mixture was incubated at 30 °C for 2 h. The reaction products were then analysed by immunoblotting with anti‐H4K5ac and anti‐H4K8ac antibodies. *In vitro* deacetylase activity of recombinant OsSRT2‐GST was confirmed by fluorometric assays. An HDAC Assay Kit (Fluorescent) (Active Motif, Wuhan, China) was used to assess the HDAC activity of recombinant GST, Uv1809^∆SP^‐His, Uv1809^23–245^‐His, Uv1809^246–391^‐His, OsSRT2‐GST, OsSRT2‐GST and Uv1809^∆SP^‐His, OsSRT2‐GST and Uv1809^23–245^‐His, OsSRT2‐GST and Uv1809^246–391^‐His, and OsSRT2‐GST and the HADC inhibitor trichostatin A. Fluorescence was measured on a fluorescence plate reader with excitation and emission wavelengths of 355 and 460 nm, respectively. For *in vivo* deacetylation assays, pCNF‐*OsSRT2*, pCNG, pCNG‐*Uv1809*, pCNG‐*Uv1809* and pCNF‐*OsSRT2*, pCNG and pCNF‐*OsSRT2* vectors were infiltrated/co‐infiltrated into *N*. *benthamiana* leaves, the total protein was extracted at 2 dpi and then analysed by immunoblotting with anti‐H4K5ac and anti‐H4K8ac antibodies. Accordingly, the nuclear protein was extracted with Plant Cell Nuclear Extraction Kit (BestBio, BB‐3611223, Shanghai, China) from these *N*. *benthamiana* leaves, and the deacetylase activity of nuclear protein was confirmed by the HDAC Assay Kit (Fluorescent).

### ChIP‐seq and data analysis

Chromatin immunoprecipitation (ChIP) was performed as described previously (Lu *et al*., 2018). Briefly, 5 g of ZH11 or *ossrt2* mutant rice spikelets was crosslinked in 1% (v/v) formaldehyde. Chromatin was extracted and fragmented to 100‐ to 500‐bp fragments by sonication, and ChIP was performed using the anti‐H4K5ac and anti‐H4K8ac antibodies (Abcam, ab51997 and ab45166). Sequencing libraries were constructed with DNA from ChIP following the Illumina TruSeqCHIP Sample Prep Set A protocol and sequenced on an Illumina HiSeq2000 as 150‐bp paired‐end reads by Wuhan IGENEBOOK Biotechnology Co., Ltd.

FastP (v0.232) was used with the default parameters to trim low‐quality bases and adapters. Bowtie2 (version 2.3.5.1) was used to map cleaned reads to the rice genome (MSU7.0) using the default settings (Langmead and Salzberg, 2012). Samtools (v1.9) was then used to remove duplicate reads (Li *et al*., 2009). The following parameters were used in MACS (Zhang *et al*., 2008) to identify histone modification peaks: ‐f BAMPE ‐B ‐q 0.05 ‐g 3.6e+8. DiffBind (v3.5) (Stark and Brown, 2011) was used to identify histone modification sites that differed between the wild‐type and mutant rice plants. Peaks were annotated by Homer (v4.11) using the default parameters (Heinz *et al*., 2010). Metaplots were generated by ngsplot (v2.61) (Shen *et al*., 2014). Chromosome plots, correlation plots, and scatter plots were generated in R (v3.5). GSEA and GO pathways were analysed by clusterProfiler (v4.0) (Wu *et al*., 2021a,b).

### RT‐qPCR and ChIP‐qPCR

Total RNA was extracted using TRIzol reagent (Vazyme Biotech, Nanjing, China). First‐strand cDNA synthesis was carried out using cDNA Synthesis SuperMix (TransGen Biotech, China). RT‐qPCR was performed with TransStart® Tip Green qPCR SuperMix (TransGen Biotech, Beijing, China). The precipitated and input DNA was analysed by ChIP‐qPCR. Transcript levels were normalized using the rice ubiquitin gene (*OsUBQ1*) or *U*. *virens* β‐tubulin gene (*Uv8b_900*). At least three biological replicates were tested per treatment.

### Detection of ROS accumulation

Rice plants were grown on MS medium in the growth chamber for 12 days. Leaves of the seedlings were cut into discs (3 mm) and then submerged in distilled water in a 96‐well plate under light overnight. The distilled water was pipetted out and replaced with 100 μL of mixed solution for each well (the solution contained 50 μΜ luminol, 10 μg/mL horseradish peroxidase and 8 nm chitin) using a multiple‐channel pipette. Chemiluminescence was measured at 30 s intervals over a period of 20 min in a SPARK‐10M microplate reader (TECAN, Männedorf, Switzerland). Ten biological replicates were used for each sample. Distilled water was used as the mock control.

### Statistical analyses

Statistical analyses of each treatment were performed using SPSS version 14.0 software (SPSS, Chicago, IL), and when significant treatment effects (*P* ≤ 0.05) were found, the means were compared by the least significant difference (LSD) test at *P* = 0.05.

## Conflict of interest

The authors declare no conflict of interest.

## Author contributions

Y.M.P. conceived the project; X.Y.C. performed most of the experiments; Q.T.X. performed data analyses and provided technical support; Z.Y.W., L.Z., Y.H.W., J.B.H., and X.L.C. gave critical suggestions for the project; X.Y.C. and Q.T.X. wrote and revised the manuscript. All authors have read and approved the final manuscript.

## Supporting information


**Figure S1** Characterization of Uv1809 in *U*. *virens*.
**Figure S2** Gene deletion and complementation of *Uv1809* in *U*. *virens*.
**Figure S3** Deletion of *Uv1809* affects conidiation of *U*. *virens*.
**Figure S4** Subcellular localization of Uv1809 variants in *N*. *benthamiana* leaves.
**Figure S5** Uv1809 expressed in *M*. *oryzae* is translocated into rice cells during host infection.
**Figure S6** Expression and Agronomic traits of *5S‐Uv1809* and *35S‐EV* transgenic rice plants.
**Figure S7** HIGS of *Uv1809* in rice.
**Figure S8** Uv1809^246–391^ interact with OsSRT2.
**Figure S9** Expression and subcellular localization of OsSRT2.
**Figure S10** Mutation identified within and around the target sites of *OsSRT2* generated through CRISPR/Cas9‐mediated genome editing in rice.
**Figure S11** Agronomic traits of the wild‐type ZH11 and *ossrt2* mutants rice lines at mature stage following growth in field conditions.
**Figure S12** Multiscatter plots of RNA‐seq and ChIP‐seq.
**Figure S13** RT‐qPCR to validate differentially expressed genes from RNA‐seq.
**Figure S14** Pearson correlation coefficients of H4K5ac (a) and H4K8ac (b) ChIP‐seq replicates.
**Figure S15** Analysis of leaf H4K5 and H4K8 acetylation ChIP‐seq data of wild‐type spikelets.
**Figure S16** ROS level in ZH11 and *ossrt2* rice plants after chitin treatment.

**Table S1** Putative Uv1809‐interacting proteins identified by Y2H.
**Table S2** Transcriptionally up and downregulated genes in *ossrt2* mutant vs WT.
**Table S3** H4K5ac up and downregulated genes in *ossrt2* mutant vs WT.
**Table S4** H4K8ac up and downregulated genes in *ossrt2* mutant vs WT.
**Table S5** GO pathways of H4K5ac and H4K8ac upregulated genes in *ossrt2* mutant vs WT.
**Table S6** H4K5ac, H4K8ac and transcriptionally upregulated genes in *ossrt2* mutant vs WT.
**Table S7** GO pathways of H4K5ac, H4K8ac and transcriptionally upregulated genes in *ossrt2* mutant vs WT.

## Data Availability

RNA‐seq and ChIP‐seq data were deposited in the SRA database under accession number PRJNA912072.

## References

[pbi14174-bib-0001] Alvarez, M.E. , Florencia, N. and Damián, A.C. (2010) Epigenetic control of plant immunity. Mol. Plant Pathol. 11, 563–576. 20618712 10.1111/j.1364-3703.2010.00621.x PMC6640280

[pbi14174-bib-0002] Azevedo, C. , Sadanandom, A. , Kitagawa, K. , Freialdenhoven, A. , Shirasu, K. and Schulze‐Lefert, P. (2002) The RAR1 interactor SGT1, an essential component of R gene‐triggered disease resistance. Science, 295, 2073–2076. 11847307 10.1126/science.1067554

[pbi14174-bib-0003] Berger, S.L. (2007) The complex language of chromatin regulation during transcription. Nature, 447, 407–412. 17522673 10.1038/nature05915

[pbi14174-bib-0004] Chen, X. , Ding, A.B. and Zhong, X. (2020a) Functions and mechanisms of plant histone deacetylases. Sci. China Life Sci. 63, 206–216. 31879846 10.1007/s11427-019-1587-x

[pbi14174-bib-0005] Chen, X.Y. , Tang, J.T. , Pei, Z.X. , Liu, H. , Huang, J.B. , Luo, C.X. , Hsiang, T. *et al*. (2020b) The “pears and lemons” protein UvPal1 regulates development and virulence of *Ustilaginoidea virens* . Environ. Microbiol. 22, 5414–5432. 33073491 10.1111/1462-2920.15284

[pbi14174-bib-0006] Chen, X.Y. , Xu, Q.T. , Duan, Y.H. , Liu, H. , Chen, X.L. , Huang, J.B. , Luo, C.X. *et al*. (2021) *Ustilaginoidea virens* modulates lysine 2‐hydroxyisobutyrylation in rice flowers during infection. J. Integr. Plant Biol. 63, 1801–1814. 34245484 10.1111/jipb.13149

[pbi14174-bib-0007] Chen, X. , Duan, Y. , Qiao, F. , Liu, H. , Huang, J. , Luo, C. , Chen, X. *et al*. (2022a) A secreted fungal effector suppresses rice immunity through host histone hypoacetylation. New Phytol. 235, 1977–1994. 35592995 10.1111/nph.18265

[pbi14174-bib-0008] Chen, X. , Li, X. , Duan, Y. , Pei, Z. , Liu, H. , Yin, W. , Huang, J. *et al*. (2022b) A secreted fungal subtilase interferes with rice immunity via degradation of SUPPRESSOR OF G2 ALLELE OF *skp1* . Plant Physiol. 190, 1474–1489. 35861434 10.1093/plphys/kiac334 PMC9516721

[pbi14174-bib-0009] Chen, X. , Pei, Z. , Liu, H. , Huang, J. , Chen, X. , Luo, C. , Hsiang, T. *et al*. (2022c) Host‐induced gene silencing of fungal‐specific genes of *Ustilaginoidea virens* confers effective resistance to rice false smut. Plant Biotechnol. J. 20, 253–255. 34850522 10.1111/pbi.13756 PMC8753348

[pbi14174-bib-0010] Cheng, W. , Song, S.X. , Li, H.P. , Cao, L.H. , Sun, K. , Qiu, X.L. , Xu, Y.B. *et al*. (2015) Host‐induced gene silencing of an essential chitin synthase gene confers durable resistance to *Fusarium* head blight and seedling blight in wheat. Plant Biotechnol. J. 13, 1335–1345. 25735638 10.1111/pbi.12352

[pbi14174-bib-0011] Choi, S.M. , Song, H.R. , Han, S.K. , Han, M. , Kim, C.Y. , Park, J. , Lee, Y.H. *et al*. (2012) HDA19 is required for the repression of salicylic acid biosynthesis and salicylic acid‐mediated defense responses in Arabidopsis. Plant J. 71, 135–146. 22381007 10.1111/j.1365-313X.2012.04977.x

[pbi14174-bib-0012] Couto, D. and Zipfel, C. (2016) Regulation of pattern recognition receptor signaling in plants. Nat. Rev. Immunol. 16, 537–552. 27477127 10.1038/nri.2016.77

[pbi14174-bib-0013] Dangl, J.L. , Horvath, D.M. and Staskawicz, B.J. (2013) Pivoting the plant immune system from dissection to deployment. Science, 341, 746–751. 23950531 10.1126/science.1236011 PMC3869199

[pbi14174-bib-0014] Darino, M. , Chia, K.S. , Marques, J. , Aleksza, D. , Soto‐Jiménez, L.M. , Saado, I. , Uhse, S. *et al*. (2021) *Ustilago maydis* effector Jsi1 interacts with Topless corepressor, hijacking plant jasmonate/ethylene signaling. New Phytol. 229, 3393–3407. 33247447 10.1111/nph.17116 PMC8126959

[pbi14174-bib-0015] DeFraia, C.T. , Wang, Y.S. , Yao, J.Q. and Mou, Z.L. (2013) Elongator subunit 3 positively regulates plant immunity through its histone acetyltransferase and radical S‐adenosylmethionine domains. BMC Plant Biol. 13, 1–13. 23856002 10.1186/1471-2229-13-102 PMC3728140

[pbi14174-bib-0016] Ding, B. , Bellizzi, M.R. , Ning, Y.S. , Meyers, B.C. and Wang, G.L. (2012) HDT701, a histone H4 deacetylase, negatively regulates plant innate immunity by modulating histone H4 acetylation of defense‐related genes in rice. Plant Cell, 24, 3783–3794. 22968716 10.1105/tpc.112.101972 PMC3480302

[pbi14174-bib-0017] Dodds, P.N. and Rathjen, J.P. (2010) Plant immunity: towards an integrated view of plant‐pathogen interactions. Nat. Rev. Genet. 11, 539–548. 20585331 10.1038/nrg2812

[pbi14174-bib-0018] Dou, D.L. and Zhou, J.M. (2012) Phytopathogen effectors subverting host immunity: different foes, similar battleground. Cell Host Microbe, 12, 484–495. 23084917 10.1016/j.chom.2012.09.003

[pbi14174-bib-0019] Du, Y. , Chen, X. , Guo, Y. , Zhang, X. , Zhang, H. , Li, F. , Huang, G. *et al*. (2021) *Phytophthora infestans* RXLR effector PITG20303 targets a potato MKK1 protein to suppress plant immunity. New Phytol. 229, 501–515. 32772378 10.1111/nph.16861

[pbi14174-bib-0020] Eulgem, T. and Somssich, I.E. (2007) Networks of WRKY transcription factors in defense signaling. Curr. Opin. Plant Biol. 10, 366–371. 17644023 10.1016/j.pbi.2007.04.020

[pbi14174-bib-0021] Fan, J. , Liu, J. , Gong, Z.Y. , Xu, P.Z. , Hu, X.H. , Wu, J.L. , Li, G.B. *et al*. (2020) The false smut pathogen *Ustilaginoidea virens* requires rice stamens for false smut ball formation. Environ. Microbiol. 2, 646–659. 10.1111/1462-2920.14881 PMC7028044 31797523

[pbi14174-bib-0022] Fukada, F. , Rössel, N. , Münch, K. , Glatter, T. and Kahmann, R. (2021) A small *Ustilago maydis* effector acts as a novel adhesin for hyphal aggregation in plant tumors. New Phytol. 231, 416–431. 33843063 10.1111/nph.17389

[pbi14174-bib-0023] Gómez‐Díaz, E. , Jordà, M. , Peinado, M.A. and Rivero, A. (2012) Epigenetics of host‐pathogen interactions: the road ahead and the road behind. PLoS Pathog. 8, e1003007. 23209403 10.1371/journal.ppat.1003007 PMC3510240

[pbi14174-bib-0024] Guo, X.Y. , Li, Y. , Fan, J. , Xiong, H. , Xu, F.X. , Shi, J. , Shi, Y. *et al*. (2019) Host‐induced gene silencing of *MoAP1* confers broad‐spectrum resistance to *Magnaporthe oryzae* . Front. Plant Sci. 10, 433. 31024598 10.3389/fpls.2019.00433 PMC6465682

[pbi14174-bib-0025] Heinz, S. , Benner, C. , Spann, N. , Bertolino, E. , Lin, Y.C. , Laslo, P. , Cheng, J.X. *et al*. (2010) Simple combinations of lineage‐determining transcription factors prime cis‐regulatory elements required for macrophage and B cell identities. Mol. Cell 38, 576–589. 20513432 10.1016/j.molcel.2010.05.004 PMC2898526

[pbi14174-bib-0026] Hu, Z. , Dang, Y. , Liu, C.S. , Zhou, L.G. and Liu, H. (2019) Acute exposure to ustiloxin A affects growth and development of early life zebrafish, *Danio rerio* . Chemosphere, 226, 851–857. 30978596 10.1016/j.chemosphere.2019.04.002

[pbi14174-bib-0027] Hussain, R.M.F. , Sheikh, A.H. , Haider, I. , Quareshy, M. and Linthorst, H.J.M. (2018) Arabidopsis WRKY50 and TGA transcription factors synergistically activate expression of PR1. Front. Plant Sci. 9, 930. 30057584 10.3389/fpls.2018.00930 PMC6053526

[pbi14174-bib-0028] Jacobs, K.A. , Collins‐Racie, L.A. , Colbert, M. , Duckett, M.K. , Golden‐Fleet, M. , Kelleher, K. , Kriz, R. *et al*. (1997) A genetic selection for isolating cDNAs encoding secreted proteins. Gene, 198, 289–296. 9370294 10.1016/s0378-1119(97)00330-2

[pbi14174-bib-0029] Jiang, C. , Hei, R.N. , Yang, Y. , Zhang, S. , Wang, Q. , Wang, W. , Zhang, Q. *et al*. (2020) An orphan protein of *Fusarium graminearum* modulates host immunity by mediating proteasomal degradation of TaSnRK1α. Nat. Commun. 11, 4382. 32873802 10.1038/s41467-020-18240-y PMC7462860

[pbi14174-bib-0030] Jin, H. , Choi, S.M. , Kang, M.J. , Yun, S.H. , Kwon, D.J. , Noh, Y.S. and Noh, B. (2018) Salicylic acid‐induced transcriptional reprogramming by the HAC‐NPR1‐TGA histone acetyltransferase complex in Arabidopsis. Nucleic Acids Res. 46, 11712–11725. 30239885 10.1093/nar/gky847 PMC6294559

[pbi14174-bib-0031] Jones, J.D. and Dangl, J.L. (2006) The plant immune system. Nature, 444, 323–329. 17108957 10.1038/nature05286

[pbi14174-bib-0032] Kim, K.C. , Lai, Z. , Fan, B. and Chen, Z. (2008) *Arabidopsis* WRKY38 and WRKY62 transcription factors interact with histone deacetylase 19 in basal defense. Plant Cell, 20, 2357–2371. 18776063 10.1105/tpc.107.055566 PMC2570728

[pbi14174-bib-0033] Koch, A. , Kumar, N. , Weber, L. , Keller, H. , Imani, J. and Kogel, K.H. (2013) Host‐induced gene silencing of cytochrome P450 lanosterol C14α‐demethylase‐encoding genes confers strong resistance to *Fusarium* species. Proc. Natl. Acad. Sci. USA, 110, 19324–19329. 24218613 10.1073/pnas.1306373110 PMC3845197

[pbi14174-bib-0034] Kong, L. , Qiu, X.F. , Kang, J.G. *et al*. (2017) A *Phytophthora* effector manipulates host histone acetylation and reprograms defense gene expression to promote infection. Curr. Biol. 27, 981–991. 28318979 10.1016/j.cub.2017.02.044

[pbi14174-bib-0035] Langmead, B. and Salzberg, S.L. (2012) Fast gapped‐read alignment with Bowtie 2. Nat. Methods, 9, 357–359. 22388286 10.1038/nmeth.1923 PMC3322381

[pbi14174-bib-0036] Li, H. , Handsaker, B. , Wysoker, A. , Fennell, T. , Ruan, J. , Homer, N. , Marth, G. *et al*. (2009) The sequence alignment/map format and SAMtools. Bioinformatics, 25, 2078–2079. 19505943 10.1093/bioinformatics/btp352 PMC2723002

[pbi14174-bib-0037] Li, H.Y. , Wang, H.N. , Jing, M.F. , Zhu, J. , Guo, B. , Wang, Y. , Lin, Y. *et al*. (2018) A *Phytophthora* effector recruits a host cytoplasmic transacetylase into nuclear speckles to enhance plant susceptibility. Elife, 7, e40039. 30346270 10.7554/eLife.40039 PMC6249003

[pbi14174-bib-0038] Li, W.T. , Wang, K. , Chern, M. , Liu, Y. , Zhu, Z. , Liu, J. , Zhu, X. *et al*. (2020) Sclerenchyma cell thickening through enhanced lignification induced by OsMYB30 prevents fungal penetration of rice leaves. New Phytol. 226, 1850–1863. 32112568 10.1111/nph.16505

[pbi14174-bib-0039] Li, G.B. , Fan, J. , Wu, J.L. , He, J.X. , Liu, J. , Shen, S. , Gishkori, Z.G.N. *et al*. (2021a) The flower‐infecting fungus *Ustilaginoidea virens* subverts plant immunity by secreting a Chitin‐binding protein. Front. Plant Sci. 12, 733245. 34421978 10.3389/fpls.2021.733245 PMC8377610

[pbi14174-bib-0040] Li, Q. , Wang, B. , Yu, J.P. and Dou, D.L. (2021b) Pathogen‐informed breeding for crop disease resistance. J. Integr. Plant Biol. 63, 305–311. 33095498 10.1111/jipb.13029

[pbi14174-bib-0041] Lin, H. , Wang, M. , Chen, Y. , Nomura, K. , Hui, S. , Gui, J. , Zhang, X. *et al*. (2022) An MKP‐MAPK protein phosphorylation cascade controls vascular immunity in plants. Sci. Adv. 8, eabg8723. 35263144 10.1126/sciadv.abg8723 PMC8906744

[pbi14174-bib-0042] Lu, Y. , Xu, Q.T. , Liu, Y. , Yu, Y. , Cheng, Z.Y. , Zhao, Y. and Zhou, D.X. (2018) Dynamics and functional interplay of histone lysine butyrylation, crotonylation, and acetylation in rice under starvation and submergence. Genome Biol. 19, 144. 30253806 10.1186/s13059-018-1533-y PMC6154804

[pbi14174-bib-0043] Machado, A.K. , Brown, N.A. , Urban, M. , Kanyuka, K. and Hammond‐Kosack, K.E. (2018) RNAi as an emerging approach to control *Fusarium* head blight disease and mycotoxin contamination in cereals. Pest Manag. Sci. 74, 790–799. 28967180 10.1002/ps.4748 PMC5873435

[pbi14174-bib-0044] Mitsuhara, I. , Iwai, T. , Seo, S. , Yanagawa, Y. , Kawahigasi, H. , Hirose, S. , Ohkawa, Y. *et al*. (2008) Characteristic expression of twelve rice PR1 family genes in response to pathogen infection, wounding, and defense‐related signal compounds (121/180). Mol. Genet. Genomics, 279, 415–427. 18247056 10.1007/s00438-008-0322-9 PMC2270915

[pbi14174-bib-0045] Nguyen, B.A.N. , Pogoutse, A. , Provart, N. and Moses, A.M. (2009) NLStradamus: a simple hidden markov model for nuclear localization signal prediction. BMC Bioinformatics, 10, 202. 19563654 10.1186/1471-2105-10-202 PMC2711084

[pbi14174-bib-0046] Nowara, D. , Gay, A. , Lacomme, C. , Shaw, J. , Ridout, C. , Douchkov, D. , Hensel, G. *et al*. (2010) HIGS: host‐induced gene silencing in the obligate biotrophic fungal pathogen *Blumeria graminis* . Plant Cell, 22, 3130–3141. 20884801 10.1105/tpc.110.077040 PMC2965548

[pbi14174-bib-0047] Park, C.J. , Caddell, D.F. and Ronald, P.C. (2012) Protein phosphorylation in plant immunity: insights into the regulation of pattern recognition receptor‐mediated signaling. Front. Plant Sci. 3, 177. 22876255 10.3389/fpls.2012.00177 PMC3411088

[pbi14174-bib-0048] Parker, A.H. , Wilkinson, S.W. and Ton, J. (2022) Epigenetics: a catalyst of plant immunity against pathogens. New Phytol. 233, 66–83. 34455592 10.1111/nph.17699

[pbi14174-bib-0049] Qi, T. , Zhu, X.G. , Tan, C.L. , Liu, P. , Guo, J. , Kang, Z.S. and Guo, J. (2018) Host‐induced gene silencing of an important pathogenicity factor PsCPK1 in *Puccinia striiformis* f. sp. *tritici* enhances resistance of wheat to stripe rust. Plant Biotechnol. J. 16, 797–807. 28881438 10.1111/pbi.12829 PMC5814584

[pbi14174-bib-0051] Qiu, S. , Fang, A. , Zheng, X. , Wang, S. , Wang, J. , Fan, J. , Sun, Z. *et al*. (2022) *Ustilaginoidea virens* nuclear effector SCRE4 suppresses rice immunity via inhibiting expression of a positive immune regulator OsARF17. Int. J. Mol. Sci. 23, 10527. 36142440 10.3390/ijms231810527 PMC9501289

[pbi14174-bib-0052] Shen, L. , Shao, N. , Liu, X. and Nestler, E. (2014) ngs.plot: Quick mining and visualization of next‐generation sequencing data by integrating genomic databases. BMC Genomics, 15, 284. 24735413 10.1186/1471-2164-15-284 PMC4028082

[pbi14174-bib-0053] Song, T.Q. , Zhang, Y. , Zhang, Q. , Zhang, X. , Shen, D. , Yu, J. , Yu, M. *et al*. (2021) The N‐terminus of an *Ustilaginoidea virens* Ser‐Thr‐rich glycosylphosphatidylinositol‐anchored protein elicits plant immunity as a MAMP. Nat. Commun. 12, 1–15. 33907187 10.1038/s41467-021-22660-9 PMC8079714

[pbi14174-bib-0054] Stark, R. and Brown, G. (2011) DiffBind differential binding analysis of ChIP‐Seq peak data. *R*. *package version*, **100**(4.3).

[pbi14174-bib-0055] Sun, W.X. , Fan, J. , Fang, A.F. , Li, Y.J. , Tariqjaveed, M. , Li, D.Y. , Hu, D.W. *et al*. (2020) *Ustilaginoidea virens*: insights into an emerging rice pathogen. Annu. Rev. Phytopathol. 58, 363–385. 32364825 10.1146/annurev-phyto-010820-012908

[pbi14174-bib-0056] Tang, J.T. , Chen, X.Y. , Yan, Y.Q. , Huang, J.B. , Luo, C.X. , Hsiang, T. and Zheng, L. (2021) Comprehensive transcriptome profiling reveals abundant long non‐coding RNAs associated with development of the rice false smut fungus, *Ustilaginoidea virens* . Environ. Microbiol. 23, 4998–5013. 33587785 10.1111/1462-2920.15432

[pbi14174-bib-0057] Tang, W.S. , Zhong, L. , Ding, Q.Q. , Dou, Y.N. , Li, W.W. , Xu, Z.S. , Zhou, Y.B. *et al*. (2022) Histone deacetylase AtSRT2 regulates salt tolerance during seed germination via repression of vesicle‐associated membrane protein 714 (VAMP714) in Arabidopsis. New Phytol. 234, 1278–1293. 35224735 10.1111/nph.18060

[pbi14174-bib-0058] Tariqjaveed, M. , Mateen, A. , Wang, S. , Qiu, S. , Zheng, X. , Zhang, J. , Bhadauria, V. *et al*. (2021) Versatile effectors of phytopathogenic fungi target host immunity. J. Integr. Plant Biol. 63, 1856–1873. 34383388 10.1111/jipb.13162

[pbi14174-bib-0059] Wang, H.H. , Hao, J.J. , Chen, X.J. , Hao, Z. , Wang, X. , Lou, Y. , Peng, Y. *et al*. (2007) Overexpression of rice WRKY89 enhances ultraviolet B tolerance and disease resistance in rice plant. Plant Mol. Biol. 65, 799–815. 17960484 10.1007/s11103-007-9244-x

[pbi14174-bib-0060] Wang, C. , Gao, F. , Wu, J. , Dai, J. , Wei, C. and Li, Y. (2010) Arabidopsis putative deacetylase AtSRT2 regulates basal defense by suppressing PAD4, EDS5 and SID2 expression. Plant Cell Physiol. 51, 1291–1299. 20573705 10.1093/pcp/pcq087 PMC2920754

[pbi14174-bib-0061] Wang, Y. , Hu, Q. , Wu, Z. , Wang, H. , Han, S. , Jin, Y. , Zhou, J. *et al*. (2017) HISTONE DEACETYLASE 6 represses pathogen defence responses in *Arabidopsis thaliana* . Plant Cell Environ. 40, 2972–2986. 28770584 10.1111/pce.13047

[pbi14174-bib-0062] Wang, W. , Feng, B. , Zhou, J.M. and Tang, D.Z. (2020a) Plant immune signaling: advancing on two frontiers. J. Integr. Plant Biol. 62, 2–24. 31846204 10.1111/jipb.12898

[pbi14174-bib-0063] Wang, M.H. , Wu, L. , Mei, Y.Z. , Zhao, Y.F. , Ma, Z.H. , Zhang, X. and Chen, Y. (2020b) Host‐induced gene silencing of multiple genes of *Fusarium graminearum* enhances resistance to *Fusarium* head blight in wheat. Plant Biotechnol. J. 18, 2373–2375. 32436275 10.1111/pbi.13401 PMC7680546

[pbi14174-bib-0064] Wani, S.H. , Anand, S. , Singh, B. , Bohra, A. and Joshi, R. (2021) WRKY transcription factors and plant defense responses: latest discoveries and future prospects. Plant Cell Rep. 40, 1071–1085. 33860345 10.1007/s00299-021-02691-8

[pbi14174-bib-0065] Wu, Z. , He, L. , Jin, Y. , Chen, J. , Shi, H. , Wang, Y. and Yang, W. (2021a) HISTONE DEACETYLASE 6 suppresses salicylic acid biosynthesis to repress autoimmunity. Plant Physiol. 187, 2592–2607. 34618093 10.1093/plphys/kiab408 PMC8644357

[pbi14174-bib-0066] Wu, T. , Hu, E. , Xu, S. , Chen, M. , Guo, P. , Dai, Z. , Feng, T. *et al*. (2021b) clusterProfiler 4.0: A universal enrichment tool for interpreting omics data. Innovation (Camb), 2, 100141. 34557778 10.1016/j.xinn.2021.100141 PMC8454663

[pbi14174-bib-0555] Xu, Q. , Liu, Q. , Chen, Z. , Yue, Y. , Liu, Y. , Zhao, Y. and Zhou, D.X. (2021) Histone deacetylases control lysine acetylation of ribosomal proteins in rice. Nucleic Acids Res. 49, 4613–4628. 33836077 10.1093/nar/gkab244 PMC8096213

[pbi14174-bib-0067] Yang, B. , Yang, S. , Guo, B. , Wang, Y. , Zheng, W. , Tian, M. , Dai, K. *et al*. (2021) The *Phytophthora* effector Avh241 interacts with host NDR1‐like proteins to manipulate plant immunity. J. Integr. Plant Biol. 63, 1382–1396. 33586843 10.1111/jipb.13082

[pbi14174-bib-0068] Yang, J. , Zhang, N. , Wang, J. , Fang, A. , Fan, J. , Li, D. , Li, Y. *et al*. (2022) SnRK1A‐mediated phosphorylation of a cytosolic ATPase positively regulates rice innate immunity and is inhibited by *Ustilaginoidea virens* effector SCRE1. New Phytol. 236, 1422–1440. 36068953 10.1111/nph.18460

[pbi14174-bib-0069] Yoshiaki, Y. , Goto, K. , Takai, R. , Iwano, M. , Takayama, S. , Isogai, A. and Che, F.‐S. (2005) Function of the rice gp91phox homologs OsrbohA and OsrbohE genes in ROS‐dependent plant immune responses. Plant Biotechnol. 22, 127–135.

[pbi14174-bib-0070] Zhang, Y. , Liu, T. , Meyer, C.A. , Eeckhoute, J. , Johnson, D.S. , Bernstein, B.E. , Nusbaum, C. *et al*. (2008) Model‐based analysis of ChIP‐Seq (MACS). Genome Biol. 9, R137. 18798982 10.1186/gb-2008-9-9-r137 PMC2592715

[pbi14174-bib-0071] Zhang, Y. , Zhang, K. , Fang, A.F. , Han, Y. , Yang, J. , Xue, M. , Bao, J. *et al*. (2014) Specific adaptation of *Ustilaginoidea virens* in occupying host florets revealed by comparative and functional genomics. Nat. Commun. 5, 3849. 24846013 10.1038/ncomms4849

[pbi14174-bib-0072] Zhang, H. , Zhao, Y. and Zhou, D.X. (2017) Rice NAD+‐dependent histone deacetylase OsSRT1 represses glycolysis and regulates the moonlighting function of GAPDH as a transcriptional activator of glycolytic genes. Nucleic Acids Res. 45, 12241–12255. 28981755 10.1093/nar/gkx825 PMC5716216

[pbi14174-bib-0073] Zhang, F. , Wang, L. , Ko, E.E. , Shao, K. and Qiao, H. (2018) Histone deacetylases SRT1 and SRT2 interact with ENAP1 to mediate ethylene‐induced transcriptional repression. Plant Cell, 30, 153–166. 29298835 10.1105/tpc.17.00671 PMC5810571

[pbi14174-bib-0074] Zhang, N. , Yang, J. , Fang, A.F. , Wang, J. , Li, D. , Li, Y. , Wang, S. *et al*. (2020) The essential effector SCRE1 in *Ustilaginoidea virens* suppresses rice immunity via a small peptide region. Mol. Plant Pathol. 21, 445–459. 32087618 10.1111/mpp.12894 PMC7060142

[pbi14174-bib-0075] Zhang, K. , Zhao, Z. , Zhang, Z. , Li, Y. , Li, S. , Yao, N. , Hsiang, T. *et al*. (2021) Insights into genomic evolution from the chromosomal and mitochondrial genomes of *Ustilaginoidea virens* . Phytopathol. Res. 3, 9.

[pbi14174-bib-0076] Zheng, X. , Fang, A. , Qiu, S. , Zhao, G. , Wang, J. , Wang, S. , Wei, J. *et al*. (2022) *Ustilaginoidea virens* secretes a family of phosphatases that stabilize the negative immune regulator OsMPK6 and suppress plant immunity. Plant Cell, 34, 3088–3109. 35639755 10.1093/plcell/koac154 PMC9338817

[pbi14174-bib-0077] Zhou, C.H. , Zhang, L. , Duan, J. , Miki, B. and Wu, K.Q. (2005) Histone deacetylase 19 is involved in jasmonic acid and ethylene signaling of pathogen response in Arabidopsis. Plant Cell, 17, 1196–1204. 15749761 10.1105/tpc.104.028514 PMC1087996

[pbi14174-bib-0078] Zhu, X. , Qi, T. , Yang, Q. , He, F. , Tan, C. , Ma, W. , Voegele, R.T. *et al*. (2017) Host‐induced gene silencing of the MAPKK gene *PsFUZ7* confers stable resistance to wheat stripe rust. Plant Physiol. 175, 1853–1863. 29070517 10.1104/pp.17.01223 PMC5717739

