# Supplementary material for: Ustilaginoidea virens‐secreted effector Uv1809 suppresses rice immunity by enhancing OsSRT2‐mediated histone deacetylation
Source: Plant Biotechnol J. 2023 Sep 16;22(1):148–64. doi: 10.1111/pbi.14174 (PMC10754013; doi:10.1111/pbi.14174)
Supplement: Supplementary file 2 — Table S1 Putative Uv1809‐interacting proteins identified by Y2H. Table S2 Transcriptionally up and downregulated genes in ossrt2 mutant vs WT. Table S3 H4K5ac up and downregulated genes in ossrt2 mutant vs WT. Table S4 H4K8ac up and downregulated genes in ossrt2 mutant vs WT. Table S5 GO pathways of H4K5ac and H4K8ac upregulated genes in ossrt2 mutant vs WT. Table S6 H4K5ac, H4K8ac and transcriptionally upregulated genes in ossrt2 mutant vs WT. Table S7 GO pathways of H4K5ac, H4K8ac and transcriptionally upregulated genes in ossrt2 mutant vs WT. [file PBI-22-148-s002.docx]

**Table S1.** Putative Uv1809-interacting proteins identified by Y2H.

| **Gene ID** | **Annotation** |
| --- | --- |
| Os02g0714300 | AT-rich interactive domain-containing protein 5 |
| Os08g0177300 | Chromatin modification-related protein EAF1 B |
| Os06g0255200 | Chromatin structure-remodeling complex protein SYD |
| Os02g0324300 | Coiled-coil domain-containing protein 130 |
| Os03g0116500 | COP9 signalosome complex subunit 1 |
| Os11g0524400 | Disease resistance protein RGA5-like |
| Os11g0296500 | DNA-binding protein HEXBP |
| Os06g0608800 | E3 ubiquitin-protein ligase RGLG2 |
| Os12g0517100 | F-box/LRR-repeat protein 14 |
| Os01g0711400 | Glycine dehydrogenase |
| Os01g0218800 | Histone-lysine N-methyltransferase ATX5 |
| Os11g0602200 | Histone-lysine N-methyltransferase, H3 lysine-9 specific SUVH1 |
| Os05g0132100 | Long chain acyl-CoA synthetase 1 |
| Os03g0430100 | Lysine-specific demethylase JMJ25 |
| Os02g0192300 | Methyl-CpG-binding domain-containing protein 9 |
| Os04g0437600 | Mitogen-activated protein kinase kinase kinase 3 |
| Os12g0179800 | NAD-dependent protein deacylase SRT2 |
| Os05g0256100 | Probable LRR receptor-like serine/threonine-protein kinase |
| Os05g0530500 | Serine/threonine protein kinase OSK1 |
| Os03g0289100 | Serine/threonine protein kinase OSK3 |
| Os12g0179800 | Histone deacetylase SRT702 |
| Os08g0484600 | Serine/threonine protein kinase OSK4 |
| Os05g0596600 | Structural maintenance of chromosomes protein 5 |
| Os06g0682800 | Zinc finger CCCH domain-containing protein 13-like |

**Table S2.** Transcriptionally up and downregulated genes in *ossrt2* mutant vs WT.

| Gene ID | Log_2_FoldChange | *P* value | Expression |
| --- | --- | --- | --- |
| LOC_Os03g02470 | 12.62504617 | 1.84E-26 | up |
| LOC_Os05g45160 | 12.28694644 | 4.30E-25 | up |
| LOC_Os03g18779 | 11.72619388 | 6.15E-23 | up |
| LOC_Os05g50390 | 10.50865971 | 2.02E-18 | up |
| LOC_Os04g34500 | 10.29822023 | 9.60E-18 | up |
| LOC_Os12g07180 | 9.682169278 | 1.30E-15 | up |
| LOC_Os11g35300 | 9.548640753 | 3.87E-15 | up |
| LOC_Os03g43100 | 9.425878178 | 9.70E-15 | up |
| LOC_Os08g09990 | 8.790587131 | 1.04E-12 | up |
| LOC_Os02g53240 | 8.743774198 | 2.06E-39 | up |
| LOC_Os10g41350 | 8.41934038 | 2.12E-11 | up |
| LOC_Os10g24004 | 8.333166882 | 2.78E-22 | up |
| LOC_Os02g15350 | 8.157331111 | 1.86E-10 | up |
| LOC_Os06g13690 | 8.044960979 | 2.32E-10 | up |
| LOC_Os01g41960 | 7.911613786 | 7.13E-10 | up |
| LOC_Os11g10590 | 7.831821259 | 5.02E-05 | up |
| LOC_Os12g24050 | 7.74453305 | 3.65E-36 | up |
| LOC_Os06g21369 | 7.687545647 | 6.14E-13 | up |
| LOC_Os01g49750 | 7.619898094 | 4.62E-09 | up |
| LOC_Os03g58320 | 7.536271257 | 8.80E-17 | up |
| LOC_Os05g45170 | 7.369605249 | 4.43E-17 | up |
| LOC_Os09g10660 | 7.340439005 | 3.17E-08 | up |
| LOC_Os01g03680 | 7.0159552 | 4.69E-15 | up |
| LOC_Os12g14440 | 6.970316808 | 6.04E-10 | up |
| LOC_Os12g35000 | 6.822673652 | 8.73E-07 | up |
| LOC_Os12g39280 | 6.797820911 | 1.08E-06 | up |
| LOC_Os11g10250 | 6.643646107 | 3.52E-06 | up |
| LOC_Os10g35160 | 6.637766713 | 2.17E-06 | up |
| LOC_Os05g37810 | 6.620024505 | 3.36E-06 | up |
| LOC_Os03g27400 | 6.604866487 | 2.53E-06 | up |
| LOC_Os05g45130 | 6.442418765 | 6.70E-06 | up |
| LOC_Os04g52790 | 6.368729591 | 1.49E-05 | up |
| LOC_Os04g46200 | 6.314391889 | 5.24E-05 | up |
| LOC_Os04g33430 | 6.120752046 | 5.05E-05 | up |
| LOC_Os05g07170 | 6.107053356 | 5.41E-05 | up |
| LOC_Os08g07090 | 6.059134315 | 5.19E-05 | up |
| LOC_Os05g33140 | 6.046937683 | 0.000104748 | up |
| LOC_Os05g34490 | 6.041136254 | 9.08E-08 | up |
| LOC_Os12g12690 | 6.027667524 | 0.000378042 | up |
| LOC_Os08g07080 | 5.978059139 | 1.32E-33 | up |
| LOC_Os06g07420 | 5.955335379 | 0.000123546 | up |
| LOC_Os10g38660 | 5.947044657 | 9.17E-05 | up |
| LOC_Os12g02140 | 5.894894355 | 0.000143477 | up |
| LOC_Os06g20740 | 5.884940792 | 0.000224533 | up |
| LOC_Os04g32080 | 5.884302298 | 2.35E-07 | up |
| LOC_Os10g39890 | 5.861186442 | 0.000159503 | up |
| LOC_Os02g12470 | 5.861076535 | 0.000135191 | up |
| LOC_Os12g27220 | 5.809931315 | 0.000283526 | up |
| LOC_Os03g14820 | 5.749546197 | 7.20E-12 | up |
| LOC_Os06g19430 | 5.744660206 | 0.000349336 | up |
| LOC_Os07g18130 | 5.592883632 | 0.000546796 | up |
| LOC_Os07g34370 | 5.574578629 | 1.61E-05 | up |
| LOC_Os05g23050 | 5.564555674 | 0.000605092 | up |
| LOC_Os07g15970 | 5.38856406 | 0.001143666 | up |
| LOC_Os07g37385 | 5.370685617 | 3.49E-05 | up |
| LOC_Os03g36100 | 5.352995774 | 5.32E-25 | up |
| LOC_Os10g21140 | 5.345990223 | 0.002069494 | up |
| LOC_Os05g45110 | 5.317053571 | 0.002019913 | up |
| LOC_Os12g27254 | 5.247135535 | 0.002344376 | up |
| LOC_Os01g72470 | 5.2442988 | 0.001970428 | up |
| LOC_Os03g55220 | 5.182551697 | 0.004216786 | up |
| LOC_Os02g26794 | 5.145458602 | 0.001640481 | up |
| LOC_Os09g10650 | 5.091571374 | 7.51E-42 | up |
| LOC_Os03g64260 | 5.080590291 | 0.004556597 | up |
| LOC_Os02g21320 | 5.031467847 | 4.88E-07 | up |
| LOC_Os06g32600 | 5.031256582 | 5.77E-08 | up |
| LOC_Os02g02930 | 5.01243515 | 1.62E-13 | up |
| LOC_Os12g04300 | 4.969028381 | 0.007776011 | up |
| LOC_Os11g29780 | 4.913550857 | 0.009132868 | up |
| LOC_Os03g30800 | 4.87547344 | 0.008334809 | up |
| LOC_Os05g31100 | 4.872645739 | 0.017389879 | up |
| LOC_Os01g62740 | 4.846367583 | 0.010991559 | up |
| LOC_Os05g46790 | 4.838062429 | 0.000297626 | up |
| LOC_Os11g06770 | 4.831510041 | 0.000651286 | up |
| LOC_Os09g03200 | 4.819315423 | 0.020555685 | up |
| LOC_Os09g36850 | 4.799158346 | 0.013064074 | up |
| LOC_Os01g46830 | 4.792097685 | 0.021017804 | up |
| LOC_Os01g29450 | 4.764370663 | 0.010315455 | up |
| LOC_Os12g13800 | 4.760428409 | 0.011838798 | up |
| LOC_Os05g23650 | 4.756592987 | 0.011154796 | up |
| LOC_Os03g59146 | 4.748879614 | 0.011370297 | up |
| LOC_Os04g52440 | 4.739044954 | 2.51E-07 | up |
| LOC_Os12g02470 | 4.726897081 | 0.000419028 | up |
| LOC_Os06g40240 | 4.705503685 | 5.30E-68 | up |
| LOC_Os06g03150 | 4.67725044 | 0.017351682 | up |
| LOC_Os08g14950 | 4.648144528 | 0.018543364 | up |
| LOC_Os01g54515 | 4.639603334 | 0.017670231 | up |
| LOC_Os08g19694 | 4.635339031 | 0.019087817 | up |
| LOC_Os06g37340 | 4.630092659 | 0.03118108 | up |
| LOC_Os02g30700 | 4.599886723 | 4.30E-07 | up |
| LOC_Os09g38104 | 4.594337109 | 0.031962064 | up |
| LOC_Os08g07100 | 4.591204144 | 2.56E-06 | up |
| LOC_Os05g12040 | 4.582389852 | 2.35E-06 | up |
| LOC_Os01g42420 | 4.56250968 | 0.021360179 | up |
| LOC_Os05g19500 | 4.557841702 | 0.022939465 | up |
| LOC_Os08g29510 | 4.557841702 | 0.022939465 | up |
| LOC_Os11g47300 | 4.549063764 | 0.021165428 | up |
| LOC_Os03g27090 | 4.544013426 | 0.035414938 | up |
| LOC_Os08g03190 | 4.540023355 | 0.0212221 | up |
| LOC_Os02g09560 | 4.53491698 | 0.03608489 | up |
| LOC_Os03g27410 | 4.51569633 | 0.041625667 | up |
| LOC_Os11g42470 | 4.476937356 | 0.040985942 | up |
| LOC_Os11g30290 | 4.456875461 | 0.026251763 | up |
| LOC_Os03g02230 | 4.447876246 | 0.000169519 | up |
| LOC_Os05g25330 | 4.442512619 | 0.02663177 | up |
| LOC_Os01g74430 | 4.437729791 | 0.033746353 | up |
| LOC_Os08g24650 | 4.418060588 | 0.029694325 | up |
| LOC_Os04g19810 | 4.413017944 | 0.032545041 | up |
| LOC_Os01g66900 | 4.402162104 | 0.048113586 | up |
| LOC_Os11g02220 | 4.396942512 | 0.049933364 | up |
| LOC_Os07g05450 | 4.366132526 | 5.03E-10 | up |
| LOC_Os01g19430 | 4.344612101 | 0.00846402 | up |
| LOC_Os07g05830 | 4.332606655 | 0.035281586 | up |
| LOC_Os04g44020 | 4.327338796 | 0.034819958 | up |
| LOC_Os05g32840 | 4.322097739 | 0.03605442 | up |
| LOC_Os08g11150 | 4.31900983 | 0.017736116 | up |
| LOC_Os12g31340 | 4.317122521 | 0.047254424 | up |
| LOC_Os01g10590 | 4.306421315 | 0.037242812 | up |
| LOC_Os07g42130 | 4.306363608 | 0.036373269 | up |
| LOC_Os09g27310 | 4.306363608 | 0.036373269 | up |
| LOC_Os11g01580 | 4.295701767 | 0.038868168 | up |
| LOC_Os07g10850 | 4.295173337 | 1.40E-05 | up |
| LOC_Os12g38720 | 4.287567896 | 5.91E-43 | up |
| LOC_Os04g01740 | 4.284770825 | 0.043786735 | up |
| LOC_Os06g21360 | 4.248042459 | 1.52E-19 | up |
| LOC_Os03g22130 | 4.234997066 | 0.000106883 | up |
| LOC_Os02g52730 | 4.192826173 | 2.66E-20 | up |
| LOC_Os05g02550 | 4.190932949 | 0.048040732 | up |
| LOC_Os04g34490 | 4.185711773 | 4.28E-33 | up |
| LOC_Os06g48090 | 4.173619964 | 0.048661842 | up |
| LOC_Os12g04140 | 4.143759913 | 0.017395145 | up |
| LOC_Os01g50350 | 4.137669637 | 0.015908847 | up |
| LOC_Os11g10710 | 4.108372534 | 3.23E-48 | up |
| LOC_Os01g62540 | 4.075142599 | 0.018172514 | up |
| LOC_Os11g02540 | 3.992669465 | 2.21E-07 | up |
| LOC_Os06g10020 | 3.989256559 | 0.00379372 | up |
| LOC_Os03g37610 | 3.986410302 | 0.023910297 | up |
| LOC_Os09g37890 | 3.986410302 | 0.023910297 | up |
| LOC_Os11g36790 | 3.980110829 | 0.000123267 | up |
| LOC_Os05g02030 | 3.966280186 | 0.023155517 | up |
| LOC_Os07g41400 | 3.917238561 | 1.33E-45 | up |
| LOC_Os01g56660 | 3.90273378 | 0.026343991 | up |
| LOC_Os02g09710 | 3.895797239 | 0.029858298 | up |
| LOC_Os03g26250 | 3.895671144 | 0.026108178 | up |
| LOC_Os12g05290 | 3.860567076 | 7.58E-07 | up |
| LOC_Os04g42240 | 3.837595396 | 0.049484215 | up |
| LOC_Os06g02350 | 3.798805307 | 0.033060073 | up |
| LOC_Os06g49350 | 3.792277549 | 2.13E-27 | up |
| LOC_Os06g14870 | 3.7833166 | 0.038568692 | up |
| LOC_Os01g49200 | 3.766470915 | 3.08E-05 | up |
| LOC_Os01g14630 | 3.751589688 | 1.54E-12 | up |
| LOC_Os04g05360 | 3.718435797 | 0.009751663 | up |
| LOC_Os11g02520 | 3.7156909 | 4.46E-09 | up |
| LOC_Os09g17000 | 3.715036882 | 0.041522304 | up |
| LOC_Os08g35720 | 3.711033741 | 0.041135797 | up |
| LOC_Os11g39380 | 3.706984058 | 0.041502941 | up |
| LOC_Os01g52900 | 3.682545215 | 0.045986188 | up |
| LOC_Os01g52690 | 3.639777771 | 7.60E-08 | up |
| LOC_Os12g43620 | 3.618549561 | 0.013722851 | up |
| LOC_Os05g08940 | 3.594244439 | 4.21E-05 | up |
| LOC_Os12g07950 | 3.541994915 | 1.45E-147 | up |
| LOC_Os03g25510 | 3.505108864 | 2.67E-07 | up |
| LOC_Os10g26110 | 3.489669985 | 2.10E-08 | up |
| LOC_Os03g03480 | 3.485668687 | 0.005441256 | up |
| LOC_Os02g58380 | 3.474179083 | 3.01E-07 | up |
| LOC_Os03g52020 | 3.473577008 | 0.029840477 | up |
| LOC_Os02g55900 | 3.455768583 | 0.019650481 | up |
| LOC_Os07g42560 | 3.412443382 | 1.94E-13 | up |
| LOC_Os02g58370 | 3.394967114 | 5.01E-18 | up |
| LOC_Os04g01560 | 3.312025498 | 0.029761933 | up |
| LOC_Os07g11440 | 3.311301268 | 3.72E-05 | up |
| LOC_Os01g71840 | 3.288544843 | 9.13E-10 | up |
| LOC_Os01g49650 | 3.285281249 | 0.002979824 | up |
| LOC_Os09g26760 | 3.271568964 | 0.033330413 | up |
| LOC_Os10g39990 | 3.247804549 | 0.047790031 | up |
| LOC_Os04g24510 | 3.227402034 | 0.001385676 | up |
| LOC_Os08g10240 | 3.214132772 | 0.048376608 | up |
| LOC_Os08g38800 | 3.187995712 | 2.83E-66 | up |
| LOC_Os02g41830 | 3.159683967 | 0.046695119 | up |
| LOC_Os04g10434 | 3.084544899 | 0.001713888 | up |
| LOC_Os01g03320 | 3.081359389 | 1.12E-18 | up |
| LOC_Os07g34260 | 3.056833485 | 7.98E-05 | up |
| LOC_Os12g37320 | 3.019172657 | 0.010906229 | up |
| LOC_Os07g39080 | 3.017318024 | 0.009421475 | up |
| LOC_Os01g08530 | 3.014110473 | 0.020626362 | up |
| LOC_Os06g13700 | 3.003255726 | 9.18E-19 | up |
| LOC_Os03g52070 | 2.992890057 | 1.23E-08 | up |
| LOC_Os07g34280 | 2.990141772 | 3.11E-06 | up |
| LOC_Os01g58100 | 2.984472093 | 7.98E-06 | up |
| LOC_Os06g07040 | 2.983884823 | 0.034652337 | up |
| LOC_Os07g19594 | 2.98302554 | 0.016162606 | up |
| LOC_Os10g07544 | 2.956013568 | 0.031476391 | up |
| LOC_Os10g02250 | 2.951660004 | 0.033201825 | up |
| LOC_Os03g42090 | 2.945946245 | 0.006163101 | up |
| LOC_Os07g44000 | 2.933275185 | 0.023120136 | up |
| LOC_Os10g02880 | 2.924246264 | 5.34E-17 | up |
| LOC_Os03g05700 | 2.911723645 | 0.005110479 | up |
| LOC_Os03g10290 | 2.901896732 | 1.12E-19 | up |
| LOC_Os01g16200 | 2.896520731 | 0.001561197 | up |
| LOC_Os03g01620 | 2.892125146 | 0.035974103 | up |
| LOC_Os11g27290 | 2.878972885 | 0.007001433 | up |
| LOC_Os01g01840 | 2.857583555 | 1.09E-09 | up |
| LOC_Os03g20780 | 2.846266657 | 0.014804187 | up |
| LOC_Os07g46920 | 2.845293666 | 0.000520834 | up |
| LOC_Os04g28270 | 2.844571442 | 0.008768827 | up |
| LOC_Os10g09110 | 2.83561599 | 0.00059601 | up |
| LOC_Os01g58490 | 2.824753922 | 0.040022434 | up |
| LOC_Os01g19190 | 2.814810154 | 0.000369653 | up |
| LOC_Os10g39190 | 2.810764657 | 4.34E-18 | up |
| LOC_Os09g03140 | 2.788004581 | 0.000281481 | up |
| LOC_Os04g29040 | 2.787407538 | 0.032216584 | up |
| LOC_Os08g31720 | 2.786708695 | 0.022562485 | up |
| LOC_Os03g08350 | 2.786486529 | 2.74E-05 | up |
| LOC_Os06g15110 | 2.712273746 | 0.006744332 | up |
| LOC_Os08g16359 | 2.66101442 | 0.030133766 | up |
| LOC_Os10g29400 | 2.646122137 | 0.007892404 | up |
| LOC_Os09g25380 | 2.62988686 | 0.034164428 | up |
| LOC_Os03g10280 | 2.622771434 | 0.011832429 | up |
| LOC_Os04g14690 | 2.620064703 | 2.30E-81 | up |
| LOC_Os08g03070 | 2.610489238 | 0.001769889 | up |
| LOC_Os02g48770 | 2.599676385 | 1.53E-32 | up |
| LOC_Os06g32720 | 2.588508506 | 0.005125957 | up |
| LOC_Os09g39630 | 2.587856228 | 0.007315127 | up |
| LOC_Os06g07941 | 2.581276695 | 0.000301675 | up |
| LOC_Os07g03750 | 2.573306483 | 0.030241511 | up |
| LOC_Os03g60960 | 2.569999614 | 0.003523339 | up |
| LOC_Os04g17660 | 2.555605143 | 2.20E-20 | up |
| LOC_Os12g18680 | 2.539435069 | 0.030343475 | up |
| LOC_Os07g49090 | 2.532761612 | 0.010050822 | up |
| LOC_Os03g30740 | 2.528296183 | 7.05E-09 | up |
| LOC_Os02g32630 | 2.522309592 | 0.037505328 | up |
| LOC_Os06g42120 | 2.507040769 | 0.049370073 | up |
| LOC_Os08g04340 | 2.484635813 | 0.001352119 | up |
| LOC_Os01g42370 | 2.472993492 | 2.82E-05 | up |
| LOC_Os04g09604 | 2.444097029 | 8.37E-28 | up |
| LOC_Os04g32010 | 2.425330104 | 0.000128943 | up |
| LOC_Os07g02920 | 2.423248713 | 0.012616126 | up |
| LOC_Os05g43940 | 2.420091832 | 0.022742617 | up |
| LOC_Os09g08720 | 2.407261967 | 6.57E-06 | up |
| LOC_Os01g28450 | 2.402832612 | 0.008905524 | up |
| LOC_Os03g18030 | 2.383226933 | 5.75E-11 | up |
| LOC_Os10g08810 | 2.377733331 | 0.026657644 | up |
| LOC_Os05g44650 | 2.368377245 | 0.024377988 | up |
| LOC_Os07g01904 | 2.343760806 | 3.82E-72 | up |
| LOC_Os08g41590 | 2.317766543 | 5.24E-25 | up |
| LOC_Os04g58040 | 2.31376981 | 0.006399288 | up |
| LOC_Os10g37020 | 2.311577237 | 0.023195734 | up |
| LOC_Os12g04230 | 2.304678375 | 0.022659374 | up |
| LOC_Os06g05310 | 2.294759997 | 0.00426395 | up |
| LOC_Os10g04370 | 2.277742717 | 0.000135872 | up |
| LOC_Os08g41600 | 2.276408788 | 2.38E-10 | up |
| LOC_Os09g25390 | 2.262370531 | 0.001192878 | up |
| LOC_Os06g09980 | 2.25384311 | 8.19E-07 | up |
| LOC_Os02g10240 | 2.246472632 | 0.014528141 | up |
| LOC_Os03g47280 | 2.242902023 | 2.03E-07 | up |
| LOC_Os04g30720 | 2.238170009 | 0.002586916 | up |
| LOC_Os09g19140 | 2.238157446 | 9.75E-13 | up |
| LOC_Os01g52410 | 2.234578036 | 0.00068133 | up |
| LOC_Os03g42780 | 2.232375128 | 0.001322119 | up |
| LOC_Os04g47580 | 2.222805981 | 0.000576361 | up |
| LOC_Os05g02230 | 2.202118846 | 0.029681566 | up |
| LOC_Os01g56235 | 2.193955736 | 0.024626218 | up |
| LOC_Os10g41270 | 2.180462791 | 0.002285977 | up |
| LOC_Os09g16790 | 2.169320689 | 1.35E-07 | up |
| LOC_Os09g38710 | 2.162133797 | 0.001098308 | up |
| LOC_Os04g27340 | 2.160808602 | 1.71E-05 | up |
| LOC_Os02g11870 | 2.158585467 | 0.002155059 | up |
| LOC_Os12g02400 | 2.152151175 | 0.019029756 | up |
| LOC_Os08g07360 | 2.14886817 | 0.028561959 | up |
| LOC_Os04g40750 | 2.14124859 | 0.039072125 | up |
| LOC_Os10g02840 | 2.11778508 | 3.11E-06 | up |
| LOC_Os08g17500 | 2.115936521 | 0.018234415 | up |
| LOC_Os08g31130 | 2.113756329 | 0.01017825 | up |
| LOC_Os11g35930 | 2.109770543 | 1.56E-17 | up |
| LOC_Os08g35990 | 2.103936224 | 0.036830626 | up |
| LOC_Os02g56010 | 2.103393991 | 0.047257978 | up |
| LOC_Os03g57510 | 2.102992854 | 0.046942641 | up |
| LOC_Os01g20206 | 2.1006843 | 7.17E-06 | up |
| LOC_Os05g50820 | 2.085212018 | 0.012390623 | up |
| LOC_Os01g33070 | 2.076402225 | 0.031634749 | up |
| LOC_Os01g04050 | 2.062463699 | 0.00013683 | up |
| LOC_Os06g16000 | 2.062177384 | 0.012607552 | up |
| LOC_Os03g03810 | 2.062058566 | 0.00013718 | up |
| LOC_Os04g15920 | 2.044067419 | 1.59E-18 | up |
| LOC_Os10g33774 | 2.036939406 | 0.014345504 | up |
| LOC_Os02g57770 | 2.004179498 | 4.27E-09 | up |
| LOC_Os10g02230 | 2.002543979 | 0.024995614 | up |
| LOC_Os05g44060 | 2.001885302 | 5.65E-08 | up |
| LOC_Os07g41060 | 1.996442728 | 0.000509667 | up |
| LOC_Os03g08320 | 1.992012969 | 4.80E-16 | up |
| LOC_Os05g20020 | 1.987968707 | 0.024283388 | up |
| LOC_Os03g29150 | 1.98612946 | 0.006486044 | up |
| LOC_Os08g36960 | 1.984544432 | 0.037055412 | up |
| LOC_Os06g08800 | 1.979425488 | 0.047875452 | up |
| LOC_Os09g26780 | 1.972096159 | 9.92E-20 | up |
| LOC_Os08g38650 | 1.970205983 | 0.015927123 | up |
| LOC_Os09g09540 | 1.9678598 | 0.007194283 | up |
| LOC_Os06g13460 | 1.955994355 | 0.039181329 | up |
| LOC_Os10g30360 | 1.921572476 | 0.004152962 | up |
| LOC_Os12g25350 | 1.911250429 | 5.23E-05 | up |
| LOC_Os02g06630 | 1.885572622 | 1.43E-08 | up |
| LOC_Os05g24240 | 1.885202905 | 0.03892666 | up |
| LOC_Os01g71490 | 1.884334449 | 0.015691185 | up |
| LOC_Os10g22110 | 1.870576239 | 0.046759698 | up |
| LOC_Os03g55460 | 1.868633074 | 0.007276032 | up |
| LOC_Os02g15810 | 1.860162193 | 0.006368964 | up |
| LOC_Os05g50610 | 1.859566292 | 1.33E-05 | up |
| LOC_Os08g14880 | 1.853747876 | 6.63E-49 | up |
| LOC_Os02g42970 | 1.853550521 | 0.010640642 | up |
| LOC_Os02g47060 | 1.844327876 | 0.009577604 | up |
| LOC_Os07g29570 | 1.831325126 | 0.001625557 | up |
| LOC_Os10g35950 | 1.826835272 | 0.000123295 | up |
| LOC_Os01g01950 | 1.821911395 | 0.037815124 | up |
| LOC_Os11g44890 | 1.817833699 | 0.004996107 | up |
| LOC_Os05g38980 | 1.817803487 | 0.002310367 | up |
| LOC_Os01g73604 | 1.811629164 | 0.031015779 | up |
| LOC_Os05g39750 | 1.804063316 | 0.007249826 | up |
| LOC_Os07g03740 | 1.796731233 | 0.003970515 | up |
| LOC_Os03g52860 | 1.785339955 | 0.017684648 | up |
| LOC_Os08g06510 | 1.782510532 | 0.00493676 | up |
| LOC_Os07g41650 | 1.781041536 | 0.007020756 | up |
| LOC_Os09g22410 | 1.774375483 | 5.02E-15 | up |
| LOC_Os02g09260 | 1.76551469 | 0.01455906 | up |
| LOC_Os11g42220 | 1.760644184 | 0.012125685 | up |
| LOC_Os01g19200 | 1.753494991 | 0.022134668 | up |
| LOC_Os01g68050 | 1.744214103 | 4.26E-14 | up |
| LOC_Os01g12240 | 1.738529577 | 0.000490158 | up |
| LOC_Os09g25070 | 1.735868003 | 0.001766308 | up |
| LOC_Os05g14260 | 1.729513127 | 0.035491537 | up |
| LOC_Os03g10469 | 1.726627106 | 0.047609601 | up |
| LOC_Os09g03900 | 1.7254984 | 0.021025171 | up |
| LOC_Os10g11270 | 1.724674554 | 7.84E-08 | up |
| LOC_Os09g28660 | 1.720005135 | 0.023247637 | up |
| LOC_Os01g15520 | 1.711829713 | 4.03E-18 | up |
| LOC_Os04g28620 | 1.707861088 | 3.64E-23 | up |
| LOC_Os10g28350 | 1.707806481 | 4.79E-05 | up |
| LOC_Os08g30340 | 1.705143637 | 0.021469022 | up |
| LOC_Os01g70490 | 1.682284811 | 8.82E-10 | up |
| LOC_Os04g32810 | 1.681515579 | 1.05E-15 | up |
| LOC_Os10g33104 | 1.670745573 | 5.45E-07 | up |
| LOC_Os07g35500 | 1.664826862 | 0.003668458 | up |
| LOC_Os04g32830 | 1.657185011 | 0.001035444 | up |
| LOC_Os01g05310 | 1.646412051 | 0.02609227 | up |
| LOC_Os03g37140 | 1.644040054 | 0.044058383 | up |
| LOC_Os12g35490 | 1.640675715 | 0.000397592 | up |
| LOC_Os08g40180 | 1.630465647 | 1.03E-15 | up |
| LOC_Os12g05690 | 1.626865872 | 0.036104427 | up |
| LOC_Os01g50370 | 1.626439096 | 0.002453059 | up |
| LOC_Os06g34550 | 1.626056696 | 0.029910283 | up |
| LOC_Os03g10440 | 1.600191782 | 0.008993853 | up |
| LOC_Os04g53800 | 1.592998046 | 1.13E-09 | up |
| LOC_Os09g32210 | 1.590912489 | 0.014758932 | up |
| LOC_Os12g29160 | 1.587846201 | 0.013101259 | up |
| LOC_Os10g29800 | 1.584832928 | 0.015023904 | up |
| LOC_Os11g41380 | 1.582128402 | 2.02E-13 | up |
| LOC_Os06g23180 | 1.576152031 | 0.027773276 | up |
| LOC_Os02g53580 | 1.575240394 | 0.018778329 | up |
| LOC_Os04g33820 | 1.57397371 | 0.002906836 | up |
| LOC_Os04g44240 | 1.573553518 | 0.000476153 | up |
| LOC_Os01g38580 | 1.56191055 | 0.000150477 | up |
| LOC_Os01g03620 | 1.56143246 | 3.88E-06 | up |
| LOC_Os07g09190 | 1.559756084 | 3.50E-11 | up |
| LOC_Os01g49720 | 1.558056274 | 4.44E-05 | up |
| LOC_Os08g01690 | 1.553448655 | 0.003323376 | up |
| LOC_Os07g37730 | 1.551935626 | 2.06E-07 | up |
| LOC_Os04g35270 | 1.544918979 | 0.016396044 | up |
| LOC_Os12g17950 | 1.540579364 | 3.83E-05 | up |
| LOC_Os01g16270 | 1.529425482 | 0.032447054 | up |
| LOC_Os12g41730 | 1.523056635 | 0.003603826 | up |
| LOC_Os05g46840 | 1.519325941 | 0.006443033 | up |
| LOC_Os11g04030 | 1.511228969 | 0.000162444 | up |
| LOC_Os09g34330 | 1.510430435 | 0.001869305 | up |
| LOC_Os08g28890 | 1.510395376 | 0.049212101 | up |
| LOC_Os08g11180 | 1.50988572 | 0.004594851 | up |
| LOC_Os07g18154 | 1.501083143 | 3.26E-05 | up |
| LOC_Os01g18120 | 1.495670403 | 0.000317816 | up |
| LOC_Os02g43050 | 1.489650833 | 0.033660424 | up |
| LOC_Os08g07730 | 1.486911868 | 0.024430075 | up |
| LOC_Os10g12140 | 1.486777688 | 0.017415944 | up |
| LOC_Os08g36230 | 1.48478926 | 0.016772028 | up |
| LOC_Os01g66010 | 1.482440413 | 4.70E-06 | up |
| LOC_Os02g38000 | 1.472411527 | 0.002464386 | up |
| LOC_Os04g32480 | 1.468809492 | 0.00827517 | up |
| LOC_Os04g32840 | 1.468691787 | 0.00359339 | up |
| LOC_Os06g21270 | 1.467604281 | 0.010553084 | up |
| LOC_Os08g11200 | 1.466273223 | 0.005587461 | up |
| LOC_Os07g27350 | 1.465465451 | 1.87E-18 | up |
| LOC_Os03g28110 | 1.462669865 | 0.005535142 | up |
| LOC_Os01g08710 | 1.459016425 | 0.000111116 | up |
| LOC_Os05g26040 | 1.458694691 | 2.27E-09 | up |
| LOC_Os11g47944 | 1.449983786 | 0.014082806 | up |
| LOC_Os08g29560 | 1.449547332 | 0.001035079 | up |
| LOC_Os01g66760 | 1.448502146 | 0.026902383 | up |
| LOC_Os03g38470 | 1.444186965 | 0.040630063 | up |
| LOC_Os03g58290 | 1.443698079 | 6.89E-14 | up |
| LOC_Os08g43570 | 1.443635343 | 0.022088147 | up |
| LOC_Os08g03020 | 1.44025318 | 1.97E-09 | up |
| LOC_Os07g18162 | 1.438273341 | 4.50E-13 | up |
| LOC_Os05g50170 | 1.432853668 | 0.018473053 | up |
| LOC_Os07g30330 | 1.428069386 | 0.003163665 | up |
| LOC_Os09g26170 | 1.426277535 | 0.009085731 | up |
| LOC_Os04g32860 | 1.424310386 | 5.48E-17 | up |
| LOC_Os11g29920 | 1.41410472 | 0.027933432 | up |
| LOC_Os05g43930 | 1.412419405 | 0.00047253 | up |
| LOC_Os07g47960 | 1.41001971 | 0.000630531 | up |
| LOC_Os09g21160 | 1.408413192 | 0.047700093 | up |
| LOC_Os10g04520 | 1.407409841 | 0.002747184 | up |
| LOC_Os05g45140 | 1.405029861 | 0.006404495 | up |
| LOC_Os04g42950 | 1.393645398 | 0.002424251 | up |
| LOC_Os01g32380 | 1.392277138 | 0.049339596 | up |
| LOC_Os07g13770 | 1.392157187 | 0.000442896 | up |
| LOC_Os04g35430 | 1.39178616 | 0.011196373 | up |
| LOC_Os01g49710 | 1.382101483 | 0.002345862 | up |
| LOC_Os11g42240 | 1.381539881 | 0.025679914 | up |
| LOC_Os02g31845 | 1.377061174 | 0.013318565 | up |
| LOC_Os10g33040 | 1.37521609 | 0.016436943 | up |
| LOC_Os06g49190 | 1.373701668 | 6.36E-15 | up |
| LOC_Os05g14590 | 1.372368773 | 0.023478595 | up |
| LOC_Os10g03090 | 1.371762705 | 0.023594495 | up |
| LOC_Os09g02250 | 1.371597768 | 6.56E-05 | up |
| LOC_Os04g08450 | 1.367065925 | 0.002063586 | up |
| LOC_Os10g36960 | 1.365088046 | 0.000133115 | up |
| LOC_Os05g10370 | 1.357445315 | 8.87E-07 | up |
| LOC_Os08g13420 | 1.355890721 | 0.003819137 | up |
| LOC_Os07g37110 | 1.35418975 | 7.68E-05 | up |
| LOC_Os06g11190 | 1.349492549 | 6.30E-14 | up |
| LOC_Os02g58360 | 1.348179468 | 2.50E-10 | up |
| LOC_Os06g51200 | 1.343462635 | 0.023727661 | up |
| LOC_Os09g03939 | 1.340337549 | 0.018796073 | up |
| LOC_Os07g34070 | 1.336273576 | 0.000598449 | up |
| LOC_Os04g23550 | 1.336020181 | 1.29E-05 | up |
| LOC_Os12g22110 | 1.334998491 | 4.72E-10 | up |
| LOC_Os04g03990 | 1.327192276 | 0.002078785 | up |
| LOC_Os03g01014 | 1.326539861 | 0.043486336 | up |
| LOC_Os12g33194 | 1.324241018 | 5.27E-07 | up |
| LOC_Os02g43820 | 1.321765362 | 0.04374871 | up |
| LOC_Os09g30280 | 1.317519493 | 0.010468806 | up |
| LOC_Os08g28710 | 1.315815974 | 0.012312394 | up |
| LOC_Os10g35500 | 1.310765354 | 0.000403466 | up |
| LOC_Os01g71570 | 1.306633613 | 0.013940681 | up |
| LOC_Os03g24760 | 1.30244587 | 0.013591691 | up |
| LOC_Os01g36240 | 1.299471215 | 0.000106744 | up |
| LOC_Os11g17540 | 1.290717966 | 2.22E-10 | up |
| LOC_Os12g37260 | 1.289753909 | 0.000704029 | up |
| LOC_Os05g35360 | 1.2858553 | 5.58E-07 | up |
| LOC_Os03g61720 | 1.284825396 | 4.30E-14 | up |
| LOC_Os09g37600 | 1.28378651 | 0.001381641 | up |
| LOC_Os10g28230 | 1.276370748 | 0.004240721 | up |
| LOC_Os04g55130 | 1.274824703 | 0.01215508 | up |
| LOC_Os10g25400 | 1.274250962 | 0.006943917 | up |
| LOC_Os01g25500 | 1.268740029 | 0.006690705 | up |
| LOC_Os03g58300 | 1.268038802 | 4.05E-10 | up |
| LOC_Os09g17146 | 1.267786623 | 0.039664316 | up |
| LOC_Os05g10830 | 1.264756746 | 6.05E-05 | up |
| LOC_Os01g70220 | 1.264292809 | 1.26E-05 | up |
| LOC_Os05g08850 | 1.264209303 | 0.001879543 | up |
| LOC_Os01g70340 | 1.260719052 | 8.30E-05 | up |
| LOC_Os06g39390 | 1.259840165 | 1.03E-13 | up |
| LOC_Os11g22350 | 1.258949784 | 8.13E-12 | up |
| LOC_Os01g60600 | 1.257157855 | 0.030578362 | up |
| LOC_Os03g57640 | 1.253860133 | 0.011172643 | up |
| LOC_Os03g15180 | 1.251158172 | 0.042183459 | up |
| LOC_Os01g18390 | 1.247864937 | 0.042286299 | up |
| LOC_Os04g51520 | 1.243905278 | 0.015946014 | up |
| LOC_Os11g45990 | 1.243453427 | 0.000116089 | up |
| LOC_Os07g13780 | 1.242549277 | 0.000350262 | up |
| LOC_Os06g38350 | 1.240868834 | 8.12E-05 | up |
| LOC_Os08g11190 | 1.24074495 | 0.011783957 | up |
| LOC_Os11g05840 | 1.23586906 | 0.011335595 | up |
| LOC_Os03g19670 | 1.23582033 | 0.04028301 | up |
| LOC_Os01g47820 | 1.228301742 | 0.030712686 | up |
| LOC_Os07g18158 | 1.226797291 | 2.68E-09 | up |
| LOC_Os08g04630 | 1.225678148 | 0.00010184 | up |
| LOC_Os01g50940 | 1.224585977 | 1.32E-06 | up |
| LOC_Os04g50770 | 1.22405313 | 3.96E-05 | up |
| LOC_Os12g01830 | 1.220270195 | 0.006523007 | up |
| LOC_Os06g46990 | 1.218926276 | 0.045268599 | up |
| LOC_Os11g36830 | 1.215408948 | 4.81E-15 | up |
| LOC_Os01g61690 | 1.209987592 | 0.033918495 | up |
| LOC_Os05g46350 | 1.20875662 | 0.000216977 | up |
| LOC_Os01g19460 | 1.207080707 | 0.022889729 | up |
| LOC_Os02g58350 | 1.204448886 | 0.004133461 | up |
| LOC_Os12g09540 | 1.202007198 | 0.041136932 | up |
| LOC_Os11g36810 | 1.201682684 | 0.003507777 | up |
| LOC_Os07g22400 | 1.199130153 | 0.016720217 | up |
| LOC_Os11g42550 | 1.198406298 | 6.64E-10 | up |
| LOC_Os05g24560 | 1.195896293 | 0.033983205 | up |
| LOC_Os03g09910 | 1.195724129 | 0.034654308 | up |
| LOC_Os05g32830 | 1.19326751 | 0.003988639 | up |
| LOC_Os09g20684 | 1.192292628 | 9.12E-07 | up |
| LOC_Os03g03790 | 1.186710769 | 7.43E-09 | up |
| LOC_Os07g44250 | 1.186581492 | 0.000136252 | up |
| LOC_Os11g29000 | 1.185771155 | 0.00253329 | up |
| LOC_Os11g29290 | 1.182851368 | 1.52E-08 | up |
| LOC_Os07g13160 | 1.180241616 | 0.01138017 | up |
| LOC_Os01g42690 | 1.180007853 | 0.014652613 | up |
| LOC_Os06g38340 | 1.178173803 | 2.41E-14 | up |
| LOC_Os02g09790 | 1.176380232 | 0.001883727 | up |
| LOC_Os02g08270 | 1.175589989 | 0.032843811 | up |
| LOC_Os10g42130 | 1.1727654 | 0.000348269 | up |
| LOC_Os12g38040 | 1.169198833 | 0.001576017 | up |
| LOC_Os10g08790 | 1.164384678 | 0.007675508 | up |
| LOC_Os02g32009 | 1.163320837 | 0.018813203 | up |
| LOC_Os04g32820 | 1.162679551 | 4.37E-09 | up |
| LOC_Os06g21570 | 1.162165236 | 0.01937666 | up |
| LOC_Os01g61570 | 1.161907324 | 0.007091776 | up |
| LOC_Os01g41950 | 1.161614848 | 0.035477377 | up |
| LOC_Os08g03700 | 1.153695015 | 0.020254883 | up |
| LOC_Os03g35600 | 1.151185299 | 2.65E-06 | up |
| LOC_Os09g16780 | 1.150713354 | 0.000781283 | up |
| LOC_Os06g19070 | 1.149555628 | 2.72E-06 | up |
| LOC_Os10g34480 | 1.14881347 | 1.61E-10 | up |
| LOC_Os03g24650 | 1.148273858 | 0.019607356 | up |
| LOC_Os07g13790 | 1.145995446 | 0.016751531 | up |
| LOC_Os07g40290 | 1.144501627 | 7.23E-05 | up |
| LOC_Os05g45240 | 1.143921375 | 0.00440408 | up |
| LOC_Os01g01940 | 1.13702592 | 0.030111994 | up |
| LOC_Os11g26950 | 1.134845755 | 6.52E-05 | up |
| LOC_Os07g05190 | 1.134556982 | 0.005106498 | up |
| LOC_Os08g32920 | 1.133698303 | 0.006656745 | up |
| LOC_Os06g30430 | 1.133406814 | 0.013028071 | up |
| LOC_Os02g18880 | 1.131167844 | 0.000558216 | up |
| LOC_Os10g01920 | 1.126727689 | 0.045916318 | up |
| LOC_Os06g07300 | 1.126506925 | 0.000225444 | up |
| LOC_Os09g21260 | 1.126410234 | 0.0376956 | up |
| LOC_Os12g32970 | 1.125169318 | 9.39E-06 | up |
| LOC_Os07g44690 | 1.123596614 | 0.001194848 | up |
| LOC_Os10g04400 | 1.123269836 | 0.013605322 | up |
| LOC_Os01g49230 | 1.122700503 | 0.023013835 | up |
| LOC_Os09g36700 | 1.121208658 | 0.004962692 | up |
| LOC_Os12g40750 | 1.120644258 | 1.73E-05 | up |
| LOC_Os12g25660 | 1.120115935 | 0.004507748 | up |
| LOC_Os11g42500 | 1.113333857 | 0.002364061 | up |
| LOC_Os10g25290 | 1.112859661 | 4.79E-08 | up |
| LOC_Os12g04340 | 1.110998684 | 0.01627681 | up |
| LOC_Os01g16714 | 1.109589431 | 2.37E-07 | up |
| LOC_Os07g22770 | 1.108891231 | 0.000841905 | up |
| LOC_Os12g03410 | 1.108800983 | 0.035412488 | up |
| LOC_Os01g65690 | 1.108453283 | 0.000318269 | up |
| LOC_Os08g38210 | 1.105111331 | 1.67E-08 | up |
| LOC_Os05g50790 | 1.100634608 | 0.00649435 | up |
| LOC_Os06g34730 | 1.099877675 | 6.03E-06 | up |
| LOC_Os06g36560 | 1.099790304 | 2.92E-09 | up |
| LOC_Os03g39650 | 1.096136464 | 0.014703639 | up |
| LOC_Os09g10010 | 1.094477229 | 0.001078384 | up |
| LOC_Os11g10180 | 1.092100359 | 0.000232203 | up |
| LOC_Os09g39370 | 1.089464829 | 0.019526706 | up |
| LOC_Os09g39760 | 1.081978021 | 0.01516028 | up |
| LOC_Os06g50360 | 1.080941851 | 1.12E-08 | up |
| LOC_Os03g26530 | 1.079284333 | 0.004148023 | up |
| LOC_Os04g53780 | 1.073601697 | 0.000127411 | up |
| LOC_Os02g13310 | 1.072561812 | 0.024971942 | up |
| LOC_Os06g28800 | 1.07010361 | 0.007980934 | up |
| LOC_Os07g46770 | 1.070005521 | 0.012544244 | up |
| LOC_Os08g02996 | 1.067509098 | 2.80E-07 | up |
| LOC_Os02g37770 | 1.06739879 | 3.52E-07 | up |
| LOC_Os06g06510 | 1.064818499 | 0.0305245 | up |
| LOC_Os11g22960 | 1.063207568 | 0.023321863 | up |
| LOC_Os01g24710 | 1.062647224 | 0.008409376 | up |
| LOC_Os01g46140 | 1.06122139 | 2.43E-05 | up |
| LOC_Os11g30810 | 1.060420515 | 0.000942334 | up |
| LOC_Os02g43194 | 1.060346718 | 4.28E-13 | up |
| LOC_Os07g03150 | 1.059681563 | 0.01436114 | up |
| LOC_Os05g23950 | 1.057229464 | 0.010414609 | up |
| LOC_Os07g15160 | 1.05306473 | 0.000758303 | up |
| LOC_Os07g34320 | 1.05022225 | 9.02E-05 | up |
| LOC_Os12g12514 | 1.04997817 | 0.000339718 | up |
| LOC_Os09g33700 | 1.049459205 | 0.028346515 | up |
| LOC_Os04g26870 | 1.049217792 | 0.003477365 | up |
| LOC_Os03g52284 | 1.046071743 | 0.026454704 | up |
| LOC_Os05g49730 | 1.044701778 | 0.028365484 | up |
| LOC_Os06g05630 | 1.044257786 | 0.00412958 | up |
| LOC_Os01g22560 | 1.04089963 | 7.96E-07 | up |
| LOC_Os07g03810 | 1.034455013 | 3.79E-06 | up |
| LOC_Os05g37190 | 1.033591895 | 0.001205508 | up |
| LOC_Os02g55330 | 1.031187688 | 0.000308499 | up |
| LOC_Os04g47010 | 1.030286049 | 0.027551764 | up |
| LOC_Os05g01010 | 1.028539351 | 6.61E-05 | up |
| LOC_Os03g01290 | 1.027729448 | 0.000382163 | up |
| LOC_Os12g38540 | 1.024518182 | 0.03135529 | up |
| LOC_Os05g49780 | 1.024041647 | 0.011668897 | up |
| LOC_Os09g25060 | 1.023246556 | 0.000896328 | up |
| LOC_Os02g11859 | 1.022859446 | 0.000831604 | up |
| LOC_Os06g05930 | 1.022317171 | 0.003938572 | up |
| LOC_Os04g32800 | 1.021921548 | 3.19E-08 | up |
| LOC_Os02g26014 | 1.021866104 | 0.042103156 | up |
| LOC_Os12g22120 | 1.021561145 | 0.014382038 | up |
| LOC_Os02g36500 | 1.021262544 | 0.010195884 | up |
| LOC_Os03g55070 | 1.018908093 | 1.05E-13 | up |
| LOC_Os11g03300 | 1.015618203 | 2.80E-05 | up |
| LOC_Os06g34650 | 1.014511018 | 0.002686732 | up |
| LOC_Os03g08330 | 1.010666398 | 4.54E-09 | up |
| LOC_Os07g42430 | 1.008162522 | 0.000674837 | up |
| LOC_Os03g61620 | 1.007824792 | 7.51E-08 | up |
| LOC_Os04g45100 | 1.00706344 | 0.005449165 | up |
| LOC_Os02g37780 | 1.006331957 | 0.000147675 | up |
| LOC_Os04g56060 | 1.005450222 | 4.31E-11 | up |
| LOC_Os01g19480 | 1.004214774 | 7.07E-07 | up |
| LOC_Os03g63560 | 1.003797669 | 0.000103465 | up |
| LOC_Os07g23640 | 1.001927387 | 0.0035215 | up |
| LOC_Os02g05900 | 1.001922561 | 2.28E-06 | up |
| LOC_Os08g05610 | 1.001846164 | 0.011481302 | up |
| LOC_Os03g06670 | 1.001666228 | 0.010150707 | up |
| LOC_Os04g47720 | 1.001395481 | 0.045963117 | up |
| LOC_Os01g04310 | -4.453891765 | 0.025332857 | down |
| LOC_Os01g04520 | -3.125553352 | 0.047885201 | down |
| LOC_Os01g05640 | -1.400393109 | 0.013171366 | down |
| LOC_Os01g09700 | -1.555467464 | 0.016723953 | down |
| LOC_Os01g11730 | -1.45001766 | 2.73E-06 | down |
| LOC_Os01g13120 | -1.124134007 | 1.61E-14 | down |
| LOC_Os01g13610 | -6.844397481 | 7.00E-06 | down |
| LOC_Os01g14550 | -1.471080889 | 0.028657278 | down |
| LOC_Os01g15240 | -1.352529463 | 0.004715926 | down |
| LOC_Os01g17330 | -2.467094766 | 0.04616495 | down |
| LOC_Os01g19290 | -6.173248783 | 3.97E-05 | down |
| LOC_Os01g23710 | -1.336510531 | 7.45E-08 | down |
| LOC_Os01g27230 | -1.522175473 | 8.34E-24 | down |
| LOC_Os01g28580 | -1.876926308 | 0.003143981 | down |
| LOC_Os01g28980 | -1.553864407 | 0.010173997 | down |
| LOC_Os01g29804 | -2.159161328 | 8.69E-09 | down |
| LOC_Os01g31010 | -5.025439421 | 0.004430532 | down |
| LOC_Os01g31020 | -2.98975194 | 8.32E-06 | down |
| LOC_Os01g31050 | -2.750520572 | 1.50E-07 | down |
| LOC_Os01g32920 | -2.131751878 | 0.005950671 | down |
| LOC_Os01g36294 | -2.681556634 | 1.26E-10 | down |
| LOC_Os01g40290 | -3.933516918 | 0.030148473 | down |
| LOC_Os01g40800 | -4.5912836 | 0.018674801 | down |
| LOC_Os01g41790 | -5.698476768 | 0.000336725 | down |
| LOC_Os01g42860 | -1.296566368 | 0.016095243 | down |
| LOC_Os01g44360 | -1.266355817 | 0.035758884 | down |
| LOC_Os01g45140 | -1.110535181 | 0.000151708 | down |
| LOC_Os01g45274 | -1.102283177 | 6.41E-20 | down |
| LOC_Os01g45914 | -2.154439198 | 5.23E-46 | down |
| LOC_Os01g46460 | -4.553135834 | 0.034457536 | down |
| LOC_Os01g47370 | -1.823331905 | 3.42E-08 | down |
| LOC_Os01g48120 | -5.172705895 | 0.002774147 | down |
| LOC_Os01g49320 | -1.139923635 | 0.000211865 | down |
| LOC_Os01g53779 | -1.162611031 | 4.08E-05 | down |
| LOC_Os01g56960 | -1.498608148 | 0.010655101 | down |
| LOC_Os01g57030 | -2.250161775 | 0.031549494 | down |
| LOC_Os01g57599 | -1.107431738 | 1.51E-07 | down |
| LOC_Os01g58022 | -1.28047385 | 0.02157343 | down |
| LOC_Os01g63540 | -1.364058149 | 4.88E-06 | down |
| LOC_Os01g68290 | -4.552396703 | 6.87E-09 | down |
| LOC_Os01g68720 | -2.163249047 | 0.000835516 | down |
| LOC_Os01g68740 | -1.186604646 | 0.001170604 | down |
| LOC_Os01g69010 | -2.557659817 | 2.22E-05 | down |
| LOC_Os01g72370 | -2.97399453 | 2.00E-20 | down |
| LOC_Os01g73390 | -1.588146018 | 0.017137117 | down |
| LOC_Os01g74160 | -1.091811533 | 0.000222876 | down |
| LOC_Os01g74170 | -1.433397866 | 0.000595706 | down |
| LOC_Os01g74340 | -1.381038251 | 0.000533765 | down |
| LOC_Os02g06140 | -1.063294828 | 0.010572008 | down |
| LOC_Os02g06420 | -1.500158528 | 0.042330216 | down |
| LOC_Os02g09760 | -3.678453783 | 0.044497771 | down |
| LOC_Os02g10450 | -1.450316199 | 0.013848927 | down |
| LOC_Os02g10780 | -1.81566533 | 1.44E-36 | down |
| LOC_Os02g11790 | -1.279406933 | 1.04E-06 | down |
| LOC_Os02g12680 | -4.008949745 | 0.021662989 | down |
| LOC_Os02g13460 | -1.411791387 | 0.043012445 | down |
| LOC_Os02g13620 | -2.728087003 | 0.001782452 | down |
| LOC_Os02g14180 | -3.713241138 | 0.040221466 | down |
| LOC_Os02g14440 | -1.320622995 | 3.66E-18 | down |
| LOC_Os02g16544 | -4.616809188 | 0.02964888 | down |
| LOC_Os02g19410 | -1.362948309 | 0.043493891 | down |
| LOC_Os02g20210 | -1.703748722 | 0.00649733 | down |
| LOC_Os02g21470 | -1.158651977 | 0.008477379 | down |
| LOC_Os02g27120 | -4.338364338 | 0.034887842 | down |
| LOC_Os02g28970 | -3.751773868 | 0.025992655 | down |
| LOC_Os02g29190 | -2.144678696 | 0.048567645 | down |
| LOC_Os02g29774 | -2.155781828 | 0.011535031 | down |
| LOC_Os02g34970 | -1.087772503 | 0.024079184 | down |
| LOC_Os02g34990 | -1.129099003 | 1.71E-11 | down |
| LOC_Os02g36070 | -3.463845372 | 0.004875918 | down |
| LOC_Os02g36110 | -1.23585259 | 0.048068215 | down |
| LOC_Os02g36140 | -2.09393734 | 0.020780899 | down |
| LOC_Os02g36190 | -3.226997149 | 0.003197746 | down |
| LOC_Os02g36210 | -2.098346045 | 9.53E-12 | down |
| LOC_Os02g38574 | -1.90527592 | 0.028342046 | down |
| LOC_Os02g39090 | -1.008274509 | 5.79E-05 | down |
| LOC_Os02g43370 | -2.405325877 | 2.61E-35 | down |
| LOC_Os02g46740 | -1.267558022 | 0.019832142 | down |
| LOC_Os02g46820 | -3.685771823 | 0.045795121 | down |
| LOC_Os02g48450 | -1.719185456 | 0.006736603 | down |
| LOC_Os02g50000 | -1.347943752 | 9.41E-07 | down |
| LOC_Os02g50460 | -1.301855807 | 0.033410291 | down |
| LOC_Os02g51510 | -1.651538127 | 0.006310665 | down |
| LOC_Os02g51910 | -2.051742381 | 0.003720875 | down |
| LOC_Os02g53840 | -1.676436753 | 0.046541164 | down |
| LOC_Os02g55710 | -1.406466914 | 0.004475871 | down |
| LOC_Os03g04040 | -1.023612809 | 0.000694649 | down |
| LOC_Os03g04190 | -1.723376339 | 0.002321542 | down |
| LOC_Os03g08624 | -1.332848757 | 0.023109061 | down |
| LOC_Os03g10260 | -1.082763755 | 0.040519162 | down |
| LOC_Os03g17790 | -1.004156166 | 0.037532049 | down |
| LOC_Os03g17870 | -1.595272909 | 2.63E-08 | down |
| LOC_Os03g18060 | -2.293870986 | 0.024402029 | down |
| LOC_Os03g18130 | -1.221827836 | 1.19E-05 | down |
| LOC_Os03g18640 | -1.444987759 | 0.018231335 | down |
| LOC_Os03g19170 | -1.991028942 | 0.013749886 | down |
| LOC_Os03g22634 | -1.005405974 | 2.55E-08 | down |
| LOC_Os03g24820 | -4.059000711 | 3.88E-11 | down |
| LOC_Os03g25610 | -1.528415027 | 0.018028737 | down |
| LOC_Os03g26690 | -5.002814807 | 0.012117603 | down |
| LOC_Os03g32040 | -1.299997612 | 0.039453888 | down |
| LOC_Os03g32100 | -2.212344676 | 0.008262929 | down |
| LOC_Os03g36650 | -4.286979381 | 0.009612263 | down |
| LOC_Os03g38534 | -2.677772981 | 0.029620942 | down |
| LOC_Os03g40710 | -1.118080784 | 0.002197517 | down |
| LOC_Os03g40840 | -2.592739795 | 0.005083757 | down |
| LOC_Os03g44260 | -2.92881999 | 0.007712018 | down |
| LOC_Os03g45210 | -2.049630301 | 2.87E-05 | down |
| LOC_Os03g45519 | -4.885255745 | 0.008632941 | down |
| LOC_Os03g45960 | -2.025069632 | 0.002005267 | down |
| LOC_Os03g46040 | -5.474872074 | 0.001678288 | down |
| LOC_Os03g46060 | -1.453268595 | 1.41E-07 | down |
| LOC_Os03g48160 | -1.717875947 | 0.010979832 | down |
| LOC_Os03g51350 | -2.432669663 | 0.007710347 | down |
| LOC_Os03g51530 | -2.148097998 | 3.19E-09 | down |
| LOC_Os03g52680 | -1.115072458 | 1.20E-08 | down |
| LOC_Os03g53260 | -2.762674242 | 0.007990507 | down |
| LOC_Os03g56930 | -1.30285023 | 5.99E-08 | down |
| LOC_Os03g61160 | -2.327412788 | 3.37E-11 | down |
| LOC_Os03g61910 | -2.705377178 | 0.000802322 | down |
| LOC_Os03g62100 | -4.438039426 | 0.02846973 | down |
| LOC_Os04g01470 | -1.501432489 | 0.016811139 | down |
| LOC_Os04g02590 | -6.053230869 | 7.98E-05 | down |
| LOC_Os04g02880 | -1.115114747 | 3.39E-09 | down |
| LOC_Os04g09260 | -1.475136693 | 0.035022981 | down |
| LOC_Os04g09900 | -2.071548007 | 0.017042764 | down |
| LOC_Os04g09920 | -1.698416294 | 0.021917784 | down |
| LOC_Os04g10000 | -2.436243776 | 0.004296561 | down |
| LOC_Os04g10010 | -5.345880766 | 0.00022423 | down |
| LOC_Os04g10160 | -1.920553094 | 0.046264373 | down |
| LOC_Os04g10924 | -1.451968152 | 5.54E-05 | down |
| LOC_Os04g11400 | -1.029689829 | 3.16E-09 | down |
| LOC_Os04g11430 | -2.745145295 | 0.015683204 | down |
| LOC_Os04g14260 | -1.575280874 | 0.004644721 | down |
| LOC_Os04g14280 | -1.932145654 | 0.022241037 | down |
| LOC_Os04g18400 | -12.23242314 | 6.81E-25 | down |
| LOC_Os04g21350 | -1.394281832 | 3.21E-18 | down |
| LOC_Os04g22140 | -1.046346809 | 0.032312285 | down |
| LOC_Os04g22330 | -1.783886786 | 0.001118444 | down |
| LOC_Os04g25800 | -1.5421083 | 0.012690685 | down |
| LOC_Os04g27190 | -1.432426838 | 1.81E-07 | down |
| LOC_Os04g27410 | -2.630767535 | 0.004680089 | down |
| LOC_Os04g30030 | -1.4717611 | 0.017802543 | down |
| LOC_Os04g30240 | -1.276470129 | 5.48E-12 | down |
| LOC_Os04g34320 | -1.018884203 | 2.89E-09 | down |
| LOC_Os04g37970 | -5.97660276 | 1.15E-05 | down |
| LOC_Os04g37980 | -1.221686796 | 2.05E-09 | down |
| LOC_Os04g39370 | -3.199540011 | 0.044596109 | down |
| LOC_Os04g40990 | -2.375070549 | 1.28E-25 | down |
| LOC_Os04g41640 | -1.474726123 | 2.01E-10 | down |
| LOC_Os04g41680 | -1.186865583 | 4.03E-05 | down |
| LOC_Os04g42120 | -2.429951682 | 2.66E-08 | down |
| LOC_Os04g46650 | -1.928302725 | 0.017106169 | down |
| LOC_Os04g48930 | -1.432540092 | 3.73E-15 | down |
| LOC_Os04g49194 | -1.866302163 | 0.034405589 | down |
| LOC_Os04g52250 | -1.047580285 | 2.13E-12 | down |
| LOC_Os04g52810 | -1.325374577 | 2.00E-06 | down |
| LOC_Os04g55790 | -1.056829485 | 0.00597886 | down |
| LOC_Os04g56240 | -1.036242487 | 1.37E-14 | down |
| LOC_Os04g58000 | -4.293256591 | 0.038291862 | down |
| LOC_Os04g59150 | -1.081766694 | 4.45E-11 | down |
| LOC_Os04g59200 | -1.091094081 | 1.20E-05 | down |
| LOC_Os05g02760 | -3.322079627 | 0.019306786 | down |
| LOC_Os05g05550 | -1.909452108 | 1.90E-05 | down |
| LOC_Os05g05650 | -4.346793391 | 0.035021649 | down |
| LOC_Os05g06250 | -1.05466978 | 0.047590597 | down |
| LOC_Os05g06480 | -4.973164341 | 0.012253822 | down |
| LOC_Os05g07560 | -2.432821628 | 1.69E-05 | down |
| LOC_Os05g11310 | -3.949079919 | 0.039999784 | down |
| LOC_Os05g15770 | -1.084859657 | 1.88E-14 | down |
| LOC_Os05g16930 | -1.501684422 | 1.37E-06 | down |
| LOC_Os05g19910 | -1.147790777 | 0.002959497 | down |
| LOC_Os05g20430 | -4.211824102 | 0.018906006 | down |
| LOC_Os05g25370 | -1.582390843 | 4.41E-12 | down |
| LOC_Os05g27580 | -1.443653385 | 0.004972545 | down |
| LOC_Os05g27680 | -4.347047369 | 0.034261025 | down |
| LOC_Os05g28780 | -1.020868032 | 0.034579161 | down |
| LOC_Os05g33220 | -1.187937514 | 0.015212007 | down |
| LOC_Os05g35350 | -4.274371508 | 0.042566487 | down |
| LOC_Os05g35390 | -3.831338051 | 0.033077706 | down |
| LOC_Os05g39140 | -1.339750838 | 4.51E-07 | down |
| LOC_Os05g43390 | -1.180745741 | 0.000601835 | down |
| LOC_Os05g45600 | -3.374243161 | 0.034388798 | down |
| LOC_Os05g46480 | -2.393225725 | 0.000164321 | down |
| LOC_Os05g49240 | -1.373811718 | 1.55E-05 | down |
| LOC_Os05g49440 | -5.109075926 | 0.003460624 | down |
| LOC_Os05g50340 | -1.130367673 | 1.16E-14 | down |
| LOC_Os05g50815 | -2.031251607 | 0.00113813 | down |
| LOC_Os06g01840 | -1.206585201 | 8.59E-07 | down |
| LOC_Os06g02040 | -4.445585152 | 0.026915202 | down |
| LOC_Os06g03670 | -1.283339689 | 0.042302354 | down |
| LOC_Os06g03900 | -1.060419176 | 0.000488499 | down |
| LOC_Os06g04200 | -1.201370863 | 0.000707587 | down |
| LOC_Os06g04990 | -4.56227958 | 0.029076546 | down |
| LOC_Os06g05540 | -1.458323487 | 0.042830057 | down |
| LOC_Os06g06970 | -1.591135696 | 0.014638192 | down |
| LOC_Os06g08310 | -2.184300517 | 0.028726565 | down |
| LOC_Os06g08650 | -1.088870879 | 0.046625861 | down |
| LOC_Os06g10820 | -1.259339419 | 0.022937649 | down |
| LOC_Os06g12370 | -2.561266658 | 0.011477551 | down |
| LOC_Os06g14280 | -1.194707012 | 2.76E-06 | down |
| LOC_Os06g14630 | -2.043897674 | 8.67E-09 | down |
| LOC_Os06g15600 | -3.874796882 | 0.034694402 | down |
| LOC_Os06g20150 | -1.264123236 | 3.12E-08 | down |
| LOC_Os06g20790 | -1.262024966 | 0.003555266 | down |
| LOC_Os06g26270 | -1.253548742 | 0.001996073 | down |
| LOC_Os06g27360 | -1.023077861 | 0.005403962 | down |
| LOC_Os06g28040 | -1.191766577 | 0.034678828 | down |
| LOC_Os06g34840 | -1.584289455 | 0.000276443 | down |
| LOC_Os06g35480 | -4.66099475 | 0.014560054 | down |
| LOC_Os06g35490 | -1.224229138 | 0.016388961 | down |
| LOC_Os06g35700 | -2.275790735 | 0.000149341 | down |
| LOC_Os06g36040 | -1.317470851 | 2.17E-12 | down |
| LOC_Os06g37300 | -1.589770894 | 3.67E-09 | down |
| LOC_Os06g38120 | -1.925318181 | 1.60E-08 | down |
| LOC_Os06g43350 | -1.821331125 | 2.65E-11 | down |
| LOC_Os06g44690 | -4.488323437 | 0.043898436 | down |
| LOC_Os06g46740 | -3.513832268 | 0.000630803 | down |
| LOC_Os06g47270 | -4.367743445 | 0.008248162 | down |
| LOC_Os06g49050 | -1.020410687 | 0.024469693 | down |
| LOC_Os07g03220 | -1.126065777 | 0.005953516 | down |
| LOC_Os07g03710 | -1.367991822 | 0.010495128 | down |
| LOC_Os07g03730 | -3.433121039 | 3.97E-06 | down |
| LOC_Os07g03880 | -1.003687051 | 9.46E-06 | down |
| LOC_Os07g04130 | -1.070917944 | 0.003052137 | down |
| LOC_Os07g05700 | -1.526422994 | 0.001737208 | down |
| LOC_Os07g05840 | -1.263563332 | 0.016284466 | down |
| LOC_Os07g09830 | -2.783636046 | 0.047397686 | down |
| LOC_Os07g09990 | -1.173614277 | 0.020406441 | down |
| LOC_Os07g11739 | -4.512496313 | 0.038857675 | down |
| LOC_Os07g13050 | -2.841714152 | 0.044584863 | down |
| LOC_Os07g13100 | -3.375554869 | 0.002675339 | down |
| LOC_Os07g14660 | -1.72144969 | 0.047043963 | down |
| LOC_Os07g14740 | -2.322373934 | 1.12E-17 | down |
| LOC_Os07g15460 | -1.786706998 | 2.44E-13 | down |
| LOC_Os07g15500 | -1.005613991 | 0.004996324 | down |
| LOC_Os07g16600 | -1.363507543 | 0.005729735 | down |
| LOC_Os07g18970 | -1.11947392 | 0.045265441 | down |
| LOC_Os07g19110 | -4.561700834 | 0.019991866 | down |
| LOC_Os07g22620 | -4.630573035 | 0.030298364 | down |
| LOC_Os07g23944 | -2.45045285 | 2.92E-05 | down |
| LOC_Os07g25050 | -1.904897529 | 2.36E-07 | down |
| LOC_Os07g25530 | -1.248007976 | 0.035061903 | down |
| LOC_Os07g26110 | -1.634216048 | 0.007977703 | down |
| LOC_Os07g29170 | -1.88318803 | 0.044862936 | down |
| LOC_Os07g30280 | -2.379449251 | 0.005270711 | down |
| LOC_Os07g30590 | -4.172893629 | 0.048759422 | down |
| LOC_Os07g31190 | -3.437053226 | 0.005983512 | down |
| LOC_Os07g33898 | -1.567149252 | 0.015083887 | down |
| LOC_Os07g34310 | -1.968234949 | 0.044477425 | down |
| LOC_Os07g34510 | -1.191525111 | 5.45E-10 | down |
| LOC_Os07g34520 | -4.26459777 | 7.85E-47 | down |
| LOC_Os07g34850 | -2.278320629 | 0.02317005 | down |
| LOC_Os07g38130 | -1.67987481 | 0.000224721 | down |
| LOC_Os07g45080 | -2.253926056 | 0.002029315 | down |
| LOC_Os07g46846 | -1.422999726 | 2.59E-06 | down |
| LOC_Os08g01210 | -1.836955426 | 0.046765535 | down |
| LOC_Os08g01950 | -1.842246813 | 0.005479793 | down |
| LOC_Os08g05010 | -1.347183169 | 1.92E-05 | down |
| LOC_Os08g05470 | -1.281409993 | 0.003477642 | down |
| LOC_Os08g05970 | -1.988504737 | 0.042487659 | down |
| LOC_Os08g08690 | -1.560442456 | 0.003803144 | down |
| LOC_Os08g08960 | -1.320163837 | 0.044973073 | down |
| LOC_Os08g14940 | -1.221478973 | 1.20E-13 | down |
| LOC_Os08g15920 | -1.42187051 | 0.001801155 | down |
| LOC_Os08g20486 | -1.637590218 | 0.000177738 | down |
| LOC_Os08g23280 | -2.08500316 | 2.53E-07 | down |
| LOC_Os08g26710 | -1.366875525 | 8.17E-17 | down |
| LOC_Os08g29610 | -4.639370719 | 0.016875445 | down |
| LOC_Os08g29910 | -2.515115283 | 0.034874206 | down |
| LOC_Os08g31950 | -1.50137793 | 0.033834824 | down |
| LOC_Os08g35430 | -4.506492801 | 0.025845938 | down |
| LOC_Os08g41960 | -4.453891765 | 0.025332857 | down |
| LOC_Os08g43390 | -1.124559856 | 1.10E-13 | down |
| LOC_Os08g43810 | -1.008999011 | 0.043616879 | down |
| LOC_Os08g43950 | -1.051726472 | 0.043273227 | down |
| LOC_Os09g04310 | -1.472950868 | 0.001239498 | down |
| LOC_Os09g04360 | -2.303880779 | 0.00405497 | down |
| LOC_Os09g04370 | -2.823916941 | 0.014684824 | down |
| LOC_Os09g13440 | -1.467127658 | 4.59E-06 | down |
| LOC_Os09g16950 | -1.18313434 | 4.41E-10 | down |
| LOC_Os09g17630 | -1.321234247 | 8.54E-07 | down |
| LOC_Os09g18230 | -1.431420304 | 0.00367822 | down |
| LOC_Os09g25200 | -4.506492801 | 0.025845938 | down |
| LOC_Os09g26050 | -1.650862374 | 0.026415369 | down |
| LOC_Os09g26144 | -1.273606145 | 0.000401763 | down |
| LOC_Os09g28500 | -1.248234824 | 0.000898239 | down |
| LOC_Os09g28840 | -2.889588219 | 0.000796433 | down |
| LOC_Os09g29710 | -1.230366179 | 0.000662733 | down |
| LOC_Os09g30414 | -2.539922722 | 0.007030318 | down |
| LOC_Os09g34214 | -1.009435431 | 1.51E-10 | down |
| LOC_Os09g34950 | -3.501916578 | 0.000235412 | down |
| LOC_Os09g35940 | -1.281997963 | 4.57E-20 | down |
| LOC_Os09g39720 | -2.863916692 | 0.043916779 | down |
| LOC_Os10g05250 | -1.030783221 | 0.003752854 | down |
| LOC_Os10g05930 | -2.291374099 | 0.007769862 | down |
| LOC_Os10g06600 | -5.402151567 | 0.001437545 | down |
| LOC_Os10g07160 | -1.270107196 | 0.036585222 | down |
| LOC_Os10g08474 | -1.317916241 | 1.47E-05 | down |
| LOC_Os10g11100 | -5.362307457 | 0.000218892 | down |
| LOC_Os10g11889 | -2.339978878 | 2.66E-24 | down |
| LOC_Os10g17620 | -1.294137732 | 0.028708628 | down |
| LOC_Os10g20440 | -4.514806675 | 0.025389498 | down |
| LOC_Os10g21236 | -2.746919164 | 0.007595345 | down |
| LOC_Os10g21590 | -2.340783824 | 1.07E-05 | down |
| LOC_Os10g22860 | -2.616037352 | 0.022681039 | down |
| LOC_Os10g25040 | -1.190569131 | 2.83E-09 | down |
| LOC_Os10g28080 | -1.50180605 | 4.11E-12 | down |
| LOC_Os10g29260 | -4.781130138 | 0.010504649 | down |
| LOC_Os10g30290 | -1.197955717 | 0.00613514 | down |
| LOC_Os10g33620 | -1.171123597 | 0.017876105 | down |
| LOC_Os10g36070 | -2.228706629 | 0.034947547 | down |
| LOC_Os10g36090 | -4.906188653 | 0.014768883 | down |
| LOC_Os10g39260 | -2.346702158 | 0.011490932 | down |
| LOC_Os10g40040 | -1.791164791 | 0.021680807 | down |
| LOC_Os11g02240 | -1.489601998 | 4.69E-08 | down |
| LOC_Os11g03820 | -1.847700475 | 7.09E-05 | down |
| LOC_Os11g05800 | -1.103553088 | 0.034979356 | down |
| LOC_Os11g06550 | -1.551453189 | 0.011626812 | down |
| LOC_Os11g06640 | -2.670719351 | 0.032889476 | down |
| LOC_Os11g06730 | -1.299131252 | 0.011714462 | down |
| LOC_Os11g07880 | -3.00926347 | 0.023450378 | down |
| LOC_Os11g08830 | -1.746857024 | 0.006905283 | down |
| LOC_Os11g08840 | -1.367401261 | 0.00746977 | down |
| LOC_Os11g14980 | -1.00582099 | 0.04273673 | down |
| LOC_Os11g15040 | -1.319067145 | 1.16E-08 | down |
| LOC_Os11g15170 | -1.834566103 | 2.28E-08 | down |
| LOC_Os11g15340 | -1.018613606 | 0.002667996 | down |
| LOC_Os11g15624 | -2.505824663 | 2.80E-18 | down |
| LOC_Os11g16310 | -1.347450821 | 2.41E-10 | down |
| LOC_Os11g17330 | -1.702612649 | 0.006580593 | down |
| LOC_Os11g18610 | -5.058193264 | 0.004469845 | down |
| LOC_Os11g20320 | -4.600660874 | 0.031573155 | down |
| LOC_Os11g20790 | -1.424579187 | 0.017982851 | down |
| LOC_Os11g26570 | -4.734016899 | 0.013250283 | down |
| LOC_Os11g26780 | -2.765510412 | 0.009498112 | down |
| LOC_Os11g26790 | -4.827793127 | 1.77E-18 | down |
| LOC_Os11g28184 | -2.316811944 | 5.26E-15 | down |
| LOC_Os11g28530 | -1.251285934 | 0.033912558 | down |
| LOC_Os11g32170 | -2.640380843 | 0.038823719 | down |
| LOC_Os11g36000 | -1.803517754 | 0.00165363 | down |
| LOC_Os11g37870 | -1.14696957 | 0.001770932 | down |
| LOC_Os11g37950 | -1.331837616 | 0.004162954 | down |
| LOC_Os11g38580 | -2.393057165 | 4.31E-20 | down |
| LOC_Os11g43520 | -2.312400043 | 0.020409265 | down |
| LOC_Os11g43870 | -1.843564125 | 0.000477016 | down |
| LOC_Os11g45540 | -1.61777262 | 0.018573408 | down |
| LOC_Os11g47140 | -1.169199863 | 7.88E-10 | down |
| LOC_Os12g02840 | -1.143216226 | 0.013355735 | down |
| LOC_Os12g03640 | -2.840348479 | 0.004565711 | down |
| LOC_Os12g03840 | -4.668163635 | 0.013855313 | down |
| LOC_Os12g05470 | -4.839949365 | 0.013827385 | down |
| LOC_Os12g06660 | -3.560807352 | 0.00379629 | down |
| LOC_Os12g07680 | -1.576255668 | 9.32E-14 | down |
| LOC_Os12g08564 | -1.068333991 | 0.010428305 | down |
| LOC_Os12g10440 | -1.017408012 | 0.031275649 | down |
| LOC_Os12g10660 | -1.031372147 | 1.20E-06 | down |
| LOC_Os12g13350 | -2.29991452 | 5.83E-08 | down |
| LOC_Os12g15680 | -2.59127262 | 0.002403905 | down |
| LOC_Os12g16520 | -1.385500749 | 2.45E-05 | down |
| LOC_Os12g16540 | -1.037778457 | 3.32E-08 | down |
| LOC_Os12g21600 | -1.279946985 | 0.039091986 | down |
| LOC_Os12g24020 | -1.696584452 | 6.25E-10 | down |
| LOC_Os12g24320 | -1.318657432 | 5.21E-06 | down |
| LOC_Os12g24490 | -1.939501663 | 0.023918621 | down |
| LOC_Os12g25200 | -1.466255465 | 1.48E-08 | down |
| LOC_Os12g27740 | -9.701523612 | 3.89E-15 | down |
| LOC_Os12g30740 | -4.182592191 | 0.048848127 | down |
| LOC_Os12g31540 | -5.774337222 | 0.000206391 | down |
| LOC_Os12g31860 | -1.406387469 | 0.00018933 | down |
| LOC_Os12g32390 | -1.907467834 | 0.004402445 | down |
| LOC_Os12g32790 | -1.950374404 | 0.005091124 | down |
| LOC_Os12g36850 | -2.979773405 | 8.68E-08 | down |
| LOC_Os12g38051 | -2.102905632 | 0.024473683 | down |
| LOC_Os12g40390 | -1.573599083 | 0.002455795 | down |
| LOC_Os12g40940 | -4.747840285 | 0.011169067 | down |
| LOC_Os12g43450 | -1.172613481 | 0.001386576 | down |
| LOC_Os12g43570 | -1.343019233 | 0.026613212 | down |

**Table S3.** H4K5ac up and downregulated genes in *ossrt2* mutant vs WT.

| **Gene ID** | **Log2Fold change** | ***P* value** | **Expression** |
| --- | --- | --- | --- |
| LOC_Os04g48440 | 9.58 | 1.02E-20 | up |
| LOC_Os06g13680 | 9.3 | 1.39E-19 | up |
| LOC_Os12g16550 | 9.14 | 6.5E-19 | up |
| LOC_Os02g01460 | 8.69 | 3.73E-17 | up |
| LOC_Os11g05070 | 8.24 | 2.05E-15 | up |
| LOC_Os10g25040 | 8.16 | 4.02E-15 | up |
| LOC_Os03g01520 | 8.12 | 5.57E-15 | up |
| LOC_Os03g27400 | 7.97 | 1.9E-14 | up |
| LOC_Os12g02050 | 7.6 | 4.66E-13 | up |
| LOC_Os02g12470 | 7.57 | 1.22E-18 | up |
| LOC_Os03g48890 | 7.44 | 1.56E-12 | up |
| LOC_Os06g23090 | 7.42 | 1.96E-12 | up |
| LOC_Os01g02670 | 7.17 | 1.8E-11 | up |
| LOC_Os10g24004 | 7.17 | 1.42E-11 | up |
| LOC_Os11g02490 | 7.11 | 2.21E-11 | up |
| LOC_Os05g37810 | 6.92 | 1.24E-29 | up |
| LOC_Os05g39920 | 6.69 | 1.52E-18 | up |
| LOC_Os04g14690 | 6.66 | 3.08E-57 | up |
| LOC_Os02g55280 | 6.63 | 3.48E-18 | up |
| LOC_Os07g40986 | 6.47 | 3.11E-09 | up |
| LOC_Os06g08240 | 6.02 | 2.77E-10 | up |
| LOC_Os08g34060 | 5.91 | 1.22E-13 | up |
| LOC_Os02g52730 | 5.7 | 2.19E-27 | up |
| LOC_Os04g56240 | 5.68 | 3.94E-18 | up |
| LOC_Os05g08940 | 5.61 | 2.79E-48 | up |
| LOC_Os12g05680 | 5.45 | 1.43E-16 | up |
| LOC_Os06g19444 | 5.44 | 1E-64 | up |
| LOC_Os12g40730 | 5.31 | 3.62E-56 | up |
| LOC_Os04g21110 | 5.27 | 0.00178 | up |
| LOC_Os11g35300 | 5.25 | 4.07E-24 | up |
| LOC_Os10g41829 | 5.2 | 9.32E-10 | up |
| LOC_Os09g02030 | 5.12 | 1.12E-46 | up |
| LOC_Os12g02140 | 5.12 | 1.11E-12 | up |
| LOC_Os12g12690 | 5.11 | 5.44E-16 | up |
| LOC_Os04g33440 | 5.08 | 6.01E-54 | up |
| LOC_Os02g12240 | 5.02 | 0.000000011 | up |
| LOC_Os02g09080 | 4.99 | 2.31E-10 | up |
| LOC_Os09g09370 | 4.58 | 3.99E-45 | up |
| LOC_Os06g02450 | 4.56 | 0.000248 | up |
| LOC_Os11g41590 | 4.56 | 5.92E-13 | up |
| LOC_Os06g34540 | 4.55 | 0.000672 | up |
| LOC_Os02g51510 | 4.44 | 3.92E-54 | up |
| LOC_Os12g39280 | 4.44 | 1.86E-80 | up |
| LOC_Os09g33620 | 4.43 | 0.000617 | up |
| LOC_Os09g10650 | 4.41 | 0.00379 | up |
| LOC_Os04g05010 | 4.33 | 5.27E-11 | up |
| LOC_Os03g30810 | 4.3 | 6.8E-34 | up |
| LOC_Os09g10660 | 4.26 | 1.17E-57 | up |
| LOC_Os12g05290 | 4.19 | 0.00176 | up |
| LOC_Os07g39630 | 4.16 | 9.15E-12 | up |
| LOC_Os10g09330 | 4.16 | 7.98E-14 | up |
| LOC_Os12g06060 | 4.12 | 6.18E-19 | up |
| LOC_Os04g01910 | 4.07 | 0.00000133 | up |
| LOC_Os06g20740 | 4.03 | 5.44E-26 | up |
| LOC_Os05g45150 | 3.99 | 5.75E-13 | up |
| LOC_Os11g37140 | 3.98 | 2.13E-21 | up |
| LOC_Os10g07050 | 3.92 | 7.17E-60 | up |
| LOC_Os03g19600 | 3.79 | 3.82E-12 | up |
| LOC_Os06g08350 | 3.79 | 2.08E-22 | up |
| LOC_Os01g41020 | 3.76 | 0.00274 | up |
| LOC_Os10g10175 | 3.74 | 6.16E-32 | up |
| LOC_Os05g12100 | 3.67 | 6.76E-21 | up |
| LOC_Os05g43630 | 3.67 | 6.5E-57 | up |
| LOC_Os02g52660 | 3.64 | 0.0327 | up |
| LOC_Os03g02470 | 3.61 | 3.44E-15 | up |
| LOC_Os04g28850 | 3.61 | 5.75E-18 | up |
| LOC_Os05g47920 | 3.61 | 9.98E-19 | up |
| LOC_Os04g34500 | 3.56 | 3.17E-43 | up |
| LOC_Os12g07180 | 3.55 | 1.52E-43 | up |
| LOC_Os05g45170 | 3.48 | 3.34E-75 | up |
| LOC_Os05g45779 | 3.39 | 0.0000674 | up |
| LOC_Os07g46190 | 3.39 | 3.61E-24 | up |
| LOC_Os03g30750 | 3.38 | 2.47E-13 | up |
| LOC_Os05g35970 | 3.34 | 0.0306 | up |
| LOC_Os09g15460 | 3.29 | 8.87E-45 | up |
| LOC_Os06g32330 | 3.24 | 6.69E-49 | up |
| LOC_Os09g16380 | 3.23 | 1.26E-21 | up |
| LOC_Os03g61170 | 3.21 | 3.67E-16 | up |
| LOC_Os01g42420 | 3.2 | 5.77E-47 | up |
| LOC_Os11g09050 | 3.15 | 7.38E-25 | up |
| LOC_Os03g36080 | 3.13 | 1.46E-30 | up |
| LOC_Os06g37630 | 3.11 | 1.48E-16 | up |
| LOC_Os04g31484 | 3.07 | 1.42E-52 | up |
| LOC_Os03g38250 | 3.03 | 4.25E-25 | up |
| LOC_Os01g62540 | 3.02 | 3.36E-24 | up |
| LOC_Os06g40000 | 3.02 | 4.98E-13 | up |
| LOC_Os03g26250 | 3.01 | 1.08E-31 | up |
| LOC_Os12g33194 | 2.98 | 1.36E-12 | up |
| LOC_Os03g58800 | 2.96 | 5.94E-17 | up |
| LOC_Os07g17280 | 2.95 | 2.12E-10 | up |
| LOC_Os02g49510 | 2.9 | 7.32E-17 | up |
| LOC_Os10g41340 | 2.76 | 8.08E-58 | up |
| LOC_Os03g19090 | 2.67 | 2.28E-13 | up |
| LOC_Os08g16700 | 2.67 | 1.07E-32 | up |
| LOC_Os04g28870 | 2.64 | 1.62E-12 | up |
| LOC_Os05g23050 | 2.64 | 1.14E-62 | up |
| LOC_Os07g38200 | 2.63 | 8.71E-14 | up |
| LOC_Os05g24610 | 2.62 | 4.26E-59 | up |
| LOC_Os01g15520 | 2.58 | 4.46E-39 | up |
| LOC_Os04g55140 | 2.55 | 1.26E-09 | up |
| LOC_Os12g35910 | 2.53 | 1.1E-11 | up |
| LOC_Os02g01220 | 2.51 | 1.02E-11 | up |
| LOC_Os07g05450 | 2.46 | 2.07E-53 | up |
| LOC_Os12g36750 | 2.46 | 0.00000314 | up |
| LOC_Os01g14830 | 2.45 | 3.11E-52 | up |
| LOC_Os06g49350 | 2.45 | 1.63E-15 | up |
| LOC_Os08g31730 | 2.45 | 1.88E-31 | up |
| LOC_Os07g10850 | 2.44 | 2.87E-26 | up |
| LOC_Os03g12200 | 2.41 | 1.24E-21 | up |
| LOC_Os02g02330 | 2.4 | 7.48E-13 | up |
| LOC_Os09g02050 | 2.38 | 0.0000573 | up |
| LOC_Os01g15090 | 2.36 | 2.18E-15 | up |
| LOC_Os05g23250 | 2.29 | 2.27E-34 | up |
| LOC_Os04g52790 | 2.27 | 1.04E-19 | up |
| LOC_Os05g45120 | 2.26 | 1.44E-10 | up |
| LOC_Os01g55400 | 2.22 | 9.12E-14 | up |
| LOC_Os11g47740 | 2.22 | 1.15E-11 | up |
| LOC_Os01g49740 | 2.21 | 6.79E-50 | up |
| LOC_Os03g45000 | 2.21 | 2.04E-10 | up |
| LOC_Os04g38540 | 2.2 | 3.04E-12 | up |
| LOC_Os06g14440 | 2.19 | 1.02E-20 | up |
| LOC_Os06g14870 | 2.19 | 5.02E-24 | up |
| LOC_Os05g06814 | 2.17 | 7.57E-13 | up |
| LOC_Os12g26470 | 2.15 | 3.51E-11 | up |
| LOC_Os02g52010 | 2.12 | 5.5E-16 | up |
| LOC_Os03g63930 | 2.06 | 0.00000059 | up |
| LOC_Os07g30940 | 2.04 | 4.24E-37 | up |
| LOC_Os05g44930 | 2.02 | 6.3E-42 | up |
| LOC_Os06g21369 | 2.02 | 1.9E-39 | up |
| LOC_Os02g49910 | 2.01 | 6.04E-15 | up |
| LOC_Os06g33200 | 2.01 | 4.68E-14 | up |
| LOC_Os03g20890 | 2 | 5.07E-52 | up |
| LOC_Os04g32090 | 2 | 4.66E-51 | up |
| LOC_Os04g39864 | 1.96 | 3.53E-22 | up |
| LOC_Os05g07170 | 1.96 | 6.87E-46 | up |
| LOC_Os06g22360 | 1.95 | 1.4E-20 | up |
| LOC_Os07g02030 | 1.95 | 2.69E-11 | up |
| LOC_Os03g62224 | 1.93 | 5.58E-16 | up |
| LOC_Os05g34490 | 1.93 | 8.83E-40 | up |
| LOC_Os04g42010 | 1.91 | 1.68E-27 | up |
| LOC_Os07g08940 | 1.91 | 3.11E-20 | up |
| LOC_Os02g50404 | 1.9 | 3.08E-17 | up |
| LOC_Os06g40240 | 1.9 | 3.1E-35 | up |
| LOC_Os08g27678 | 1.9 | 2.4E-21 | up |
| LOC_Os08g43180 | 1.89 | 0.000000985 | up |
| LOC_Os03g35326 | 1.87 | 4.16E-18 | up |
| LOC_Os01g61830 | 1.84 | 0.000000116 | up |
| LOC_Os03g41339 | 1.83 | 7.11E-36 | up |
| LOC_Os06g09130 | 1.83 | 6.13E-10 | up |
| LOC_Os02g12510 | 1.82 | 2.69E-20 | up |
| LOC_Os02g13620 | 1.82 | 0.000000902 | up |
| LOC_Os05g10320 | 1.81 | 1.62E-09 | up |
| LOC_Os02g53230 | 1.8 | 2.91E-52 | up |
| LOC_Os02g52120 | 1.78 | 3.17E-16 | up |
| LOC_Os05g50390 | 1.69 | 1.68E-52 | up |
| LOC_Os06g15730 | 1.69 | 1.54E-19 | up |
| LOC_Os05g07080 | 1.61 | 0.00000863 | up |
| LOC_Os03g62120 | 1.59 | 0.00000244 | up |
| LOC_Os05g38700 | 1.59 | 6.19E-21 | up |
| LOC_Os08g09990 | 1.59 | 8.43E-25 | up |
| LOC_Os05g51820 | 1.56 | 5.98E-11 | up |
| LOC_Os11g08569 | 1.56 | 1.43E-30 | up |
| LOC_Os09g14610 | 1.55 | 5.95E-19 | up |
| LOC_Os09g06730 | 1.54 | 5.26E-11 | up |
| LOC_Os12g02170 | 1.54 | 7.42E-08 | up |
| LOC_Os05g04510 | 1.53 | 0.0000689 | up |
| LOC_Os11g25980 | 1.52 | 1.91E-16 | up |
| LOC_Os02g15430 | 1.51 | 2.03E-09 | up |
| LOC_Os05g44560 | 1.51 | 0.0000159 | up |
| LOC_Os07g41080 | 1.5 | 1.26E-08 | up |
| LOC_Os03g27200 | 1.49 | 3.8E-10 | up |
| LOC_Os04g16340 | 1.49 | 0.0000129 | up |
| LOC_Os04g27310 | 1.49 | 5.78E-20 | up |
| LOC_Os02g52770 | 1.48 | 0.0000679 | up |
| LOC_Os03g18779 | 1.48 | 6.89E-44 | up |
| LOC_Os03g55460 | 1.48 | 1.13E-10 | up |
| LOC_Os05g42420 | 1.48 | 1.14E-09 | up |
| LOC_Os09g38104 | 1.47 | 7.47E-37 | up |
| LOC_Os08g43010 | 1.46 | 0.000000106 | up |
| LOC_Os01g11620 | 1.45 | 2.38E-14 | up |
| LOC_Os03g08350 | 1.45 | 4.42E-15 | up |
| LOC_Os12g03480 | 1.45 | 1.41E-29 | up |
| LOC_Os02g27550 | 1.44 | 0.000000809 | up |
| LOC_Os11g02220 | 1.44 | 1.24E-13 | up |
| LOC_Os08g10612 | 1.43 | 0.000000862 | up |
| LOC_Os11g34640 | 1.42 | 0.0000463 | up |
| LOC_Os05g14730 | 1.41 | 0.00000852 | up |
| LOC_Os09g09540 | 1.4 | 0.00000162 | up |
| LOC_Os02g21320 | 1.39 | 1.54E-13 | up |
| LOC_Os02g14480 | 1.38 | 0.000000235 | up |
| LOC_Os10g29810 | 1.38 | 1.42E-09 | up |
| LOC_Os03g43100 | 1.37 | 3.87E-34 | up |
| LOC_Os02g36860 | 1.33 | 9.15E-15 | up |
| LOC_Os08g01590 | 1.33 | 1.56E-08 | up |
| LOC_Os03g40070 | 1.3 | 0.000117 | up |
| LOC_Os05g46690 | 1.3 | 0.0000217 | up |
| LOC_Os07g02620 | 1.29 | 2.03E-12 | up |
| LOC_Os03g02230 | 1.27 | 4.92E-15 | up |
| LOC_Os05g39860 | 1.27 | 0.00000993 | up |
| LOC_Os06g07420 | 1.27 | 3.8E-22 | up |
| LOC_Os10g33770 | 1.26 | 0.00129 | up |
| LOC_Os02g31850 | 1.25 | 8.36E-09 | up |
| LOC_Os03g53780 | 1.24 | 0.0000332 | up |
| LOC_Os10g35170 | 1.22 | 4.2E-18 | up |
| LOC_Os03g51270 | 1.21 | 5.81E-11 | up |
| LOC_Os08g07700 | 1.21 | 0.000591 | up |
| LOC_Os06g35200 | 1.19 | 0.00155 | up |
| LOC_Os06g48980 | 1.19 | 0.000000303 | up |
| LOC_Os03g42270 | 1.18 | 0.00000718 | up |
| LOC_Os05g24160 | 1.18 | 2.92E-09 | up |
| LOC_Os06g39110 | 1.17 | 0.0000181 | up |
| LOC_Os09g07170 | 1.17 | 0.00228 | up |
| LOC_Os02g04880 | 1.16 | 0.0000899 | up |
| LOC_Os02g57860 | 1.16 | 3.8E-14 | up |
| LOC_Os09g25980 | 1.15 | 0.000113 | up |
| LOC_Os01g32660 | 1.14 | 0.000124 | up |
| LOC_Os02g57960 | 1.14 | 0.0000088 | up |
| LOC_Os10g34602 | 1.14 | 6.19E-40 | up |
| LOC_Os01g70850 | 1.12 | 0.000151 | up |
| LOC_Os12g40920 | 1.12 | 0.000000172 | up |
| LOC_Os12g08040 | 1.1 | 2.58E-18 | up |
| LOC_Os09g36220 | 1.09 | 0.000286 | up |
| LOC_Os02g58310 | 1.08 | 0.00000104 | up |
| LOC_Os04g12660 | 1.08 | 0.0000382 | up |
| LOC_Os01g04830 | 1.07 | 0.000377 | up |
| LOC_Os02g32814 | 1.07 | 5.42E-09 | up |
| LOC_Os07g01580 | 1.06 | 0.0021 | up |
| LOC_Os06g46610 | 1.05 | 0.000000217 | up |
| LOC_Os10g07546 | 1.03 | 0.000000549 | up |
| LOC_Os11g05690 | 1.03 | 0.000793 | up |
| LOC_Os12g35440 | 1.03 | 0.000274 | up |
| LOC_Os01g33030 | 1.01 | 0.000805 | up |
| LOC_Os03g57910 | 1.01 | 0.000011 | up |
| LOC_Os09g25300 | 1.01 | 0.000000287 | up |
| LOC_Os12g39380 | 1.01 | 0.00000136 | up |
| LOC_Os01g65094 | 1 | 0.000637 | up |
| LOC_Os10g39140 | 0.99 | 0.00138 | up |
| LOC_Os03g22130 | 0.98 | 0.000924 | up |
| LOC_Os10g39190 | 0.98 | 3.08E-18 | up |
| LOC_Os11g29030 | 0.98 | 5.66E-14 | up |
| LOC_Os02g21120 | 0.97 | 3.54E-19 | up |
| LOC_Os06g11140 | 0.97 | 0.000403 | up |
| LOC_Os09g28489 | 0.96 | 0.00188 | up |
| LOC_Os02g15160 | 0.95 | 0.000178 | up |
| LOC_Os04g25100 | 0.95 | 2.23E-28 | up |
| LOC_Os06g48600 | 0.95 | 0.00259 | up |
| LOC_Os02g09340 | 0.93 | 0.000239 | up |
| LOC_Os05g24530 | 0.93 | 6.42E-13 | up |
| LOC_Os09g39034 | 0.93 | 0.000018 | up |
| LOC_Os10g07460 | 0.93 | 0.000488 | up |
| LOC_Os03g06160 | 0.92 | 0.0000851 | up |
| LOC_Os09g25330 | 0.92 | 0.00314 | up |
| LOC_Os04g27340 | 0.9 | 0.00887 | up |
| LOC_Os05g03750 | 0.9 | 0.00029 | up |
| LOC_Os06g47460 | 0.9 | 0.000000122 | up |
| LOC_Os07g46080 | 0.9 | 0.00013 | up |
| LOC_Os09g28790 | 0.9 | 0.0052 | up |
| LOC_Os11g37970 | 0.9 | 0.00284 | up |
| LOC_Os01g36860 | 0.89 | 0.00298 | up |
| LOC_Os04g39350 | 0.89 | 0.000203 | up |
| LOC_Os07g35960 | 0.89 | 0.000301 | up |
| LOC_Os03g20720 | 0.87 | 0.00629 | up |
| LOC_Os05g10930 | 0.87 | 0.000294 | up |
| LOC_Os06g42890 | 0.87 | 4.87E-08 | up |
| LOC_Os04g32540 | 0.86 | 0.00685 | up |
| LOC_Os07g01904 | 0.86 | 1.16E-08 | up |
| LOC_Os07g46070 | 0.86 | 3.44E-11 | up |
| LOC_Os08g07320 | 0.86 | 0.00164 | up |
| LOC_Os01g18240 | 0.85 | 0.00314 | up |
| LOC_Os04g57130 | 0.85 | 4.85E-10 | up |
| LOC_Os01g71940 | 0.84 | 0.0000685 | up |
| LOC_Os05g39130 | 0.84 | 0.000000322 | up |
| LOC_Os05g46720 | 0.84 | 0.00000104 | up |
| LOC_Os10g04520 | 0.84 | 0.000000531 | up |
| LOC_Os11g03890 | 0.84 | 0.0252 | up |
| LOC_Os03g45330 | 0.83 | 0.00148 | up |
| LOC_Os09g33500 | 0.83 | 0.0000425 | up |
| LOC_Os02g47990 | 0.82 | 0.000708 | up |
| LOC_Os07g48450 | 0.82 | 0.00135 | up |
| LOC_Os09g18470 | 0.82 | 1.36E-08 | up |
| LOC_Os12g14764 | 0.82 | 0.000663 | up |
| LOC_Os04g38270 | 0.81 | 3.53E-13 | up |
| LOC_Os04g51370 | 0.8 | 0.000375 | up |
| LOC_Os04g52180 | 0.8 | 0.00258 | up |
| LOC_Os03g30890 | 0.79 | 0.0000131 | up |
| LOC_Os06g33350 | 0.79 | 0.0032 | up |
| LOC_Os06g49410 | 0.79 | 0.00000111 | up |
| LOC_Os09g11320 | 0.79 | 0.000244 | up |
| LOC_Os10g41980 | 0.79 | 0.0000203 | up |
| LOC_Os01g15600 | 0.78 | 0.00926 | up |
| LOC_Os02g01100 | 0.78 | 0.019 | up |
| LOC_Os06g05380 | 0.78 | 0.000000254 | up |
| LOC_Os09g25760 | 0.78 | 0.00000406 | up |
| LOC_Os09g33860 | 0.78 | 0.00794 | up |
| LOC_Os02g09930 | 0.77 | 0.0087 | up |
| LOC_Os04g17660 | 0.77 | 2.01E-16 | up |
| LOC_Os10g36270 | 0.77 | 0.00479 | up |
| LOC_Os01g46760 | 0.76 | 0.007 | up |
| LOC_Os02g21190 | 0.76 | 0.00123 | up |
| LOC_Os05g04770 | 0.76 | 0.0132 | up |
| LOC_Os08g05750 | 0.76 | 0.000000131 | up |
| LOC_Os10g22410 | 0.76 | 0.000724 | up |
| LOC_Os10g31330 | 0.76 | 0.00125 | up |
| LOC_Os01g62680 | 0.75 | 0.0113 | up |
| LOC_Os01g67134 | 0.75 | 7.09E-10 | up |
| LOC_Os03g37520 | 0.75 | 0.00106 | up |
| LOC_Os03g38740 | 0.75 | 0.00641 | up |
| LOC_Os05g07430 | 0.75 | 0.00542 | up |
| LOC_Os08g02540 | 0.75 | 0.0275 | up |
| LOC_Os08g11150 | 0.75 | 0.00748 | up |
| LOC_Os10g39044 | 0.75 | 0.00127 | up |
| LOC_Os11g24150 | 0.75 | 0.00000125 | up |
| LOC_Os12g35580 | 0.75 | 0.0102 | up |
| LOC_Os01g12710 | 0.74 | 0.00594 | up |
| LOC_Os01g41960 | 0.74 | 3.71E-21 | up |
| LOC_Os02g18410 | 0.74 | 0.0252 | up |
| LOC_Os04g36780 | 0.74 | 0.0194 | up |
| LOC_Os06g38220 | 0.74 | 0.000355 | up |
| LOC_Os07g42560 | 0.74 | 3.53E-10 | up |
| LOC_Os09g07810 | 0.74 | 0.0112 | up |
| LOC_Os10g21400 | 0.74 | 0.0011 | up |
| LOC_Os01g59819 | 0.73 | 0.00412 | up |
| LOC_Os03g53510 | 0.73 | 0.0119 | up |
| LOC_Os05g14270 | 0.73 | 0.00258 | up |
| LOC_Os11g16310 | 0.73 | 0.00223 | up |
| LOC_Os12g36830 | 0.73 | 0.0000261 | up |
| LOC_Os03g52239 | 0.72 | 0.00384 | up |
| LOC_Os03g64080 | 0.72 | 0.0143 | up |
| LOC_Os04g49954 | 0.72 | 0.00136 | up |
| LOC_Os05g31680 | 0.72 | 0.0149 | up |
| LOC_Os06g13860 | 0.72 | 0.00446 | up |
| LOC_Os09g09830 | 0.72 | 0.0134 | up |
| LOC_Os09g10180 | 0.72 | 0.017 | up |
| LOC_Os10g30150 | 0.72 | 1E-15 | up |
| LOC_Os11g32220 | 0.72 | 0.00387 | up |
| LOC_Os11g36160 | 0.72 | 0.00658 | up |
| LOC_Os12g35310 | 0.72 | 5.23E-08 | up |
| LOC_Os12g43970 | 0.72 | 3.54E-22 | up |
| LOC_Os02g05980 | 0.71 | 0.000848 | up |
| LOC_Os03g10290 | 0.71 | 7.35E-20 | up |
| LOC_Os01g33810 | 0.7 | 0.000512 | up |
| LOC_Os01g43700 | 0.7 | 5.41E-15 | up |
| LOC_Os02g36590 | 0.7 | 0.0000257 | up |
| LOC_Os03g14820 | 0.7 | 4.44E-09 | up |
| LOC_Os05g06920 | 0.7 | 0.0215 | up |
| LOC_Os06g06630 | 0.7 | 0.000638 | up |
| LOC_Os06g23970 | 0.7 | 0.0101 | up |
| LOC_Os07g34980 | 0.7 | 0.00206 | up |
| LOC_Os08g40630 | 0.7 | 0.00205 | up |
| LOC_Os11g34460 | 0.7 | 0.0000519 | up |
| LOC_Os01g27230 | 0.69 | 0.0000174 | up |
| LOC_Os01g37870 | 0.69 | 0.0033 | up |
| LOC_Os07g07654 | 0.69 | 0.000839 | up |
| LOC_Os08g33370 | 0.69 | 0.000272 | up |
| LOC_Os02g11980 | 0.68 | 0.000316 | up |
| LOC_Os02g13140 | 0.68 | 0.0289 | up |
| LOC_Os04g54430 | 0.68 | 0.00307 | up |
| LOC_Os05g05660 | 0.68 | 0.0000194 | up |
| LOC_Os08g42630 | 0.68 | 0.000000378 | up |
| LOC_Os08g44560 | 0.68 | 0.000000287 | up |
| LOC_Os02g55430 | 0.67 | 0.0123 | up |
| LOC_Os03g05610 | 0.67 | 0.0000871 | up |
| LOC_Os04g27810 | 0.67 | 0.0208 | up |
| LOC_Os05g13390 | 0.67 | 0.00949 | up |
| LOC_Os06g42630 | 0.67 | 0.00179 | up |
| LOC_Os08g07860 | 0.67 | 0.000000123 | up |
| LOC_Os01g12540 | 0.66 | 0.0232 | up |
| LOC_Os03g11440 | 0.66 | 0.0415 | up |
| LOC_Os04g29840 | 0.66 | 0.000916 | up |
| LOC_Os07g43940 | 0.66 | 0.000478 | up |
| LOC_Os08g39630 | 0.66 | 0.0221 | up |
| LOC_Os01g43390 | 0.65 | 0.00531 | up |
| LOC_Os01g47840 | 0.65 | 0.00285 | up |
| LOC_Os01g60230 | 0.65 | 0.025 | up |
| LOC_Os01g70290 | 0.65 | 0.0305 | up |
| LOC_Os02g43000 | 0.65 | 0.000982 | up |
| LOC_Os04g40720 | 0.65 | 1.54E-13 | up |
| LOC_Os06g30750 | 0.65 | 0.0193 | up |
| LOC_Os07g05210 | 0.65 | 0.0129 | up |
| LOC_Os07g25730 | 0.65 | 0.0017 | up |
| LOC_Os08g28590 | 0.65 | 0.000735 | up |
| LOC_Os10g04110 | 0.65 | 0.00000295 | up |
| LOC_Os12g07680 | 0.65 | 2.07E-11 | up |
| LOC_Os03g03610 | 0.64 | 0.000464 | up |
| LOC_Os03g47120 | 0.64 | 0.0141 | up |
| LOC_Os04g10050 | 0.64 | 0.00158 | up |
| LOC_Os05g19680 | 0.64 | 0.00253 | up |
| LOC_Os06g40200 | 0.64 | 0.00115 | up |
| LOC_Os06g43650 | 0.64 | 0.0152 | up |
| LOC_Os08g33400 | 0.64 | 0.000269 | up |
| LOC_Os09g29430 | 0.64 | 0.00368 | up |
| LOC_Os12g02830 | 0.64 | 0.00085 | up |
| LOC_Os01g40700 | 0.63 | 0.017 | up |
| LOC_Os01g74430 | 0.63 | 0.0394 | up |
| LOC_Os02g15660 | 0.63 | 0.00116 | up |
| LOC_Os04g45860 | 0.63 | 0.00242 | up |
| LOC_Os04g55660 | 0.63 | 0.000363 | up |
| LOC_Os08g26875 | 0.63 | 0.00308 | up |
| LOC_Os10g22060 | 0.63 | 0.0146 | up |
| LOC_Os10g39560 | 0.63 | 0.0167 | up |
| LOC_Os11g06720 | 0.63 | 0.00982 | up |
| LOC_Os01g05070 | 0.62 | 0.0194 | up |
| LOC_Os01g64262 | 0.62 | 0.0193 | up |
| LOC_Os02g15640 | 0.62 | 0.0386 | up |
| LOC_Os04g44930 | 0.62 | 0.00228 | up |
| LOC_Os06g47970 | 0.62 | 0.0379 | up |
| LOC_Os07g05820 | 0.62 | 0.000903 | up |
| LOC_Os07g13770 | 0.62 | 8.51E-09 | up |
| LOC_Os07g25900 | 0.62 | 0.00531 | up |
| LOC_Os07g48510 | 0.62 | 0.0401 | up |
| LOC_Os01g24480 | 0.61 | 0.000922 | up |
| LOC_Os03g07400 | 0.61 | 0.000113 | up |
| LOC_Os03g08570 | 0.61 | 0.000162 | up |
| LOC_Os04g30490 | 0.61 | 0.000408 | up |
| LOC_Os05g14240 | 0.61 | 0.0371 | up |
| LOC_Os06g02480 | 0.61 | 0.00285 | up |
| LOC_Os06g12990 | 0.61 | 0.0381 | up |
| LOC_Os07g38970 | 0.61 | 0.00198 | up |
| LOC_Os08g07730 | 0.61 | 0.0407 | up |
| LOC_Os12g32499 | 0.61 | 0.00000415 | up |
| LOC_Os12g42850 | 0.61 | 0.00824 | up |
| LOC_Os02g13510 | 0.6 | 0.00129 | up |
| LOC_Os02g24210 | 0.6 | 2.54E-10 | up |
| LOC_Os02g39160 | 0.6 | 0.0426 | up |
| LOC_Os02g43350 | 0.6 | 0.0328 | up |
| LOC_Os02g55900 | 0.6 | 0.00085 | up |
| LOC_Os02g56550 | 0.6 | 0.0276 | up |
| LOC_Os03g06290 | 0.6 | 0.023 | up |
| LOC_Os03g27980 | 0.6 | 0.0135 | up |
| LOC_Os04g57760 | 0.6 | 0.0394 | up |
| LOC_Os05g04610 | 0.6 | 0.0126 | up |
| LOC_Os06g51260 | 0.6 | 0.014 | up |
| LOC_Os07g42570 | 0.6 | 0.00487 | up |
| LOC_Os09g37540 | 0.6 | 0.00531 | up |
| LOC_Os11g39650 | 0.6 | 0.000383 | up |
| LOC_Os01g04005 | 0.59 | 0.0012 | up |
| LOC_Os01g11650 | 0.59 | 0.0277 | up |
| LOC_Os04g21890 | 0.59 | 0.0152 | up |
| LOC_Os04g49120 | 0.59 | 0.0205 | up |
| LOC_Os05g02120 | 0.59 | 0.0292 | up |
| LOC_Os07g18240 | 0.59 | 0.0256 | up |
| LOC_Os07g40260 | 0.59 | 0.00538 | up |
| LOC_Os07g46130 | 0.59 | 0.0184 | up |
| LOC_Os08g33740 | 0.59 | 0.0393 | up |
| LOC_Os08g38800 | 0.59 | 5.39E-14 | up |
| LOC_Os08g39250 | 0.59 | 0.0361 | up |
| LOC_Os09g02729 | 0.59 | 0.000447 | up |
| LOC_Os09g13540 | 0.59 | 5.83E-08 | up |
| LOC_Os01g60700 | 0.58 | 0.0374 | up |
| LOC_Os01g70210 | 0.58 | 0.0142 | up |
| LOC_Os02g13555 | 0.58 | 0.00913 | up |
| LOC_Os05g05600 | 0.58 | 0.0145 | up |
| LOC_Os06g14500 | 0.58 | 0.00168 | up |
| LOC_Os08g04180 | 0.58 | 0.000158 | up |
| LOC_Os09g33470 | 0.58 | 0.00000783 | up |
| LOC_Os11g06200 | 0.58 | 0.00712 | up |
| LOC_Os11g10250 | 0.58 | 0.00000151 | up |
| LOC_Os12g08810 | 0.58 | 0.000408 | up |
| LOC_Os01g02000 | -0.83 | 0.00143 | down |
| LOC_Os01g06790 | -0.7 | 0.0126 | down |
| LOC_Os01g08650 | -0.64 | 0.0205 | down |
| LOC_Os01g09450 | -0.63 | 0.0487 | down |
| LOC_Os01g09930 | -1.15 | 0.00101 | down |
| LOC_Os01g11730 | -0.64 | 0.0335 | down |
| LOC_Os01g15709 | -0.66 | 0.000658 | down |
| LOC_Os01g23705 | -0.77 | 0.0153 | down |
| LOC_Os01g25510 | -0.93 | 0.00755 | down |
| LOC_Os01g26390 | -0.64 | 0.0388 | down |
| LOC_Os01g32864 | -0.7 | 0.00804 | down |
| LOC_Os01g34010 | -0.69 | 0.0115 | down |
| LOC_Os01g36810 | -0.65 | 0.0245 | down |
| LOC_Os01g38760 | -0.67 | 0.000532 | down |
| LOC_Os01g41900 | -0.82 | 0.00762 | down |
| LOC_Os01g45160 | -0.7 | 0.00397 | down |
| LOC_Os01g45659 | -0.66 | 0.00274 | down |
| LOC_Os01g53280 | -0.59 | 0.0355 | down |
| LOC_Os01g59680 | -0.82 | 0.000236 | down |
| LOC_Os01g61350 | -0.85 | 0.00221 | down |
| LOC_Os01g62600 | -0.71 | 0.0443 | down |
| LOC_Os01g67970 | -0.58 | 0.00337 | down |
| LOC_Os01g67980 | -0.85 | 0.00249 | down |
| LOC_Os01g72150 | -0.71 | 0.00517 | down |
| LOC_Os01g72630 | -0.69 | 3.02E-05 | down |
| LOC_Os01g73710 | -0.71 | 0.00206 | down |
| LOC_Os01g74152 | -0.81 | 0.0155 | down |
| LOC_Os02g02190 | -0.9 | 6.66E-05 | down |
| LOC_Os02g02250 | -0.58 | 0.0326 | down |
| LOC_Os02g03310 | -0.71 | 0.0211 | down |
| LOC_Os02g03660 | -0.71 | 0.00745 | down |
| LOC_Os02g03900 | -0.64 | 0.0322 | down |
| LOC_Os02g04990 | -0.79 | 0.000223 | down |
| LOC_Os02g05040 | -0.64 | 0.0114 | down |
| LOC_Os02g05080 | -0.58 | 0.000428 | down |
| LOC_Os02g06100 | -0.71 | 0.0053 | down |
| LOC_Os02g06600 | -0.6 | 0.0486 | down |
| LOC_Os02g06630 | -0.87 | 0.000216 | down |
| LOC_Os02g07330 | -0.63 | 0.0489 | down |
| LOC_Os02g11010 | -0.67 | 0.00211 | down |
| LOC_Os02g12280 | -0.61 | 0.0196 | down |
| LOC_Os02g13930 | -0.86 | 0.00873 | down |
| LOC_Os02g17060 | -0.92 | 0.0061 | down |
| LOC_Os02g17200 | -1.21 | 1.74E-05 | down |
| LOC_Os02g18140 | -0.67 | 0.00985 | down |
| LOC_Os02g21100 | -0.83 | 0.00565 | down |
| LOC_Os02g25830 | -0.74 | 5.49E-05 | down |
| LOC_Os02g26320 | -0.59 | 0.0249 | down |
| LOC_Os02g26770 | -0.83 | 0.000205 | down |
| LOC_Os02g29630 | -0.77 | 0.0114 | down |
| LOC_Os02g36700 | -0.58 | 0.0314 | down |
| LOC_Os02g39090 | -0.6 | 0.0188 | down |
| LOC_Os02g42110 | -0.83 | 0.0112 | down |
| LOC_Os02g48094 | -0.6 | 0.00345 | down |
| LOC_Os02g49740 | -0.6 | 0.000288 | down |
| LOC_Os02g52800 | -0.59 | 0.00563 | down |
| LOC_Os02g57090 | -0.58 | 0.0356 | down |
| LOC_Os02g58390 | -0.67 | 0.000295 | down |
| LOC_Os03g01700 | -0.69 | 0.00514 | down |
| LOC_Os03g04240 | -0.65 | 0.000691 | down |
| LOC_Os03g05080 | -0.78 | 2.15E-07 | down |
| LOC_Os03g14654 | -0.68 | 0.00481 | down |
| LOC_Os03g16780 | -0.68 | 0.0142 | down |
| LOC_Os03g17510 | -0.76 | 0.0145 | down |
| LOC_Os03g19600 | -0.81 | 0.00207 | down |
| LOC_Os03g24650 | -0.63 | 0.00906 | down |
| LOC_Os03g25010 | -0.61 | 0.00239 | down |
| LOC_Os03g26250 | -0.87 | 3.23E-06 | down |
| LOC_Os03g26870 | -0.6 | 0.0203 | down |
| LOC_Os03g28990 | -0.66 | 0.0153 | down |
| LOC_Os03g36400 | -0.58 | 0.00117 | down |
| LOC_Os03g36560 | -0.58 | 0.0181 | down |
| LOC_Os03g36920 | -0.63 | 0.0229 | down |
| LOC_Os03g40130 | -0.64 | 0.00236 | down |
| LOC_Os03g40540 | -0.81 | 0.0118 | down |
| LOC_Os03g41339 | -0.64 | 0.0189 | down |
| LOC_Os03g42760 | -0.63 | 0.00106 | down |
| LOC_Os03g43140 | -0.65 | 0.0454 | down |
| LOC_Os03g47470 | -0.6 | 0.00858 | down |
| LOC_Os03g47830 | -0.75 | 0.000118 | down |
| LOC_Os03g48810 | -0.81 | 0.00752 | down |
| LOC_Os03g55220 | -0.69 | 0.0401 | down |
| LOC_Os03g57620 | -0.64 | 0.0173 | down |
| LOC_Os03g57630 | -0.95 | 1.07E-08 | down |
| LOC_Os04g01890 | -0.86 | 0.00642 | down |
| LOC_Os04g04950 | -0.8 | 0.00426 | down |
| LOC_Os04g09620 | -0.79 | 0.0174 | down |
| LOC_Os04g13190 | -0.6 | 0.0373 | down |
| LOC_Os04g18400 | -9.56 | 1.27E-20 | down |
| LOC_Os04g24410 | -0.63 | 0.0154 | down |
| LOC_Os04g28805 | -0.69 | 0.0118 | down |
| LOC_Os04g29260 | -0.67 | 0.0134 | down |
| LOC_Os04g34500 | -0.68 | 0.00258 | down |
| LOC_Os04g35430 | -0.64 | 0.0311 | down |
| LOC_Os04g36040 | -0.62 | 0.0108 | down |
| LOC_Os04g36720 | -0.58 | 0.00157 | down |
| LOC_Os04g37440 | -0.59 | 0.0223 | down |
| LOC_Os04g38540 | -0.59 | 0.000522 | down |
| LOC_Os04g42120 | -1.13 | 6.06E-18 | down |
| LOC_Os04g47400 | -0.67 | 0.00185 | down |
| LOC_Os04g49600 | -0.58 | 8.29E-06 | down |
| LOC_Os04g50770 | -0.58 | 0.0481 | down |
| LOC_Os04g51980 | -0.58 | 0.00323 | down |
| LOC_Os04g53360 | -0.66 | 0.0466 | down |
| LOC_Os04g54810 | -0.62 | 0.0133 | down |
| LOC_Os04g55770 | -0.63 | 0.00192 | down |
| LOC_Os04g56170 | -0.68 | 0.00909 | down |
| LOC_Os05g01790 | -0.75 | 0.00397 | down |
| LOC_Os05g04890 | -0.74 | 0.0142 | down |
| LOC_Os05g05370 | -0.81 | 0.00634 | down |
| LOC_Os05g06080 | -0.6 | 0.0275 | down |
| LOC_Os05g06130 | -0.63 | 0.0323 | down |
| LOC_Os05g13330 | -0.65 | 0.025 | down |
| LOC_Os05g13620 | -0.59 | 0.018 | down |
| LOC_Os05g15240 | -0.62 | 0.00329 | down |
| LOC_Os05g16170 | -0.61 | 0.00379 | down |
| LOC_Os05g16900 | -0.63 | 0.0303 | down |
| LOC_Os05g18294 | -0.78 | 0.011 | down |
| LOC_Os05g30070 | -3.06 | 4.01E-23 | down |
| LOC_Os05g30300 | -0.61 | 0.0154 | down |
| LOC_Os05g30660 | -0.66 | 0.00293 | down |
| LOC_Os05g32880 | -0.65 | 0.0187 | down |
| LOC_Os05g34325 | -0.62 | 0.0254 | down |
| LOC_Os05g35850 | -0.71 | 0.00347 | down |
| LOC_Os05g40700 | -0.68 | 0.0188 | down |
| LOC_Os05g40790 | -0.65 | 0.0219 | down |
| LOC_Os05g45160 | -0.58 | 6.69E-07 | down |
| LOC_Os05g47770 | -0.62 | 0.0369 | down |
| LOC_Os05g51360 | -0.68 | 0.0061 | down |
| LOC_Os06g04560 | -1.16 | 1.19E-05 | down |
| LOC_Os06g05284 | -0.85 | 0.00619 | down |
| LOC_Os06g06990 | -1.01 | 5.77E-10 | down |
| LOC_Os06g10880 | -0.68 | 0.00539 | down |
| LOC_Os06g13320 | -0.58 | 0.0267 | down |
| LOC_Os06g16660 | -0.9 | 0.000171 | down |
| LOC_Os06g19070 | -0.61 | 0.00127 | down |
| LOC_Os06g19500 | -0.62 | 0.00936 | down |
| LOC_Os06g20630 | -0.82 | 0.0214 | down |
| LOC_Os06g28480 | -0.58 | 0.0143 | down |
| LOC_Os06g35700 | -0.78 | 0.012 | down |
| LOC_Os06g38750 | -0.67 | 0.014 | down |
| LOC_Os06g39410 | -0.59 | 0.00532 | down |
| LOC_Os06g40570 | -0.71 | 0.015 | down |
| LOC_Os06g41570 | -0.62 | 0.0238 | down |
| LOC_Os06g43350 | -0.58 | 0.0143 | down |
| LOC_Os06g46680 | -0.77 | 0.0153 | down |
| LOC_Os06g46720 | -0.73 | 0.0244 | down |
| LOC_Os06g48060 | -0.62 | 0.00922 | down |
| LOC_Os06g50410 | -0.63 | 0.0116 | down |
| LOC_Os07g01904 | -1.18 | 3.87E-09 | down |
| LOC_Os07g03260 | -0.64 | 0.00254 | down |
| LOC_Os07g07510 | -0.63 | 0.0253 | down |
| LOC_Os07g08270 | -0.6 | 0.0253 | down |
| LOC_Os07g13910 | -0.67 | 0.0242 | down |
| LOC_Os07g14030 | -0.76 | 0.00445 | down |
| LOC_Os07g16980 | -0.79 | 0.00485 | down |
| LOC_Os07g17680 | -0.88 | 0.000102 | down |
| LOC_Os07g27050 | -0.67 | 0.00675 | down |
| LOC_Os07g29830 | -0.7 | 0.0278 | down |
| LOC_Os07g34140 | -0.85 | 0.0107 | down |
| LOC_Os07g35520 | -0.73 | 0.00519 | down |
| LOC_Os07g36040 | -0.58 | 0.022 | down |
| LOC_Os07g44840 | -0.71 | 0.0249 | down |
| LOC_Os07g46930 | -0.7 | 0.0396 | down |
| LOC_Os08g09700 | -0.66 | 0.0338 | down |
| LOC_Os08g10210 | -0.58 | 0.0211 | down |
| LOC_Os08g14180 | -0.84 | 0.00108 | down |
| LOC_Os08g16460 | -0.61 | 0.0119 | down |
| LOC_Os08g16610 | -0.9 | 0.00969 | down |
| LOC_Os08g22660 | -4.18 | 2.70E-15 | down |
| LOC_Os08g24830 | -0.74 | 0.0231 | down |
| LOC_Os08g25030 | -0.73 | 0.00381 | down |
| LOC_Os08g27824 | -1.01 | 1.15E-06 | down |
| LOC_Os08g30650 | -0.68 | 0.0129 | down |
| LOC_Os08g31740 | -0.58 | 0.00804 | down |
| LOC_Os08g32500 | -0.59 | 0.048 | down |
| LOC_Os08g34900 | -0.66 | 0.0028 | down |
| LOC_Os08g35050 | -0.62 | 0.0489 | down |
| LOC_Os08g35760 | -0.9 | 0.00219 | down |
| LOC_Os08g37970 | -0.63 | 0.0277 | down |
| LOC_Os08g40050 | -0.6 | 0.00943 | down |
| LOC_Os08g42630 | -0.77 | 3.75E-06 | down |
| LOC_Os08g45280 | -0.77 | 0.0152 | down |
| LOC_Os09g09370 | -0.59 | 0.00064 | down |
| LOC_Os09g10640 | -0.88 | 3.65E-09 | down |
| LOC_Os09g10650 | -1.6 | 1.07E-07 | down |
| LOC_Os09g15200 | -0.64 | 0.02 | down |
| LOC_Os09g16170 | -0.87 | 0.00136 | down |
| LOC_Os09g20550 | -0.61 | 0.0147 | down |
| LOC_Os09g21490 | -0.64 | 0.0386 | down |
| LOC_Os09g32880 | -0.76 | 3.39E-05 | down |
| LOC_Os09g33900 | -0.58 | 0.0212 | down |
| LOC_Os09g34950 | -0.71 | 3.88E-14 | down |
| LOC_Os09g36180 | -0.92 | 0.000672 | down |
| LOC_Os09g37400 | -0.69 | 0.0133 | down |
| LOC_Os09g38630 | -0.63 | 0.000741 | down |
| LOC_Os10g03850 | -0.6 | 0.0219 | down |
| LOC_Os10g07040 | -0.58 | 0.0362 | down |
| LOC_Os10g07160 | -1.13 | 6.09E-09 | down |
| LOC_Os10g09570 | -0.65 | 0.0251 | down |
| LOC_Os10g21590 | -0.88 | 0.00929 | down |
| LOC_Os10g22150 | -1.17 | 3.84E-27 | down |
| LOC_Os10g22410 | -0.79 | 0.021 | down |
| LOC_Os10g22830 | -1.04 | 0.000115 | down |
| LOC_Os10g22880 | -0.68 | 0.027 | down |
| LOC_Os10g31650 | -0.9 | 0.000533 | down |
| LOC_Os10g34110 | -0.67 | 0.000936 | down |
| LOC_Os10g41040 | -0.58 | 0.0333 | down |
| LOC_Os10g42920 | -0.6 | 0.021 | down |
| LOC_Os11g07360 | -0.72 | 0.000444 | down |
| LOC_Os11g10570 | -0.58 | 4.83E-08 | down |
| LOC_Os11g14410 | -0.62 | 0.0259 | down |
| LOC_Os11g16610 | -0.72 | 0.03 | down |
| LOC_Os11g17560 | -0.59 | 0.00184 | down |
| LOC_Os11g18980 | -0.58 | 0.0404 | down |
| LOC_Os11g20330 | -0.61 | 0.0192 | down |
| LOC_Os11g23220 | -0.96 | 0.00132 | down |
| LOC_Os11g24180 | -0.59 | 0.000485 | down |
| LOC_Os11g24890 | -0.74 | 0.0173 | down |
| LOC_Os11g24940 | -0.59 | 0.00765 | down |
| LOC_Os11g25990 | -0.92 | 0.000812 | down |
| LOC_Os11g30340 | -0.6 | 0.008 | down |
| LOC_Os11g35440 | -0.89 | 1.91E-07 | down |
| LOC_Os11g40490 | -0.62 | 0.00485 | down |
| LOC_Os11g43140 | -0.66 | 0.024 | down |
| LOC_Os11g43520 | -0.69 | 0.00259 | down |
| LOC_Os11g43990 | -0.59 | 0.0185 | down |
| LOC_Os11g45160 | -0.68 | 0.0185 | down |
| LOC_Os11g45330 | -0.61 | 0.0139 | down |
| LOC_Os11g47350 | -0.64 | 0.00405 | down |
| LOC_Os12g03840 | -2.12 | 8.15E-14 | down |
| LOC_Os12g04370 | -0.62 | 0.00589 | down |
| LOC_Os12g09520 | -0.67 | 0.00684 | down |
| LOC_Os12g12590 | -0.7 | 0.00869 | down |
| LOC_Os12g24050 | -2.53 | 3.18E-14 | down |
| LOC_Os12g27740 | -0.69 | 0.0309 | down |
| LOC_Os12g29360 | -1.14 | 0.000261 | down |
| LOC_Os12g29820 | -0.66 | 0.00352 | down |
| LOC_Os12g29874 | -0.63 | 0.0161 | down |
| LOC_Os12g31320 | -0.7 | 0.0136 | down |
| LOC_Os12g31540 | -0.61 | 0.0115 | down |
| LOC_Os12g31880 | -0.66 | 0.0309 | down |
| LOC_Os12g32499 | -0.71 | 0.00176 | down |
| LOC_Os12g33280 | -0.86 | 3.92E-07 | down |
| LOC_Os12g35000 | -0.8 | 0.000157 | down |
| LOC_Os12g36430 | -0.82 | 0.000419 | down |
| LOC_Os12g37510 | -0.6 | 0.0154 | down |
| LOC_Os12g38410 | -0.66 | 0.00955 | down |
| LOC_Os12g38420 | -1.27 | 1.73E-09 | down |

**Table S4.** H4K8ac up and downregulated genes in *ossrt2* mutant vs WT.

| **Gene ID** | **Log_2_Fold change** | ***P* value** | **Expression** |
| --- | --- | --- | --- |
| LOC_Os04g48440 | 9.33 | 1.08E-19 | up |
| LOC_Os04g21110 | 9 | 2.80E-18 | up |
| LOC_Os12g16550 | 8.84 | 1.03E-17 | up |
| LOC_Os04g14690 | 8.55 | 8.93E-24 | up |
| LOC_Os06g19444 | 8.46 | 3.45E-16 | up |
| LOC_Os05g08940 | 8.38 | 6.87E-16 | up |
| LOC_Os06g08240 | 8.25 | 2.18E-15 | up |
| LOC_Os09g02030 | 8.21 | 3.05E-15 | up |
| LOC_Os10g25040 | 7.95 | 3.09E-14 | up |
| LOC_Os12g07680 | 7.94 | 3.34E-14 | up |
| LOC_Os02g12470 | 7.89 | 5.18E-14 | up |
| LOC_Os02g01460 | 7.79 | 1.31E-13 | up |
| LOC_Os04g01910 | 7.74 | 1.80E-13 | up |
| LOC_Os09g10650 | 7.68 | 6.33E-19 | up |
| LOC_Os03g01520 | 7.64 | 3.99E-13 | up |
| LOC_Os11g05070 | 7.61 | 6.10E-13 | up |
| LOC_Os06g23090 | 7.58 | 6.77E-13 | up |
| LOC_Os05g37810 | 7.56 | 9.51E-13 | up |
| LOC_Os01g67134 | 7.55 | 8.50E-13 | up |
| LOC_Os10g24004 | 7.53 | 1.05E-12 | up |
| LOC_Os03g48890 | 7.48 | 1.50E-12 | up |
| LOC_Os05g39920 | 7.43 | 2.34E-12 | up |
| LOC_Os08g05460 | 7.39 | 3.04E-12 | up |
| LOC_Os06g37630 | 7.29 | 7.70E-12 | up |
| LOC_Os07g46190 | 7.25 | 9.29E-12 | up |
| LOC_Os12g02050 | 7.08 | 4.14E-11 | up |
| LOC_Os03g53090 | 6.95 | 1.25E-10 | up |
| LOC_Os11g02490 | 6.88 | 1.81E-10 | up |
| LOC_Os03g30750 | 6.66 | 1.07E-09 | up |
| LOC_Os09g33620 | 6.65 | 1.09E-09 | up |
| LOC_Os11g14860 | 6.64 | 1.37E-09 | up |
| LOC_Os10g40480 | 6.56 | 2.22E-09 | up |
| LOC_Os01g02670 | 6.54 | 3.07E-09 | up |
| LOC_Os06g14770 | 6.54 | 2.82E-09 | up |
| LOC_Os02g55280 | 6.51 | 3.45E-09 | up |
| LOC_Os07g40986 | 6.49 | 4.38E-09 | up |
| LOC_Os08g34060 | 6.49 | 3.85E-09 | up |
| LOC_Os09g38360 | 6.49 | 4.02E-09 | up |
| LOC_Os06g48750 | 6.47 | 5.01E-09 | up |
| LOC_Os03g27400 | 6.46 | 2.32E-16 | up |
| LOC_Os06g02450 | 6.37 | 1.07E-08 | up |
| LOC_Os12g05680 | 6.36 | 1.19E-08 | up |
| LOC_Os04g29270 | 6.33 | 1.37E-12 | up |
| LOC_Os05g39800 | 6.27 | 2.24E-18 | up |
| LOC_Os06g13680 | 6.23 | 3.97E-09 | up |
| LOC_Os03g15980 | 6.1 | 8.35E-08 | up |
| LOC_Os02g22090 | 6 | 1.64E-07 | up |
| LOC_Os03g26020 | 5.99 | 1.80E-07 | up |
| LOC_Os11g09060 | 5.9 | 7.60E-14 | up |
| LOC_Os10g02450 | 5.76 | 9.08E-07 | up |
| LOC_Os04g33590 | 5.72 | 1.35E-06 | up |
| LOC_Os08g04590 | 5.55 | 2.42E-13 | up |
| LOC_Os10g09330 | 5.49 | 4.98E-14 | up |
| LOC_Os03g12200 | 5.33 | 4.98E-08 | up |
| LOC_Os07g39630 | 5.31 | 6.19E-26 | up |
| LOC_Os10g07050 | 5.31 | 0.000131 | up |
| LOC_Os02g52660 | 5.25 | 3.53E-13 | up |
| LOC_Os02g12240 | 5.24 | 2.75E-25 | up |
| LOC_Os12g02320 | 5.2 | 4.26E-11 | up |
| LOC_Os10g05900 | 5.15 | 3.93E-18 | up |
| LOC_Os07g41400 | 4.93 | 5.33E-14 | up |
| LOC_Os09g10660 | 4.91 | 0.000112 | up |
| LOC_Os05g47920 | 4.78 | 4.97E-13 | up |
| LOC_Os05g06814 | 4.74 | 8.73E-05 | up |
| LOC_Os02g01220 | 4.73 | 5.40E-11 | up |
| LOC_Os07g05450 | 4.73 | 3.74E-11 | up |
| LOC_Os05g40890 | 4.72 | 1.29E-09 | up |
| LOC_Os12g40730 | 4.71 | 4.06E-09 | up |
| LOC_Os11g35300 | 4.62 | 2.30E-22 | up |
| LOC_Os10g10175 | 4.59 | 1.31E-28 | up |
| LOC_Os07g17280 | 4.53 | 8.04E-10 | up |
| LOC_Os12g12690 | 4.45 | 1.16E-12 | up |
| LOC_Os01g55400 | 4.39 | 6.18E-22 | up |
| LOC_Os09g15460 | 4.39 | 1.12E-05 | up |
| LOC_Os08g02870 | 4.37 | 9.22E-11 | up |
| LOC_Os09g37100 | 4.31 | 2.50E-08 | up |
| LOC_Os11g10190 | 4.3 | 1.69E-14 | up |
| LOC_Os03g61170 | 4.29 | 2.95E-13 | up |
| LOC_Os05g35970 | 4.27 | 0.000151 | up |
| LOC_Os12g39280 | 4.27 | 2.79E-31 | up |
| LOC_Os11g37140 | 4.26 | 0.00889 | up |
| LOC_Os10g33410 | 4.25 | 9.22E-05 | up |
| LOC_Os02g09080 | 4.18 | 7.75E-22 | up |
| LOC_Os05g42420 | 4.14 | 1.07E-11 | up |
| LOC_Os03g19090 | 4.13 | 3.32E-11 | up |
| LOC_Os06g22360 | 4.12 | 8.33E-11 | up |
| LOC_Os08g24800 | 4.09 | 3.13E-12 | up |
| LOC_Os02g02330 | 4.08 | 1.29E-13 | up |
| LOC_Os05g07080 | 4.08 | 0.000628 | up |
| LOC_Os12g02140 | 4.03 | 0.00141 | up |
| LOC_Os02g48560 | 4.01 | 1.41E-30 | up |
| LOC_Os02g57854 | 3.95 | 0.00651 | up |
| LOC_Os01g62540 | 3.92 | 0.00196 | up |
| LOC_Os05g44930 | 3.87 | 5.60E-06 | up |
| LOC_Os06g08350 | 3.87 | 3.04E-20 | up |
| LOC_Os02g49510 | 3.85 | 2.09E-14 | up |
| LOC_Os06g34540 | 3.83 | 9.42E-08 | up |
| LOC_Os08g23740 | 3.83 | 0.000476 | up |
| LOC_Os04g28850 | 3.81 | 0.00164 | up |
| LOC_Os04g43270 | 3.81 | 1.68E-07 | up |
| LOC_Os03g38250 | 3.76 | 0.000647 | up |
| LOC_Os12g29710 | 3.75 | 7.28E-07 | up |
| LOC_Os05g45150 | 3.73 | 1.79E-46 | up |
| LOC_Os06g05380 | 3.72 | 4.98E-14 | up |
| LOC_Os02g52730 | 3.71 | 3.17E-22 | up |
| LOC_Os01g42420 | 3.63 | 2.94E-39 | up |
| LOC_Os09g09370 | 3.63 | 1.99E-30 | up |
| LOC_Os01g55880 | 3.6 | 0.00293 | up |
| LOC_Os02g51510 | 3.6 | 4.51E-09 | up |
| LOC_Os08g16700 | 3.59 | 1.17E-26 | up |
| LOC_Os04g33440 | 3.56 | 7.97E-07 | up |
| LOC_Os05g45170 | 3.49 | 0.000197 | up |
| LOC_Os04g55140 | 3.42 | 3.44E-09 | up |
| LOC_Os05g43630 | 3.41 | 1.66E-37 | up |
| LOC_Os12g07180 | 3.4 | 1.26E-12 | up |
| LOC_Os03g02460 | 3.35 | 3.92E-46 | up |
| LOC_Os10g41340 | 3.35 | 4.40E-05 | up |
| LOC_Os05g12094 | 3.33 | 0.000527 | up |
| LOC_Os07g41080 | 3.33 | 0.00601 | up |
| LOC_Os12g36710 | 3.33 | 0.0375 | up |
| LOC_Os01g16950 | 3.29 | 5.70E-13 | up |
| LOC_Os05g24610 | 3.27 | 1.20E-55 | up |
| LOC_Os09g14610 | 3.26 | 9.74E-26 | up |
| LOC_Os01g15510 | 3.24 | 1.52E-21 | up |
| LOC_Os03g41650 | 3.22 | 1.32E-10 | up |
| LOC_Os06g14870 | 3.2 | 6.49E-21 | up |
| LOC_Os04g38540 | 3.19 | 1.31E-18 | up |
| LOC_Os09g02050 | 3.19 | 0.00147 | up |
| LOC_Os07g45970 | 3.17 | 0.0207 | up |
| LOC_Os09g06730 | 3.17 | 0.0115 | up |
| LOC_Os03g11720 | 3.15 | 9.39E-13 | up |
| LOC_Os04g19540 | 3.12 | 1.29E-09 | up |
| LOC_Os01g49740 | 3.09 | 1.70E-53 | up |
| LOC_Os03g36080 | 3.09 | 4.63E-23 | up |
| LOC_Os06g32330 | 3.09 | 0.000398 | up |
| LOC_Os05g23650 | 3.05 | 1.98E-20 | up |
| LOC_Os11g44750 | 3.05 | 4.13E-11 | up |
| LOC_Os07g10850 | 3.04 | 3.12E-19 | up |
| LOC_Os05g38700 | 3.03 | 3.69E-21 | up |
| LOC_Os06g49350 | 3.03 | 2.34E-28 | up |
| LOC_Os05g23050 | 3.02 | 3.26E-57 | up |
| LOC_Os06g20740 | 2.96 | 1.80E-05 | up |
| LOC_Os11g41590 | 2.93 | 0.000596 | up |
| LOC_Os03g18779 | 2.92 | 1.19E-53 | up |
| LOC_Os06g07410 | 2.92 | 1.25E-37 | up |
| LOC_Os03g26250 | 2.91 | 6.06E-24 | up |
| LOC_Os01g02090 | 2.9 | 1.17E-05 | up |
| LOC_Os04g42000 | 2.88 | 1.05E-27 | up |
| LOC_Os07g02030 | 2.87 | 3.30E-10 | up |
| LOC_Os10g21130 | 2.85 | 1.87E-11 | up |
| LOC_Os12g06060 | 2.84 | 1.92E-10 | up |
| LOC_Os06g19610 | 2.83 | 1.61E-05 | up |
| LOC_Os01g11620 | 2.72 | 5.04E-15 | up |
| LOC_Os08g39680 | 2.69 | 1.84E-07 | up |
| LOC_Os11g11694 | 2.66 | 0.00689 | up |
| LOC_Os05g34490 | 2.64 | 9.24E-29 | up |
| LOC_Os03g27200 | 2.62 | 0.00986 | up |
| LOC_Os04g31484 | 2.57 | 6.45E-06 | up |
| LOC_Os04g52790 | 2.56 | 3.50E-40 | up |
| LOC_Os02g30550 | 2.52 | 0.0132 | up |
| LOC_Os02g43010 | 2.51 | 5.69E-11 | up |
| LOC_Os04g32540 | 2.51 | 0.000151 | up |
| LOC_Os01g14830 | 2.5 | 2.09E-16 | up |
| LOC_Os05g23250 | 2.5 | 6.68E-34 | up |
| LOC_Os05g45779 | 2.48 | 7.14E-07 | up |
| LOC_Os03g41339 | 2.46 | 1.26E-12 | up |
| LOC_Os04g28870 | 2.44 | 1.02E-06 | up |
| LOC_Os06g14440 | 2.43 | 3.48E-16 | up |
| LOC_Os06g32590 | 2.43 | 2.14E-07 | up |
| LOC_Os03g58800 | 2.4 | 4.08E-12 | up |
| LOC_Os02g57860 | 2.39 | 1.51E-09 | up |
| LOC_Os11g02220 | 2.38 | 2.01E-10 | up |
| LOC_Os08g27678 | 2.35 | 1.54E-18 | up |
| LOC_Os07g08940 | 2.32 | 3.69E-12 | up |
| LOC_Os02g50404 | 2.3 | 3.05E-13 | up |
| LOC_Os09g28750 | 2.27 | 2.86E-10 | up |
| LOC_Os02g47990 | 2.26 | 1.36E-08 | up |
| LOC_Os04g32090 | 2.24 | 2.69E-05 | up |
| LOC_Os11g47190 | 2.23 | 0.00132 | up |
| LOC_Os03g25289 | 2.21 | 1.20E-09 | up |
| LOC_Os05g14730 | 2.2 | 5.50E-07 | up |
| LOC_Os02g53310 | 2.19 | 5.48E-12 | up |
| LOC_Os09g29360 | 2.19 | 5.28E-09 | up |
| LOC_Os12g41460 | 2.18 | 8.15E-08 | up |
| LOC_Os04g34500 | 2.16 | 0.000477 | up |
| LOC_Os08g43010 | 2.16 | 2.31E-05 | up |
| LOC_Os04g39864 | 2.14 | 8.63E-14 | up |
| LOC_Os03g45000 | 2.13 | 1.03E-24 | up |
| LOC_Os02g46680 | 2.12 | 0.0126 | up |
| LOC_Os03g62120 | 2.1 | 2.96E-07 | up |
| LOC_Os04g06840 | 2.1 | 5.74E-05 | up |
| LOC_Os05g28350 | 2.1 | 3.63E-06 | up |
| LOC_Os06g21369 | 2.1 | 3.94E-27 | up |
| LOC_Os07g41070 | 2.08 | 0.00173 | up |
| LOC_Os02g12510 | 2.07 | 1.03E-18 | up |
| LOC_Os08g31730 | 2.05 | 1.97E-20 | up |
| LOC_Os05g13650 | 2.04 | 0.0275 | up |
| LOC_Os06g09130 | 2.03 | 8.08E-11 | up |
| LOC_Os11g02230 | 2.02 | 3.13E-09 | up |
| LOC_Os04g16340 | 2.01 | 5.28E-07 | up |
| LOC_Os02g58310 | 1.99 | 0.000296 | up |
| LOC_Os03g10840 | 1.98 | 5.06E-11 | up |
| LOC_Os02g53230 | 1.97 | 1.42E-37 | up |
| LOC_Os12g35910 | 1.97 | 3.82E-08 | up |
| LOC_Os02g15430 | 1.96 | 4.44E-07 | up |
| LOC_Os02g21320 | 1.95 | 2.19E-12 | up |
| LOC_Os02g31845 | 1.94 | 7.11E-10 | up |
| LOC_Os04g27310 | 1.94 | 7.80E-19 | up |
| LOC_Os06g40240 | 1.93 | 1.60E-05 | up |
| LOC_Os01g56850 | 1.91 | 2.95E-06 | up |
| LOC_Os11g04030 | 1.91 | 9.53E-05 | up |
| LOC_Os03g53780 | 1.9 | 1.30E-05 | up |
| LOC_Os02g05030 | 1.87 | 0.000495 | up |
| LOC_Os02g04880 | 1.86 | 3.33E-06 | up |
| LOC_Os05g51820 | 1.85 | 6.71E-12 | up |
| LOC_Os08g03650 | 1.84 | 0.000179 | up |
| LOC_Os08g26880 | 1.84 | 2.68E-06 | up |
| LOC_Os01g01470 | 1.83 | 0.000487 | up |
| LOC_Os01g43110 | 1.83 | 0.00023 | up |
| LOC_Os06g43840 | 1.82 | 0.000287 | up |
| LOC_Os05g45700 | 1.8 | 1.61E-16 | up |
| LOC_Os03g08350 | 1.79 | 4.81E-13 | up |
| LOC_Os05g38530 | 1.78 | 0.00015 | up |
| LOC_Os03g31690 | 1.77 | 0.000492 | up |
| LOC_Os12g32499 | 1.76 | 0.00826 | up |
| LOC_Os06g15730 | 1.75 | 1.99E-12 | up |
| LOC_Os11g25980 | 1.75 | 4.79E-13 | up |
| LOC_Os11g34220 | 1.75 | 6.00E-06 | up |
| LOC_Os12g03480 | 1.75 | 1.04E-19 | up |
| LOC_Os10g29810 | 1.74 | 1.98E-06 | up |
| LOC_Os10g30150 | 1.73 | 5.29E-28 | up |
| LOC_Os10g04520 | 1.72 | 1.37E-12 | up |
| LOC_Os02g15160 | 1.7 | 4.58E-06 | up |
| LOC_Os02g49910 | 1.7 | 7.52E-11 | up |
| LOC_Os02g55250 | 1.7 | 0.000115 | up |
| LOC_Os08g36630 | 1.7 | 0.000173 | up |
| LOC_Os03g43100 | 1.69 | 4.48E-36 | up |
| LOC_Os07g44480 | 1.69 | 1.66E-09 | up |
| LOC_Os09g38104 | 1.69 | 1.05E-23 | up |
| LOC_Os10g21400 | 1.66 | 0.00092 | up |
| LOC_Os03g55460 | 1.64 | 1.38E-07 | up |
| LOC_Os09g35660 | 1.64 | 0.000527 | up |
| LOC_Os01g36860 | 1.62 | 0.00266 | up |
| LOC_Os08g31720 | 1.61 | 0.00764 | up |
| LOC_Os07g36610 | 1.6 | 0.00906 | up |
| LOC_Os02g14480 | 1.57 | 3.04E-06 | up |
| LOC_Os05g04770 | 1.57 | 0.00758 | up |
| LOC_Os06g03700 | 1.57 | 0.000971 | up |
| LOC_Os09g18470 | 1.57 | 7.09E-08 | up |
| LOC_Os02g50174 | 1.56 | 0.000696 | up |
| LOC_Os05g07170 | 1.56 | 1.60E-10 | up |
| LOC_Os01g48850 | 1.54 | 0.0126 | up |
| LOC_Os03g05340 | 1.54 | 1.49E-09 | up |
| LOC_Os08g01590 | 1.54 | 6.82E-07 | up |
| LOC_Os11g47010 | 1.54 | 0.00128 | up |
| LOC_Os12g12950 | 1.54 | 0.000124 | up |
| LOC_Os03g62224 | 1.52 | 4.84E-08 | up |
| LOC_Os06g40000 | 1.52 | 1.48E-10 | up |
| LOC_Os12g32390 | 1.52 | 2.42E-05 | up |
| LOC_Os07g01090 | 1.51 | 0.00749 | up |
| LOC_Os11g36880 | 1.51 | 0.000438 | up |
| LOC_Os01g53380 | 1.5 | 8.68E-06 | up |
| LOC_Os06g22610 | 1.5 | 1.44E-05 | up |
| LOC_Os07g18230 | 1.49 | 8.01E-05 | up |
| LOC_Os11g06200 | 1.49 | 1.80E-05 | up |
| LOC_Os02g57960 | 1.48 | 7.24E-07 | up |
| LOC_Os02g33540 | 1.47 | 0.00318 | up |
| LOC_Os07g09625 | 1.47 | 0.00535 | up |
| LOC_Os02g32814 | 1.46 | 1.30E-06 | up |
| LOC_Os08g09990 | 1.46 | 5.27E-18 | up |
| LOC_Os10g22410 | 1.46 | 9.93E-06 | up |
| LOC_Os02g11980 | 1.45 | 0.000291 | up |
| LOC_Os02g39020 | 1.45 | 0.00182 | up |
| LOC_Os12g35440 | 1.45 | 0.000314 | up |
| LOC_Os12g39380 | 1.45 | 1.54E-07 | up |
| LOC_Os01g46610 | 1.44 | 0.00251 | up |
| LOC_Os05g12130 | 1.43 | 0.000874 | up |
| LOC_Os10g35090 | 1.43 | 0.000344 | up |
| LOC_Os02g06630 | 1.42 | 5.12E-05 | up |
| LOC_Os06g11490 | 1.42 | 0.0033 | up |
| LOC_Os11g33100 | 1.42 | 3.96E-06 | up |
| LOC_Os02g06010 | 1.41 | 3.51E-07 | up |
| LOC_Os02g37770 | 1.41 | 0.0011 | up |
| LOC_Os03g18140 | 1.41 | 1.15E-10 | up |
| LOC_Os09g25370 | 1.41 | 0.0116 | up |
| LOC_Os12g02170 | 1.41 | 8.25E-06 | up |
| LOC_Os05g24160 | 1.4 | 7.32E-08 | up |
| LOC_Os03g35326 | 1.38 | 1.94E-08 | up |
| LOC_Os10g35170 | 1.38 | 1.56E-13 | up |
| LOC_Os12g21784 | 1.38 | 0.00293 | up |
| LOC_Os01g29390 | 1.37 | 0.00519 | up |
| LOC_Os06g35550 | 1.37 | 0.00686 | up |
| LOC_Os01g40640 | 1.35 | 0.0468 | up |
| LOC_Os03g07410 | 1.35 | 0.00492 | up |
| LOC_Os08g28560 | 1.35 | 0.00514 | up |
| LOC_Os10g39140 | 1.35 | 0.000882 | up |
| LOC_Os02g02424 | 1.34 | 0.00426 | up |
| LOC_Os02g27550 | 1.34 | 0.000461 | up |
| LOC_Os05g39860 | 1.34 | 0.000297 | up |
| LOC_Os06g48980 | 1.34 | 2.97E-06 | up |
| LOC_Os09g17530 | 1.34 | 0.000127 | up |
| LOC_Os09g25300 | 1.34 | 5.98E-07 | up |
| LOC_Os12g26470 | 1.34 | 1.45E-06 | up |
| LOC_Os02g46260 | 1.33 | 0.0145 | up |
| LOC_Os03g42270 | 1.33 | 8.52E-06 | up |
| LOC_Os03g51270 | 1.33 | 2.28E-06 | up |
| LOC_Os06g12882 | 1.33 | 0.0153 | up |
| LOC_Os09g29920 | 1.32 | 0.00598 | up |
| LOC_Os07g46080 | 1.31 | 9.15E-06 | up |
| LOC_Os09g18250 | 1.31 | 0.00838 | up |
| LOC_Os06g06080 | 1.3 | 0.000855 | up |
| LOC_Os12g09570 | 1.3 | 0.00161 | up |
| LOC_Os12g39700 | 1.3 | 9.48E-05 | up |
| LOC_Os01g10520 | 1.29 | 0.00759 | up |
| LOC_Os02g54830 | 1.29 | 0.0311 | up |
| LOC_Os03g04750 | 1.29 | 0.0364 | up |
| LOC_Os06g11170 | 1.29 | 0.00734 | up |
| LOC_Os01g70850 | 1.28 | 6.48E-05 | up |
| LOC_Os06g49410 | 1.28 | 3.67E-06 | up |
| LOC_Os01g27230 | 1.27 | 2.22E-09 | up |
| LOC_Os01g43700 | 1.27 | 8.98E-08 | up |
| LOC_Os01g70210 | 1.27 | 0.0371 | up |
| LOC_Os02g05980 | 1.27 | 0.0117 | up |
| LOC_Os01g43710 | 1.26 | 0.0169 | up |
| LOC_Os07g47940 | 1.26 | 0.0189 | up |
| LOC_Os09g28489 | 1.25 | 0.0103 | up |
| LOC_Os11g34640 | 1.25 | 0.00388 | up |
| LOC_Os02g39640 | 1.24 | 1.43E-07 | up |
| LOC_Os01g42900 | 1.23 | 0.00916 | up |
| LOC_Os08g40600 | 1.23 | 0.0139 | up |
| LOC_Os09g11320 | 1.23 | 1.30E-06 | up |
| LOC_Os09g28354 | 1.23 | 0.00707 | up |
| LOC_Os10g41980 | 1.23 | 2.71E-05 | up |
| LOC_Os11g08569 | 1.23 | 6.19E-17 | up |
| LOC_Os01g59730 | 1.22 | 0.0123 | up |
| LOC_Os09g16570 | 1.22 | 0.00614 | up |
| LOC_Os09g22160 | 1.22 | 0.00486 | up |
| LOC_Os01g71106 | 1.21 | 0.00144 | up |
| LOC_Os03g06220 | 1.21 | 0.0139 | up |
| LOC_Os06g42890 | 1.21 | 0.000217 | up |
| LOC_Os09g25760 | 1.21 | 1.25E-07 | up |
| LOC_Os11g29030 | 1.21 | 1.49E-14 | up |
| LOC_Os02g52120 | 1.2 | 0.00922 | up |
| LOC_Os06g13720 | 1.2 | 0.0242 | up |
| LOC_Os01g33370 | 1.19 | 0.00782 | up |
| LOC_Os01g48300 | 1.19 | 0.000304 | up |
| LOC_Os03g24180 | 1.19 | 0.00837 | up |
| LOC_Os08g42640 | 1.19 | 0.000631 | up |
| LOC_Os03g63480 | 1.17 | 0.0282 | up |
| LOC_Os02g47270 | 1.16 | 0.00325 | up |
| LOC_Os04g19380 | 1.16 | 0.0172 | up |
| LOC_Os07g08680 | 1.16 | 6.53E-06 | up |
| LOC_Os11g32240 | 1.16 | 0.00487 | up |
| LOC_Os01g58290 | 1.15 | 0.0045 | up |
| LOC_Os04g32310 | 1.15 | 4.22E-05 | up |
| LOC_Os09g23810 | 1.15 | 0.0246 | up |
| LOC_Os12g12000 | 1.15 | 0.00868 | up |
| LOC_Os05g35870 | 1.14 | 0.0111 | up |
| LOC_Os06g35200 | 1.14 | 1.05E-07 | up |
| LOC_Os07g02620 | 1.14 | 1.18E-05 | up |
| LOC_Os01g47400 | 1.13 | 0.0013 | up |
| LOC_Os01g71940 | 1.13 | 6.56E-06 | up |
| LOC_Os02g55020 | 1.13 | 0.0241 | up |
| LOC_Os03g61110 | 1.13 | 0.0162 | up |
| LOC_Os09g09540 | 1.13 | 0.000611 | up |
| LOC_Os01g32660 | 1.12 | 0.0037 | up |
| LOC_Os06g33350 | 1.12 | 0.000855 | up |
| LOC_Os06g39110 | 1.12 | 0.000263 | up |
| LOC_Os01g42960 | 1.11 | 0.00431 | up |
| LOC_Os02g56500 | 1.11 | 0.019 | up |
| LOC_Os03g08970 | 1.11 | 0.00561 | up |
| LOC_Os05g07420 | 1.11 | 0.00103 | up |
| LOC_Os07g32406 | 1.11 | 0.00297 | up |
| LOC_Os07g48450 | 1.11 | 0.0251 | up |
| LOC_Os01g43760 | 1.1 | 0.00204 | up |
| LOC_Os03g58250 | 1.1 | 0.0465 | up |
| LOC_Os07g02610 | 1.09 | 0.0053 | up |
| LOC_Os10g39190 | 1.09 | 1.06E-10 | up |
| LOC_Os11g37970 | 1.09 | 0.00842 | up |
| LOC_Os02g15660 | 1.07 | 0.0149 | up |
| LOC_Os04g34590 | 1.07 | 0.0246 | up |
| LOC_Os04g48330 | 1.07 | 0.0029 | up |
| LOC_Os07g11070 | 1.07 | 0.0124 | up |
| LOC_Os09g35590 | 1.07 | 0.027 | up |
| LOC_Os01g70410 | 1.06 | 0.0287 | up |
| LOC_Os02g21190 | 1.06 | 0.00786 | up |
| LOC_Os03g01650 | 1.06 | 4.87E-05 | up |
| LOC_Os03g64400 | 1.06 | 0.0397 | up |
| LOC_Os04g34800 | 1.06 | 0.00236 | up |
| LOC_Os04g42430 | 1.06 | 0.0182 | up |
| LOC_Os05g45670 | 1.06 | 8.87E-05 | up |
| LOC_Os07g31450 | 1.06 | 0.000148 | up |
| LOC_Os07g35960 | 1.06 | 0.0052 | up |
| LOC_Os01g61720 | 1.05 | 0.000846 | up |
| LOC_Os06g43290 | 1.05 | 0.00545 | up |
| LOC_Os08g07700 | 1.05 | 0.0101 | up |
| LOC_Os12g36830 | 1.05 | 0.0164 | up |
| LOC_Os01g07030 | 1.04 | 0.000849 | up |
| LOC_Os03g20300 | 1.04 | 0.00846 | up |
| LOC_Os03g28230 | 1.04 | 0.0146 | up |
| LOC_Os04g37520 | 1.04 | 0.0245 | up |
| LOC_Os04g53640 | 1.04 | 0.0211 | up |
| LOC_Os12g17160 | 1.04 | 0.0101 | up |
| LOC_Os01g24480 | 1.03 | 0.000173 | up |
| LOC_Os03g18200 | 1.03 | 0.00141 | up |
| LOC_Os04g58810 | 1.03 | 0.0118 | up |
| LOC_Os07g04570 | 1.03 | 0.00384 | up |
| LOC_Os04g57780 | 1.02 | 0.000861 | up |
| LOC_Os06g42630 | 1.02 | 1.01E-05 | up |
| LOC_Os07g13770 | 1.02 | 1.79E-10 | up |
| LOC_Os07g35140 | 1.02 | 0.0104 | up |
| LOC_Os09g29650 | 1.02 | 0.0411 | up |
| LOC_Os10g35770 | 1.02 | 0.0157 | up |
| LOC_Os12g03040 | 1.02 | 0.0249 | up |
| LOC_Os03g01610 | 1.01 | 7.92E-05 | up |
| LOC_Os03g06940 | 1.01 | 0.0364 | up |
| LOC_Os03g54150 | 1.01 | 0.0216 | up |
| LOC_Os04g46210 | 1.01 | 0.00126 | up |
| LOC_Os04g54390 | 1.01 | 0.0189 | up |
| LOC_Os07g02120 | 1.01 | 0.0118 | up |
| LOC_Os11g28950 | 1.01 | 0.0331 | up |
| LOC_Os12g38740 | 1.01 | 0.00546 | up |
| LOC_Os01g72730 | 1 | 0.000495 | up |
| LOC_Os04g36590 | 1 | 0.0137 | up |
| LOC_Os05g40300 | 1 | 0.0263 | up |
| LOC_Os09g39034 | 1 | 0.000184 | up |
| LOC_Os01g41960 | 0.99 | 4.24E-21 | up |
| LOC_Os02g06160 | 0.99 | 0.00405 | up |
| LOC_Os02g10760 | 0.99 | 8.12E-05 | up |
| LOC_Os06g41360 | 0.99 | 0.0312 | up |
| LOC_Os09g20880 | 0.99 | 0.0144 | up |
| LOC_Os10g21630 | 0.99 | 0.000873 | up |
| LOC_Os11g10840 | 0.99 | 0.00455 | up |
| LOC_Os07g05740 | 0.98 | 0.024 | up |
| LOC_Os07g10530 | 0.98 | 0.0114 | up |
| LOC_Os07g42490 | 0.98 | 0.0276 | up |
| LOC_Os07g42560 | 0.98 | 0.00367 | up |
| LOC_Os11g08235 | 0.98 | 0.0124 | up |
| LOC_Os01g55930 | 0.97 | 0.00713 | up |
| LOC_Os02g15310 | 0.97 | 0.0097 | up |
| LOC_Os03g03610 | 0.97 | 0.000207 | up |
| LOC_Os03g06890 | 0.97 | 0.0104 | up |
| LOC_Os04g38270 | 0.97 | 5.89E-13 | up |
| LOC_Os05g49600 | 0.97 | 0.00747 | up |
| LOC_Os06g48880 | 0.97 | 0.0036 | up |
| LOC_Os09g25980 | 0.97 | 0.00397 | up |
| LOC_Os11g10710 | 0.97 | 0.0135 | up |
| LOC_Os02g56470 | 0.96 | 0.000632 | up |
| LOC_Os07g07260 | 0.96 | 0.038 | up |
| LOC_Os07g10550 | 0.96 | 0.0127 | up |
| LOC_Os07g37220 | 0.96 | 0.00836 | up |
| LOC_Os01g65290 | 0.95 | 0.00984 | up |
| LOC_Os02g06210 | 0.95 | 0.0198 | up |
| LOC_Os03g01540 | 0.95 | 0.0236 | up |
| LOC_Os03g05390 | 0.95 | 0.012 | up |
| LOC_Os04g39570 | 0.95 | 0.0347 | up |
| LOC_Os05g29974 | 0.95 | 0.00606 | up |
| LOC_Os01g70180 | 0.94 | 0.00516 | up |
| LOC_Os05g46030 | 0.94 | 0.0195 | up |
| LOC_Os07g03860 | 0.94 | 0.0123 | up |
| LOC_Os08g43420 | 0.94 | 0.0235 | up |
| LOC_Os10g34602 | 0.94 | 3.20E-17 | up |
| LOC_Os01g49790 | 0.93 | 0.00773 | up |
| LOC_Os01g62860 | 0.93 | 0.00338 | up |
| LOC_Os02g06205 | 0.93 | 0.0354 | up |
| LOC_Os02g45860 | 0.93 | 0.0104 | up |
| LOC_Os03g30890 | 0.93 | 0.00483 | up |
| LOC_Os03g60820 | 0.93 | 0.0326 | up |
| LOC_Os04g21890 | 0.93 | 0.0346 | up |
| LOC_Os04g45860 | 0.93 | 0.00988 | up |
| LOC_Os05g50390 | 0.93 | 1.32E-09 | up |
| LOC_Os06g12090 | 0.93 | 0.0444 | up |
| LOC_Os07g46070 | 0.93 | 0.0315 | up |
| LOC_Os08g28890 | 0.93 | 0.0309 | up |
| LOC_Os08g39250 | 0.93 | 0.0255 | up |
| LOC_Os08g45010 | 0.93 | 0.018 | up |
| LOC_Os09g13540 | 0.93 | 4.57E-10 | up |
| LOC_Os12g07500 | 0.93 | 0.017 | up |
| LOC_Os01g40040 | 0.92 | 0.042 | up |
| LOC_Os02g04190 | 0.92 | 0.0198 | up |
| LOC_Os03g09310 | 0.92 | 0.0375 | up |
| LOC_Os03g53440 | 0.92 | 0.00434 | up |
| LOC_Os03g60580 | 0.92 | 0.0153 | up |
| LOC_Os03g63870 | 0.92 | 0.0386 | up |
| LOC_Os05g50700 | 0.92 | 0.0026 | up |
| LOC_Os10g33790 | 0.92 | 0.0269 | up |
| LOC_Os01g54420 | 0.91 | 0.0244 | up |
| LOC_Os02g21040 | 0.91 | 0.00317 | up |
| LOC_Os03g22050 | 0.91 | 0.0184 | up |
| LOC_Os03g28430 | 0.91 | 0.0224 | up |
| LOC_Os09g07810 | 0.91 | 0.000419 | up |
| LOC_Os10g07460 | 0.91 | 2.19E-07 | up |
| LOC_Os11g27530 | 0.91 | 0.0401 | up |
| LOC_Os01g57660 | 0.9 | 0.0485 | up |
| LOC_Os04g39350 | 0.9 | 0.0142 | up |
| LOC_Os04g41160 | 0.9 | 0.0433 | up |
| LOC_Os04g55640 | 0.9 | 0.0121 | up |
| LOC_Os05g01350 | 0.9 | 0.00228 | up |
| LOC_Os06g04060 | 0.9 | 0.0103 | up |
| LOC_Os09g37040 | 0.9 | 0.0429 | up |
| LOC_Os01g65920 | 0.89 | 0.0154 | up |
| LOC_Os02g12260 | 0.89 | 0.00391 | up |
| LOC_Os03g19080 | 0.89 | 0.0211 | up |
| LOC_Os07g49460 | 0.89 | 0.048 | up |
| LOC_Os11g02540 | 0.89 | 0.0258 | up |
| LOC_Os12g42020 | 0.89 | 0.00269 | up |
| LOC_Os01g20900 | 0.88 | 0.0304 | up |
| LOC_Os01g39260 | 0.88 | 0.0252 | up |
| LOC_Os01g43160 | 0.88 | 0.00481 | up |
| LOC_Os01g57440 | 0.88 | 0.00525 | up |
| LOC_Os02g42810 | 0.88 | 0.00468 | up |
| LOC_Os02g45080 | 0.88 | 0.000719 | up |
| LOC_Os03g08960 | 0.88 | 0.0105 | up |
| LOC_Os03g22020 | 0.88 | 0.045 | up |
| LOC_Os04g52630 | 0.88 | 0.0301 | up |
| LOC_Os06g28260 | 0.88 | 0.034 | up |
| LOC_Os06g34040 | 0.88 | 0.0221 | up |
| LOC_Os09g34990 | 0.88 | 0.0332 | up |
| LOC_Os09g37060 | 0.88 | 0.0452 | up |
| LOC_Os10g39220 | 0.88 | 0.0445 | up |
| LOC_Os01g15810 | 0.87 | 0.0493 | up |
| LOC_Os08g40820 | 0.87 | 0.0293 | up |
| LOC_Os11g10250 | 0.87 | 1.44E-07 | up |
| LOC_Os11g11990 | 0.87 | 1.24E-06 | up |
| LOC_Os01g53240 | 0.86 | 0.0144 | up |
| LOC_Os01g71720 | 0.86 | 0.00809 | up |
| LOC_Os03g06520 | 0.86 | 0.0263 | up |
| LOC_Os03g53660 | 0.86 | 0.0438 | up |
| LOC_Os05g45120 | 0.86 | 2.17E-07 | up |
| LOC_Os07g42310 | 0.86 | 0.0428 | up |
| LOC_Os09g26390 | 0.86 | 0.000879 | up |
| LOC_Os09g37740 | 0.86 | 0.0483 | up |
| LOC_Os10g22780 | 0.86 | 0.0029 | up |
| LOC_Os01g08830 | 0.85 | 0.0288 | up |
| LOC_Os02g55590 | 0.85 | 0.011 | up |
| LOC_Os03g22690 | 0.85 | 0.0469 | up |
| LOC_Os03g63740 | 0.85 | 0.0319 | up |
| LOC_Os05g31920 | 0.85 | 0.0369 | up |
| LOC_Os05g35100 | 0.85 | 0.0424 | up |
| LOC_Os05g39560 | 0.85 | 0.00487 | up |
| LOC_Os07g44499 | 0.85 | 0.0364 | up |
| LOC_Os08g33740 | 0.85 | 0.0324 | up |
| LOC_Os10g33855 | 0.85 | 0.0349 | up |
| LOC_Os01g61140 | 0.84 | 0.0137 | up |
| LOC_Os03g25464 | 0.84 | 0.00905 | up |
| LOC_Os03g58380 | 0.84 | 0.0184 | up |
| LOC_Os04g25100 | 0.84 | 4.26E-14 | up |
| LOC_Os08g11150 | 0.84 | 0.0163 | up |
| LOC_Os10g20360 | 0.84 | 0.0328 | up |
| LOC_Os10g33540 | 0.84 | 0.000248 | up |
| LOC_Os11g16770 | 0.84 | 0.0201 | up |
| LOC_Os01g05040 | 0.83 | 0.0359 | up |
| LOC_Os01g57560 | 0.83 | 0.0151 | up |
| LOC_Os01g70150 | 0.83 | 0.0181 | up |
| LOC_Os01g71960 | 0.83 | 0.0058 | up |
| LOC_Os01g74300 | 0.83 | 0.00559 | up |
| LOC_Os02g55330 | 0.83 | 0.0442 | up |
| LOC_Os03g09270 | 0.83 | 0.0498 | up |
| LOC_Os03g28960 | 0.83 | 0.0373 | up |
| LOC_Os04g01990 | 0.83 | 0.0302 | up |
| LOC_Os04g12080 | 0.83 | 0.011 | up |
| LOC_Os06g09970 | 0.83 | 0.0176 | up |
| LOC_Os06g21240 | 0.83 | 0.0308 | up |
| LOC_Os07g26490 | 0.83 | 0.0316 | up |
| LOC_Os07g30980 | 0.83 | 0.0343 | up |
| LOC_Os09g37760 | 0.83 | 0.0207 | up |
| LOC_Os11g43240 | 0.83 | 0.00124 | up |
| LOC_Os01g60040 | 0.82 | 0.023 | up |
| LOC_Os01g70470 | 0.82 | 0.0301 | up |
| LOC_Os01g70810 | 0.82 | 0.00439 | up |
| LOC_Os02g53670 | 0.82 | 0.00168 | up |
| LOC_Os03g55784 | 0.82 | 0.0136 | up |
| LOC_Os03g58060 | 0.82 | 0.0165 | up |
| LOC_Os04g34370 | 0.82 | 0.000265 | up |
| LOC_Os04g45980 | 0.82 | 0.00893 | up |
| LOC_Os05g28650 | 0.82 | 0.0393 | up |
| LOC_Os06g43520 | 0.82 | 0.0289 | up |
| LOC_Os06g50910 | 0.82 | 0.04 | up |
| LOC_Os07g31220 | 0.82 | 0.0338 | up |
| LOC_Os08g01940 | 0.82 | 0.0124 | up |
| LOC_Os09g35670 | 0.82 | 0.0487 | up |
| LOC_Os11g06390 | 0.82 | 0.00963 | up |
| LOC_Os11g32780 | 0.82 | 0.0272 | up |
| LOC_Os01g53810 | 0.81 | 0.0158 | up |
| LOC_Os03g21240 | 0.81 | 0.00149 | up |
| LOC_Os03g27930 | 0.81 | 0.0116 | up |
| LOC_Os03g62060 | 0.81 | 0.0207 | up |
| LOC_Os04g10050 | 0.81 | 0.00261 | up |
| LOC_Os04g38940 | 0.81 | 0.0272 | up |
| LOC_Os04g44670 | 0.81 | 0.0228 | up |
| LOC_Os04g48070 | 0.81 | 0.0241 | up |
| LOC_Os06g19370 | 0.81 | 0.0368 | up |
| LOC_Os06g38220 | 0.81 | 0.0187 | up |
| LOC_Os09g37390 | 0.81 | 0.0128 | up |
| LOC_Os01g06140 | 0.8 | 0.0438 | up |
| LOC_Os01g21250 | 0.8 | 0.00894 | up |
| LOC_Os01g60700 | 0.8 | 0.0263 | up |
| LOC_Os01g73130 | 0.8 | 0.0279 | up |
| LOC_Os02g05930 | 0.8 | 0.00222 | up |
| LOC_Os02g10650 | 0.8 | 0.0167 | up |
| LOC_Os03g14210 | 0.8 | 0.0323 | up |
| LOC_Os03g56720 | 0.8 | 0.00551 | up |
| LOC_Os03g61840 | 0.8 | 0.0448 | up |
| LOC_Os05g39690 | 0.8 | 0.0384 | up |
| LOC_Os06g13860 | 0.8 | 0.0197 | up |
| LOC_Os10g37970 | 0.8 | 0.0348 | up |
| LOC_Os01g10130 | 0.79 | 0.044 | up |
| LOC_Os02g09290 | 0.79 | 0.0287 | up |
| LOC_Os02g20210 | 0.79 | 0.0214 | up |
| LOC_Os02g44670 | 0.79 | 0.0293 | up |
| LOC_Os03g04290 | 0.79 | 0.0162 | up |
| LOC_Os03g22870 | 0.79 | 0.018 | up |
| LOC_Os04g17660 | 0.79 | 4.51E-11 | up |
| LOC_Os04g27790 | 0.79 | 0.0234 | up |
| LOC_Os04g52780 | 0.79 | 0.0327 | up |
| LOC_Os05g12720 | 0.79 | 0.0326 | up |
| LOC_Os05g51690 | 0.79 | 0.0232 | up |
| LOC_Os06g41640 | 0.79 | 0.000141 | up |
| LOC_Os07g01740 | 0.79 | 0.0388 | up |
| LOC_Os07g43925 | 0.79 | 0.0331 | up |
| LOC_Os08g01680 | 0.79 | 0.0121 | up |
| LOC_Os08g07320 | 0.79 | 1.94E-08 | up |
| LOC_Os08g44590 | 0.79 | 0.00781 | up |
| LOC_Os10g04110 | 0.79 | 3.47E-06 | up |
| LOC_Os11g47820 | 0.79 | 0.0014 | up |
| LOC_Os02g08100 | 0.78 | 0.00625 | up |
| LOC_Os02g35560 | 0.78 | 0.0285 | up |
| LOC_Os02g43660 | 0.78 | 0.0059 | up |
| LOC_Os02g52720 | 0.78 | 8.70E-09 | up |
| LOC_Os03g10620 | 0.78 | 0.0185 | up |
| LOC_Os06g48665 | 0.78 | 0.0442 | up |
| LOC_Os07g01904 | 0.78 | 4.80E-06 | up |
| LOC_Os07g02630 | 0.78 | 0.0476 | up |
| LOC_Os08g16910 | 0.78 | 0.0267 | up |
| LOC_Os08g37970 | 0.78 | 0.0247 | up |
| LOC_Os10g28760 | 0.78 | 0.0417 | up |
| LOC_Os12g25370 | 0.78 | 0.00676 | up |
| LOC_Os01g15130 | 0.77 | 0.0152 | up |
| LOC_Os01g57480 | 0.77 | 0.00474 | up |
| LOC_Os02g37290 | 0.77 | 0.042 | up |
| LOC_Os03g14700 | 0.77 | 0.0457 | up |
| LOC_Os03g29850 | 0.77 | 0.00921 | up |
| LOC_Os03g60470 | 0.77 | 0.0154 | up |
| LOC_Os04g31390 | 0.77 | 0.0142 | up |
| LOC_Os04g45740 | 0.77 | 0.00413 | up |
| LOC_Os07g36740 | 0.77 | 0.0449 | up |
| LOC_Os08g14910 | 0.77 | 0.0148 | up |
| LOC_Os10g31040 | 0.77 | 0.0447 | up |
| LOC_Os12g04980 | 0.77 | 0.0201 | up |
| LOC_Os12g08720 | 0.77 | 0.0121 | up |
| LOC_Os01g38140 | 0.76 | 0.0432 | up |
| LOC_Os01g43170 | 0.76 | 0.000511 | up |
| LOC_Os01g59340 | 0.76 | 0.00116 | up |
| LOC_Os02g36860 | 0.76 | 0.000313 | up |
| LOC_Os02g44130 | 0.76 | 0.0362 | up |
| LOC_Os02g47660 | 0.76 | 0.0013 | up |
| LOC_Os02g56110 | 0.76 | 0.0272 | up |
| LOC_Os03g06930 | 0.76 | 0.0465 | up |
| LOC_Os03g11734 | 0.76 | 0.033 | up |
| LOC_Os04g14400 | 0.76 | 6.39E-06 | up |
| LOC_Os04g55200 | 0.76 | 0.049 | up |
| LOC_Os06g47760 | 0.76 | 0.0239 | up |
| LOC_Os07g42750 | 0.76 | 0.0345 | up |
| LOC_Os08g16330 | 0.76 | 0.0317 | up |
| LOC_Os10g35140 | 0.76 | 0.0171 | up |
| LOC_Os10g35370 | 0.76 | 0.0075 | up |
| LOC_Os11g08970 | 0.76 | 0.0193 | up |
| LOC_Os11g36810 | 0.76 | 1.51E-09 | up |
| LOC_Os12g14764 | 0.76 | 0.00727 | up |
| LOC_Os12g24050 | 0.76 | 0.0114 | up |
| LOC_Os12g42044 | 0.76 | 0.00307 | up |
| LOC_Os01g53010 | 0.75 | 0.0402 | up |
| LOC_Os02g36890 | 0.75 | 0.0383 | up |
| LOC_Os02g42100 | 0.75 | 0.00999 | up |
| LOC_Os03g28330 | 0.75 | 0.0244 | up |
| LOC_Os04g35890 | 0.75 | 0.00604 | up |
| LOC_Os05g23950 | 0.75 | 1.49E-05 | up |
| LOC_Os12g02180 | 0.75 | 0.000257 | up |
| LOC_Os01g59819 | 0.74 | 0.0256 | up |
| LOC_Os02g44570 | 0.74 | 0.0498 | up |
| LOC_Os03g46470 | 0.74 | 0.0452 | up |
| LOC_Os05g10320 | 0.74 | 0.000443 | up |
| LOC_Os05g19680 | 0.74 | 0.025 | up |
| LOC_Os05g44390 | 0.74 | 0.000675 | up |
| LOC_Os07g05820 | 0.74 | 0.00606 | up |
| LOC_Os09g37590 | 0.74 | 0.0226 | up |
| LOC_Os10g03690 | 0.74 | 0.0314 | up |
| LOC_Os01g09860 | 0.73 | 0.00144 | up |
| LOC_Os01g57735 | 0.73 | 0.00839 | up |
| LOC_Os04g57860 | 0.73 | 0.0176 | up |
| LOC_Os07g40480 | 0.73 | 0.0359 | up |
| LOC_Os07g42770 | 0.73 | 0.00953 | up |
| LOC_Os01g11190 | 0.72 | 0.0194 | up |
| LOC_Os02g09580 | 0.72 | 0.0362 | up |
| LOC_Os02g38130 | 0.72 | 0.047 | up |
| LOC_Os02g43730 | 0.72 | 0.00721 | up |
| LOC_Os03g12550 | 0.72 | 0.00368 | up |
| LOC_Os04g12660 | 0.72 | 0.0123 | up |
| LOC_Os04g49110 | 0.72 | 0.0328 | up |
| LOC_Os05g43910 | 0.72 | 0.0461 | up |
| LOC_Os06g51260 | 0.72 | 0.0229 | up |
| LOC_Os07g42700 | 0.72 | 0.0242 | up |
| LOC_Os10g40360 | 0.72 | 0.000594 | up |
| LOC_Os12g01370 | 0.72 | 0.0185 | up |
| LOC_Os12g01449 | 0.72 | 0.000104 | up |
| LOC_Os01g05820 | 0.71 | 0.0145 | up |
| LOC_Os01g11650 | 0.71 | 0.0376 | up |
| LOC_Os01g62050 | 0.71 | 0.00186 | up |
| LOC_Os01g63520 | 0.71 | 0.0497 | up |
| LOC_Os02g57340 | 0.71 | 0.0217 | up |
| LOC_Os03g04610 | 0.71 | 0.0252 | up |
| LOC_Os03g17730 | 0.71 | 0.0232 | up |
| LOC_Os03g19580 | 0.71 | 0.0368 | up |
| LOC_Os03g52150 | 0.71 | 0.0292 | up |
| LOC_Os03g64320 | 0.71 | 0.0242 | up |
| LOC_Os05g12680 | 0.71 | 0.00222 | up |
| LOC_Os06g09980 | 0.71 | 0.000177 | up |
| LOC_Os06g44210 | 0.71 | 0.019 | up |
| LOC_Os07g30990 | 0.71 | 0.0127 | up |
| LOC_Os07g35580 | 0.71 | 0.0248 | up |
| LOC_Os07g37850 | 0.71 | 0.048 | up |
| LOC_Os08g10612 | 0.71 | 0.0485 | up |
| LOC_Os09g25280 | 0.71 | 0.00549 | up |
| LOC_Os09g36530 | 0.71 | 0.0206 | up |
| LOC_Os10g33900 | 0.71 | 0.0428 | up |
| LOC_Os12g35000 | 0.71 | 0.0345 | up |
| LOC_Os01g02420 | 0.7 | 0.00973 | up |
| LOC_Os01g04720 | 0.7 | 0.0391 | up |
| LOC_Os02g13555 | 0.7 | 0.0174 | up |
| LOC_Os02g35470 | 0.7 | 0.0181 | up |
| LOC_Os02g51100 | 0.7 | 0.05 | up |
| LOC_Os02g56190 | 0.7 | 0.05 | up |
| LOC_Os04g01530 | 0.7 | 0.0175 | up |
| LOC_Os04g29990 | 0.7 | 0.00199 | up |
| LOC_Os05g41910 | 0.7 | 0.0246 | up |
| LOC_Os06g02555 | 0.7 | 0.0262 | up |
| LOC_Os06g23910 | 0.7 | 0.0167 | up |
| LOC_Os06g47460 | 0.7 | 8.32E-06 | up |
| LOC_Os08g32090 | 0.7 | 0.0113 | up |
| LOC_Os12g35620 | 0.7 | 0.0371 | up |
| LOC_Os01g24920 | 0.69 | 0.00962 | up |
| LOC_Os01g46210 | 0.69 | 0.0187 | up |
| LOC_Os01g73720 | 0.69 | 0.0299 | up |
| LOC_Os02g01010 | 0.69 | 0.0343 | up |
| LOC_Os04g49500 | 0.69 | 0.00653 | up |
| LOC_Os05g04820 | 0.69 | 0.015 | up |
| LOC_Os05g34330 | 0.69 | 0.0372 | up |
| LOC_Os05g43090 | 0.69 | 0.0408 | up |
| LOC_Os07g01550 | 0.69 | 0.0393 | up |
| LOC_Os07g22950 | 0.69 | 0.00138 | up |
| LOC_Os07g40620 | 0.69 | 0.0451 | up |
| LOC_Os08g42630 | 0.69 | 6.04E-05 | up |
| LOC_Os09g29940 | 0.69 | 0.0252 | up |
| LOC_Os09g37540 | 0.69 | 0.00624 | up |
| LOC_Os10g30420 | 0.69 | 0.0213 | up |
| LOC_Os11g06760 | 0.69 | 0.024 | up |
| LOC_Os12g12590 | 0.69 | 0.0285 | up |
| LOC_Os12g37690 | 0.69 | 0.0125 | up |
| LOC_Os01g60480 | 0.68 | 0.0153 | up |
| LOC_Os01g71160 | 0.68 | 0.0167 | up |
| LOC_Os02g07060 | 0.68 | 0.0015 | up |
| LOC_Os02g09670 | 0.68 | 0.0289 | up |
| LOC_Os03g21220 | 0.68 | 0.0265 | up |
| LOC_Os03g52690 | 0.68 | 0.0332 | up |
| LOC_Os03g58040 | 0.68 | 0.00155 | up |
| LOC_Os04g47140 | 0.68 | 0.031 | up |
| LOC_Os04g58830 | 0.68 | 0.0414 | up |
| LOC_Os05g03750 | 0.68 | 0.0244 | up |
| LOC_Os05g39130 | 0.68 | 0.000662 | up |
| LOC_Os06g14500 | 0.68 | 0.00264 | up |
| LOC_Os06g17880 | 0.68 | 0.00596 | up |
| LOC_Os07g39310 | 0.68 | 0.00915 | up |
| LOC_Os07g44800 | 0.68 | 0.0156 | up |
| LOC_Os07g46130 | 0.68 | 0.0251 | up |
| LOC_Os10g33760 | 0.68 | 0.0328 | up |
| LOC_Os11g47350 | 0.68 | 0.0344 | up |
| LOC_Os12g43970 | 0.68 | 1.00E-12 | up |
| LOC_Os01g10710 | 0.67 | 0.0153 | up |
| LOC_Os01g33400 | 0.67 | 0.000249 | up |
| LOC_Os02g10550 | 0.67 | 0.00092 | up |
| LOC_Os03g04195 | 0.67 | 0.0317 | up |
| LOC_Os03g04230 | 0.67 | 0.047 | up |
| LOC_Os03g58270 | 0.67 | 0.0123 | up |
| LOC_Os04g12070 | 0.67 | 0.035 | up |
| LOC_Os05g30860 | 0.67 | 0.0476 | up |
| LOC_Os05g31570 | 0.67 | 0.0112 | up |
| LOC_Os05g38500 | 0.67 | 0.0369 | up |
| LOC_Os08g30690 | 0.67 | 0.0201 | up |
| LOC_Os09g33530 | 0.67 | 0.0408 | up |
| LOC_Os11g06210 | 0.67 | 4.60E-05 | up |
| LOC_Os01g06920 | 0.66 | 0.046 | up |
| LOC_Os02g19460 | 0.66 | 0.0274 | up |
| LOC_Os02g42290 | 0.66 | 0.0432 | up |
| LOC_Os02g50560 | 0.66 | 0.00222 | up |
| LOC_Os04g38042 | 0.66 | 0.0222 | up |
| LOC_Os04g52860 | 0.66 | 0.0127 | up |
| LOC_Os06g38640 | 0.66 | 0.0269 | up |
| LOC_Os06g49800 | 0.66 | 0.0272 | up |
| LOC_Os07g39590 | 0.66 | 0.0329 | up |
| LOC_Os09g39630 | 0.66 | 7.91E-08 | up |
| LOC_Os11g02470 | 0.66 | 0.00101 | up |
| LOC_Os12g02250 | 0.66 | 0.0315 | up |
| LOC_Os12g07020 | 0.66 | 0.0175 | up |
| LOC_Os02g10600 | 0.65 | 0.00935 | up |
| LOC_Os03g44310 | 0.65 | 0.0368 | up |
| LOC_Os03g48810 | 0.65 | 0.0128 | up |
| LOC_Os03g52980 | 0.65 | 0.00942 | up |
| LOC_Os04g02640 | 0.65 | 0.027 | up |
| LOC_Os04g48800 | 0.65 | 1.44E-07 | up |
| LOC_Os05g24550 | 0.65 | 0.0297 | up |
| LOC_Os06g08390 | 0.65 | 0.00155 | up |
| LOC_Os06g09560 | 0.65 | 0.0268 | up |
| LOC_Os07g10990 | 0.65 | 0.00218 | up |
| LOC_Os07g37740 | 0.65 | 0.0236 | up |
| LOC_Os12g19470 | 0.65 | 0.0112 | up |
| LOC_Os01g60340 | 0.64 | 0.0108 | up |
| LOC_Os03g01970 | 0.64 | 0.0355 | up |
| LOC_Os03g07940 | 0.64 | 0.0408 | up |
| LOC_Os04g09604 | 0.64 | 0.00347 | up |
| LOC_Os04g32270 | 0.64 | 0.04 | up |
| LOC_Os04g44700 | 0.64 | 0.0129 | up |
| LOC_Os04g52540 | 0.64 | 0.000344 | up |
| LOC_Os04g55440 | 0.64 | 0.0163 | up |
| LOC_Os05g41470 | 0.64 | 0.0451 | up |
| LOC_Os09g29380 | 0.64 | 0.0403 | up |
| LOC_Os10g30560 | 0.64 | 0.000616 | up |
| LOC_Os10g43050 | 0.64 | 0.0211 | up |
| LOC_Os11g07890 | 0.64 | 0.0343 | up |
| LOC_Os11g41420 | 0.64 | 0.00497 | up |
| LOC_Os11g45330 | 0.64 | 0.0268 | up |
| LOC_Os12g29520 | 0.64 | 0.0332 | up |
| LOC_Os12g38750 | 0.64 | 0.000618 | up |
| LOC_Os01g02370 | 0.63 | 0.0257 | up |
| LOC_Os01g52140 | 0.63 | 0.021 | up |
| LOC_Os01g61970 | 0.63 | 0.02 | up |
| LOC_Os02g18140 | 0.63 | 0.000659 | up |
| LOC_Os02g44090 | 0.63 | 0.0444 | up |
| LOC_Os03g53540 | 0.63 | 0.00923 | up |
| LOC_Os03g60520 | 0.63 | 0.0335 | up |
| LOC_Os06g08380 | 0.63 | 0.0466 | up |
| LOC_Os06g23970 | 0.63 | 0.0346 | up |
| LOC_Os06g44430 | 0.63 | 0.0239 | up |
| LOC_Os06g49930 | 0.63 | 0.00864 | up |
| LOC_Os08g34340 | 0.63 | 0.0374 | up |
| LOC_Os10g38880 | 0.63 | 0.013 | up |
| LOC_Os01g65240 | 0.62 | 0.0339 | up |
| LOC_Os01g67570 | 0.62 | 0.000132 | up |
| LOC_Os02g07300 | 0.62 | 0.0297 | up |
| LOC_Os02g45760 | 0.62 | 0.0401 | up |
| LOC_Os02g55480 | 0.62 | 0.0116 | up |
| LOC_Os03g02230 | 0.62 | 0.0325 | up |
| LOC_Os03g04020 | 0.62 | 0.0103 | up |
| LOC_Os03g55220 | 0.62 | 0.0457 | up |
| LOC_Os06g51050 | 0.62 | 0.0433 | up |
| LOC_Os07g07194 | 0.62 | 0.0466 | up |
| LOC_Os07g38240 | 0.62 | 0.00515 | up |
| LOC_Os08g06100 | 0.62 | 0.0133 | up |
| LOC_Os08g14890 | 0.62 | 0.0142 | up |
| LOC_Os08g32160 | 0.62 | 0.0398 | up |
| LOC_Os10g08590 | 0.62 | 0.0214 | up |
| LOC_Os12g02930 | 0.62 | 0.0263 | up |
| LOC_Os01g29430 | 0.61 | 0.0373 | up |
| LOC_Os01g55900 | 0.61 | 0.00575 | up |
| LOC_Os01g72230 | 0.61 | 0.0232 | up |
| LOC_Os02g06030 | 0.61 | 0.00176 | up |
| LOC_Os02g53340 | 0.61 | 0.0223 | up |
| LOC_Os04g02040 | 0.61 | 0.0269 | up |
| LOC_Os04g23600 | 0.61 | 0.0116 | up |
| LOC_Os04g49757 | 0.61 | 0.0313 | up |
| LOC_Os06g30930 | 0.61 | 0.023 | up |
| LOC_Os07g47700 | 0.61 | 0.0211 | up |
| LOC_Os07g48650 | 0.61 | 0.00779 | up |
| LOC_Os08g07860 | 0.61 | 0.000136 | up |
| LOC_Os09g37240 | 0.61 | 0.00998 | up |
| LOC_Os11g30050 | 0.61 | 0.0427 | up |
| LOC_Os12g35610 | 0.61 | 0.0202 | up |
| LOC_Os01g43410 | 0.6 | 0.0393 | up |
| LOC_Os01g49380 | 0.6 | 0.0462 | up |
| LOC_Os01g52514 | 0.6 | 0.0466 | up |
| LOC_Os01g54570 | 0.6 | 0.0228 | up |
| LOC_Os02g20934 | 0.6 | 0.0139 | up |
| LOC_Os02g56870 | 0.6 | 0.0393 | up |
| LOC_Os02g58160 | 0.6 | 0.0237 | up |
| LOC_Os03g10290 | 0.6 | 5.60E-10 | up |
| LOC_Os03g19120 | 0.6 | 0.0372 | up |
| LOC_Os03g20320 | 0.6 | 0.00824 | up |
| LOC_Os03g51000 | 0.6 | 0.0476 | up |
| LOC_Os04g45080 | 0.6 | 0.0071 | up |
| LOC_Os04g55230 | 0.6 | 0.0353 | up |
| LOC_Os05g02240 | 0.6 | 0.00245 | up |
| LOC_Os05g41620 | 0.6 | 0.00109 | up |
| LOC_Os06g38970 | 0.6 | 0.0405 | up |
| LOC_Os07g04990 | 0.6 | 0.0419 | up |
| LOC_Os08g05750 | 0.6 | 0.00109 | up |
| LOC_Os09g20684 | 0.6 | 0.0368 | up |
| LOC_Os10g38070 | 0.6 | 0.0243 | up |
| LOC_Os11g10230 | 0.6 | 0.00783 | up |
| LOC_Os01g54600 | 0.59 | 0.013 | up |
| LOC_Os01g61860 | 0.59 | 0.0257 | up |
| LOC_Os01g64262 | 0.59 | 0.0223 | up |
| LOC_Os01g67054 | 0.59 | 0.0407 | up |
| LOC_Os02g05940 | 0.59 | 0.00586 | up |
| LOC_Os02g45010 | 0.59 | 0.0287 | up |
| LOC_Os02g48090 | 0.59 | 0.0478 | up |
| LOC_Os02g53130 | 0.59 | 0.0276 | up |
| LOC_Os03g12660 | 0.59 | 0.0348 | up |
| LOC_Os03g50470 | 0.59 | 0.0182 | up |
| LOC_Os03g54040 | 0.59 | 0.0362 | up |
| LOC_Os04g13300 | 0.59 | 0.0271 | up |
| LOC_Os04g51900 | 0.59 | 0.0384 | up |
| LOC_Os04g55660 | 0.59 | 0.0201 | up |
| LOC_Os05g24530 | 0.59 | 0.000222 | up |
| LOC_Os05g48120 | 0.59 | 7.04E-11 | up |
| LOC_Os09g39620 | 0.59 | 0.0177 | up |
| LOC_Os01g04250 | 0.58 | 0.0116 | up |
| LOC_Os01g37870 | 0.58 | 0.00169 | up |
| LOC_Os02g15350 | 0.58 | 0.0397 | up |
| LOC_Os02g40860 | 0.58 | 0.0494 | up |
| LOC_Os02g50790 | 0.58 | 0.00593 | up |
| LOC_Os03g55240 | 0.58 | 0.00915 | up |
| LOC_Os04g40720 | 0.58 | 6.99E-07 | up |
| LOC_Os04g42470 | 0.58 | 0.014 | up |
| LOC_Os04g52830 | 0.58 | 0.0234 | up |
| LOC_Os06g09990 | 0.58 | 0.00931 | up |
| LOC_Os06g20960 | 0.58 | 0.0447 | up |
| LOC_Os06g46340 | 0.58 | 0.00633 | up |
| LOC_Os08g44560 | 0.58 | 0.00535 | up |
| LOC_Os09g37020 | 0.58 | 0.0379 | up |
| LOC_Os10g11310 | 0.58 | 0.0395 | up |
| LOC_Os10g14230 | 0.58 | 0.0399 | up |
| LOC_Os10g26690 | 0.58 | 0.011 | up |
| LOC_Os12g41124 | 0.58 | 0.000103 | up |
| LOC_Os01g15520 | -0.79 | 0.0226 | down |
| LOC_Os01g28260 | -2.2 | 0.000437 | down |
| LOC_Os01g28850 | -0.8 | 0.0307 | down |
| LOC_Os02g06630 | -1.73 | 5.68E-06 | down |
| LOC_Os02g17200 | -0.79 | 0.00019 | down |
| LOC_Os02g34370 | -0.82 | 0.0244 | down |
| LOC_Os03g05080 | -0.67 | 0.0392 | down |
| LOC_Os03g10530 | -0.6 | 0.0136 | down |
| LOC_Os03g18779 | -0.67 | 0.0302 | down |
| LOC_Os03g26250 | -1.1 | 3.16E-06 | down |
| LOC_Os03g41339 | -0.78 | 0.00726 | down |
| LOC_Os03g44940 | -0.85 | 0.0402 | down |
| LOC_Os03g57630 | -0.63 | 0.0032 | down |
| LOC_Os04g01920 | -0.59 | 0.0151 | down |
| LOC_Os04g18400 | -7.38 | 2.68E-51 | down |
| LOC_Os04g31470 | -0.64 | 0.00659 | down |
| LOC_Os04g42120 | -1.55 | 1.41E-20 | down |
| LOC_Os04g43840 | -0.81 | 0.0474 | down |
| LOC_Os04g49600 | -0.77 | 6.07E-05 | down |
| LOC_Os05g23650 | -0.97 | 1.24E-06 | down |
| LOC_Os05g30070 | -3.29 | 1.29E-18 | down |
| LOC_Os05g34290 | -0.8 | 0.0118 | down |
| LOC_Os06g06990 | -0.87 | 5.38E-06 | down |
| LOC_Os06g21910 | -0.73 | 0.0119 | down |
| LOC_Os06g44790 | -0.58 | 0.00891 | down |
| LOC_Os06g46030 | -0.9 | 0.0217 | down |
| LOC_Os07g01904 | -1.56 | 9.26E-08 | down |
| LOC_Os07g04060 | -1 | 0.0172 | down |
| LOC_Os07g11020 | -0.62 | 0.0471 | down |
| LOC_Os07g32710 | -0.64 | 0.00682 | down |
| LOC_Os07g41080 | -0.62 | 0.0235 | down |
| LOC_Os08g10870 | -1.49 | 0.000468 | down |
| LOC_Os08g14850 | -0.69 | 0.0196 | down |
| LOC_Os08g22660 | -4.64 | 4.79E-13 | down |
| LOC_Os08g27824 | -1.15 | 8.11E-05 | down |
| LOC_Os08g35220 | -0.75 | 0.0098 | down |
| LOC_Os08g42630 | -1.39 | 6.12E-07 | down |
| LOC_Os09g08730 | -0.91 | 0.0372 | down |
| LOC_Os09g09370 | -0.64 | 0.00944 | down |
| LOC_Os09g34950 | -0.65 | 1.55E-07 | down |
| LOC_Os10g02450 | -1.46 | 0.0051 | down |
| LOC_Os10g04770 | -1.3 | 0.0366 | down |
| LOC_Os10g06770 | -0.91 | 0.0335 | down |
| LOC_Os10g07160 | -1.02 | 8.43E-05 | down |
| LOC_Os10g22150 | -1.51 | 1.12E-34 | down |
| LOC_Os10g41838 | -0.77 | 0.00118 | down |
| LOC_Os11g08240 | -0.97 | 0.0244 | down |
| LOC_Os11g10460 | -0.68 | 0.0497 | down |
| LOC_Os11g10570 | -1.03 | 0.00796 | down |
| LOC_Os11g20330 | -0.77 | 0.0471 | down |
| LOC_Os11g35440 | -0.74 | 0.000109 | down |
| LOC_Os11g45320 | -0.81 | 0.0485 | down |
| LOC_Os11g47760 | -0.74 | 0.0403 | down |
| LOC_Os12g03840 | -1.78 | 0.0162 | down |
| LOC_Os12g09660 | -1.17 | 0.0248 | down |
| LOC_Os12g24050 | -1.2 | 0.00763 | down |
| LOC_Os12g29360 | -0.93 | 0.00632 | down |
| LOC_Os12g34470 | -0.75 | 0.00185 | down |
| LOC_Os12g38360 | -1.27 | 0.000342 | down |
| LOC_Os12g38410 | -0.68 | 0.0195 | down |
| LOC_Os12g38420 | -1.5 | 5.80E-06 | down |

**Table S5.** GO pathways of H4K5ac and H4K8ac upregulated genes in *ossrt2* mutant vs WT.

| **ID** | **Description** | ***P* value** |
| --- | --- | --- |
| GO:0048573 | photoperiodism, flowering | 0.002521046 |
| GO:0009648 | photoperiodism | 0.0029067 |
| GO:0090502 | RNA phosphodiester bond hydrolysis, endonucleolytic | 0.003008144 |
| GO:0046685 | response to arsenic-containing substance | 0.004629014 |
| GO:0044403 | biological process involved in symbiotic interaction | 0.004936605 |
| GO:0090501 | RNA phosphodiester bond hydrolysis | 0.006788809 |
| GO:0046149 | pigment catabolic process | 0.006896666 |
| GO:0007140 | male meiotic nuclear division | 0.008766793 |
| GO:0010228 | vegetative to reproductive phase transition of meristem | 0.00943113 |
| GO:0009625 | response to insect | 0.00998567 |
| GO:0006826 | iron ion transport | 0.010837677 |
| GO:0044003 | modulation by symbiont of host process | 0.011720719 |
| GO:0030422 | production of siRNA involved in post-transcriptional gene silencing by RNA | 0.012634369 |
| GO:0140013 | meiotic nuclear division | 0.01431485 |
| GO:0035821 | modulation of process of another organism | 0.015554762 |
| GO:0009739 | response to gibberellin | 0.01636288 |
| GO:0071359 | cellular response to dsRNA | 0.020995585 |
| GO:0043331 | response to dsRNA | 0.022166166 |
| GO:0051701 | biological process involved in interaction with host | 0.024586624 |
| GO:1903046 | meiotic cell cycle process | 0.026477094 |
| GO:0048574 | long-day photoperiodism, flowering | 0.02906891 |
| GO:0070918 | primary sncRNA processing | 0.029734099 |
| GO:0009626 | plant-type hypersensitive response | 0.031765953 |
| GO:0034050 | programmed cell death induced by symbiont | 0.031765953 |
| GO:0048571 | long-day photoperiodism | 0.031765953 |
| GO:0002084 | protein depalmitoylation | 0.032389562 |
| GO:0042159 | lipoprotein catabolic process | 0.032389562 |
| GO:0046275 | flavonoid catabolic process | 0.032389562 |
| GO:0046284 | anthocyanin-containing compound catabolic process | 0.032389562 |
| GO:0071588 | hydrogen peroxide mediated signaling pathway | 0.032389562 |
| GO:0098734 | macromolecule depalmitoylation | 0.032389562 |
| GO:1900000 | regulation of anthocyanin catabolic process | 0.032389562 |
| GO:0051321 | meiotic cell cycle | 0.035523124 |
| GO:1901678 | iron coordination entity transport | 0.035571423 |
| GO:0051702 | biological process involved in interaction with symbiont | 0.03598891 |
| GO:0006222 | UMP biosynthetic process | 0.038743034 |
| GO:0009173 | pyrimidine ribonucleoside monophosphate metabolic process | 0.038743034 |
| GO:0009174 | pyrimidine ribonucleoside monophosphate biosynthetic process | 0.038743034 |
| GO:0046049 | UMP metabolic process | 0.038743034 |
| GO:0046283 | anthocyanin-containing compound metabolic process | 0.038918357 |
| GO:0046777 | protein autophosphorylation | 0.039708042 |
| GO:1901657 | glycosyl compound metabolic process | 0.04057403 |
| GO:0000712 | resolution of meiotic recombination intermediates | 0.041904427 |
| GO:0006825 | copper ion transport | 0.041904427 |
| GO:0009129 | pyrimidine nucleoside monophosphate metabolic process | 0.041904427 |
| GO:0009130 | pyrimidine nucleoside monophosphate biosynthetic process | 0.041904427 |
| GO:0010623 | programmed cell death involved in cell development | 0.041904427 |
| GO:0045493 | xylan catabolic process | 0.041904427 |
| GO:1901527 | abscisic acid-activated signaling pathway involved in stomatal movement | 0.041904427 |
| GO:0009624 | response to nematode | 0.043476848 |
| GO:0043094 | cellular metabolic compound salvage | 0.043476848 |
| GO:0010102 | lateral root morphogenesis | 0.044255225 |
| GO:0002478 | antigen processing and presentation of exogenous peptide antigen | 0.045055634 |
| GO:0006878 | cellular copper ion homeostasis | 0.045055634 |
| GO:0010600 | regulation of auxin biosynthetic process | 0.045055634 |
| GO:0019048 | modulation by virus of host process | 0.045055634 |
| GO:0019884 | antigen processing and presentation of exogenous antigen | 0.045055634 |
| GO:0019886 | antigen processing and presentation of exogenous peptide antigen via MHC class II | 0.045055634 |
| GO:0010101 | post-embryonic root morphogenesis | 0.045827576 |
| GO:0035194 | post-transcriptional gene silencing by RNA | 0.045827576 |
| GO:0042440 | pigment metabolic process | 0.047845714 |
| GO:0007112 | male meiosis cytokinesis | 0.048196687 |

**Table S6.** H4K5ac, H4K8ac and transcriptionally upregulated genes in *ossrt2* mutant vs WT.

| **Gene ID** | **H4K5ac Log_2_FC** | **H4K8ac Log_2_FC** | **Transcriptional Log_2_FC** |
| --- | --- | --- | --- |
| LOC_Os02g12470 | 7.57 | 7.89 | 5.861076535 |
| LOC_Os09g10650 | 4.41 | 7.68 | 5.091571374 |
| LOC_Os09g09540 | 1.4 | 1.13 | 1.9678598 |
| LOC_Os09g38104 | 1.47 | 1.69 | 4.594337109 |
| LOC_Os03g43100 | 1.37 | 1.69 | 9.425878178 |
| LOC_Os11g35300 | 5.25 | 4.62 | 9.548640753 |
| LOC_Os12g02140 | 5.12 | 4.03 | 5.894894355 |
| LOC_Os05g34490 | 1.93 | 2.64 | 6.041136254 |
| LOC_Os02g52730 | 5.7 | 3.71 | 4.192826173 |
| LOC_Os06g20740 | 4.03 | 2.96 | 5.884940792 |
| LOC_Os04g52790 | 2.27 | 2.56 | 6.368729591 |
| LOC_Os04g17660 | 0.77 | 0.79 | 2.555605143 |
| LOC_Os03g18779 | 1.48 | 2.92 | 11.72619388 |
| LOC_Os05g23050 | 2.64 | 3.02 | 5.564555674 |
| LOC_Os03g10290 | 0.71 | 0.6 | 2.901896732 |
| LOC_Os09g10660 | 4.26 | 4.91 | 7.340439005 |
| LOC_Os07g01904 | 0.86 | 0.78 | 2.343760806 |
| LOC_Os11g10250 | 0.58 | 0.87 | 6.643646107 |
| LOC_Os12g39280 | 4.44 | 4.27 | 6.797820911 |
| LOC_Os03g02230 | 1.27 | 0.62 | 4.447876246 |
| LOC_Os05g37810 | 6.92 | 7.56 | 6.620024505 |
| LOC_Os03g27400 | 7.97 | 6.46 | 6.604866487 |
| LOC_Os06g40240 | 1.9 | 1.93 | 4.705503685 |
| LOC_Os12g12690 | 5.11 | 4.45 | 6.027667524 |
| LOC_Os05g08940 | 5.61 | 8.38 | 3.594244439 |
| LOC_Os10g39190 | 0.98 | 1.09 | 2.810764657 |
| LOC_Os12g07180 | 3.55 | 3.4 | 9.682169278 |
| LOC_Os10g04520 | 0.84 | 1.72 | 1.407409841 |
| LOC_Os04g34500 | 3.56 | 2.16 | 10.29822023 |
| LOC_Os07g42560 | 0.74 | 0.98 | 3.412443382 |
| LOC_Os02g21320 | 1.39 | 1.95 | 5.031467847 |
| LOC_Os08g09990 | 1.59 | 1.46 | 8.790587131 |
| LOC_Os05g07170 | 1.96 | 1.56 | 6.107053356 |
| LOC_Os05g45170 | 3.48 | 3.49 | 7.369605249 |
| LOC_Os07g10850 | 2.44 | 3.04 | 4.295173337 |
| LOC_Os07g05450 | 2.46 | 4.73 | 4.366132526 |
| LOC_Os01g62540 | 3.02 | 3.92 | 4.075142599 |
| LOC_Os08g11150 | 0.75 | 0.84 | 4.31900983 |
| LOC_Os01g41960 | 0.74 | 0.99 | 7.911613786 |
| LOC_Os04g14690 | 6.66 | 8.55 | 2.620064703 |
| LOC_Os10g24004 | 7.17 | 7.53 | 8.333166882 |
| LOC_Os05g50390 | 1.69 | 0.93 | 10.50865971 |
| LOC_Os06g49350 | 2.45 | 3.03 | 3.792277549 |
| LOC_Os03g55460 | 1.48 | 1.64 | 1.868633074 |
| LOC_Os03g08350 | 1.45 | 1.79 | 2.786486529 |
| LOC_Os06g14870 | 2.19 | 3.2 | 3.7833166 |
| LOC_Os03g26250 | 3.01 | 2.91 | 3.895671144 |
| LOC_Os06g21369 | 2.02 | 2.1 | 7.687545647 |
| LOC_Os07g13770 | 0.62 | 1.02 | 1.392157187 |
| LOC_Os01g42420 | 3.2 | 3.63 | 4.56250968 |
| LOC_Os11g02220 | 1.44 | 2.38 | 4.396942512 |
| LOC_Os03g22130 | 0.98 | #N/A | 4.234997066 |
| LOC_Os08g07730 | 0.61 | #N/A | 1.486911868 |
| LOC_Os02g55900 | 0.6 | #N/A | 3.455768583 |
| LOC_Os01g74430 | 0.63 | #N/A | 4.437729791 |
| LOC_Os06g07420 | 1.27 | #N/A | 5.955335379 |
| LOC_Os03g02470 | 3.61 | #N/A | 12.62504617 |
| LOC_Os08g38800 | 0.59 | #N/A | 3.187995712 |
| LOC_Os12g05290 | 4.19 | #N/A | 3.860567076 |
| LOC_Os03g14820 | 0.7 | #N/A | 5.749546197 |
| LOC_Os01g15520 | 2.58 | #N/A | 1.711829713 |
| LOC_Os04g27340 | 0.9 | #N/A | 2.160808602 |
| LOC_Os12g33194 | 2.98 | #N/A | 1.324241018 |
| LOC_Os09g39630 | #N/A | 0.66 | 2.587856228 |
| LOC_Os02g15350 | #N/A | 0.58 | 8.157331111 |
| LOC_Os03g55220 | #N/A | 0.62 | 5.182551697 |
| LOC_Os07g41400 | #N/A | 4.93 | 3.917238561 |
| LOC_Os12g35000 | #N/A | 0.71 | 6.822673652 |
| LOC_Os02g37770 | #N/A | 1.41 | 1.06739879 |
| LOC_Os02g55330 | #N/A | 0.83 | 1.031187688 |
| LOC_Os11g10710 | #N/A | 0.97 | 4.108372534 |
| LOC_Os11g36810 | #N/A | 0.76 | 1.201682684 |
| LOC_Os08g31720 | #N/A | 1.61 | 2.786708695 |
| LOC_Os11g04030 | #N/A | 1.91 | 1.511228969 |
| LOC_Os08g28890 | #N/A | 0.93 | 1.510395376 |
| LOC_Os02g06630 | #N/A | 1.42 | 1.885572622 |
| LOC_Os09g20684 | #N/A | 0.6 | 1.192292628 |
| LOC_Os05g23650 | #N/A | 3.05 | 4.756592987 |
| LOC_Os04g09604 | #N/A | 0.64 | 2.444097029 |
| LOC_Os06g09980 | #N/A | 0.71 | 2.25384311 |
| LOC_Os05g23950 | #N/A | 0.75 | 1.057229464 |
| LOC_Os02g31845 | #N/A | 1.94 | 1.377061174 |
| LOC_Os12g24050 | #N/A | 0.76 | 7.74453305 |
| LOC_Os11g02540 | #N/A | 0.89 | 3.992669465 |

**Table S7.** GO pathways of H4K5ac, H4K8ac and transcriptionally upregulated genes in *ossrt2* mutant vs WT.

| **ID** | **Description** | ***P* value** |
| --- | --- | --- |
| GO:0034754 | cellular hormone metabolic process | 0.00070375 |
| GO:0009812 | flavonoid metabolic process | 0.00412462 |
| GO:0046275 | flavonoid catabolic process | 0.00535384 |
| GO:0046284 | anthocyanin-containing compound catabolic process | 0.00535384 |
| GO:1900000 | regulation of anthocyanin catabolic process | 0.00535384 |
| GO:0098754 | detoxification | 0.00562178 |
| GO:1901527 | abscisic acid-activated signaling pathway involved in stomatal movement | 0.0069551 |
| GO:0042440 | pigment metabolic process | 0.007221 |
| GO:0010067 | procambium histogenesis | 0.0085541 |
| GO:0030186 | melatonin metabolic process | 0.0085541 |
| GO:0030187 | melatonin biosynthetic process | 0.0085541 |
| GO:0009269 | response to desiccation | 0.00961884 |
| GO:0016139 | glycoside catabolic process | 0.00961884 |
| GO:0010065 | primary meristem tissue development | 0.01174534 |
| GO:0051552 | flavone metabolic process | 0.01174534 |
| GO:0051553 | flavone biosynthetic process | 0.01174534 |
| GO:0051554 | flavonol metabolic process | 0.01174534 |
| GO:0051555 | flavonol biosynthetic process | 0.01174534 |
| GO:0042445 | hormone metabolic process | 0.01385634 |
| GO:0016137 | glycoside metabolic process | 0.01386783 |
| GO:0042147 | retrograde transport, endosome to Golgi | 0.01651534 |
| GO:0046685 | response to arsenic-containing substance | 0.01651534 |
| GO:0009636 | response to toxic substance | 0.01663029 |
| GO:0046149 | pigment catabolic process | 0.02021141 |
| GO:0046854 | phosphatidylinositol phosphate biosynthetic process | 0.02021141 |
| GO:0046834 | lipid phosphorylation | 0.02073843 |
| GO:0071456 | cellular response to hypoxia | 0.02126519 |
| GO:0009690 | cytokinin metabolic process | 0.02179171 |
| GO:0048508 | embryonic meristem development | 0.02179171 |
| GO:0036294 | cellular response to decreased oxygen levels | 0.02284401 |
| GO:0071453 | cellular response to oxygen levels | 0.02284401 |
| GO:0010167 | response to nitrate | 0.02336979 |
| GO:0031537 | regulation of anthocyanin metabolic process | 0.0244206 |
| GO:1901658 | glycosyl compound catabolic process | 0.02494563 |
| GO:0010817 | regulation of hormone levels | 0.02585713 |
| GO:0016482 | cytosolic transport | 0.02861396 |
| GO:0042538 | hyperosmotic salinity response | 0.03122679 |
| GO:0042631 | cellular response to water deprivation | 0.03122679 |
| GO:0071462 | cellular response to water stimulus | 0.03122679 |
| GO:0009809 | lignin biosynthetic process | 0.03279153 |
| GO:0006661 | phosphatidylinositol biosynthetic process | 0.03435407 |
| GO:1901136 | carbohydrate derivative catabolic process | 0.03643401 |
| GO:0009808 | lignin metabolic process | 0.03799139 |
| GO:0042435 | indole-containing compound biosynthetic process | 0.04006448 |
| GO:0002239 | response to oomycetes | 0.04058214 |
| GO:0006972 | hyperosmotic response | 0.04058214 |
| GO:0002218 | activation of innate immune response | 0.04213365 |
| GO:0009626 | plant-type hypersensitive response | 0.04471463 |
| GO:0034050 | programmed cell death induced by symbiont | 0.04471463 |
| GO:0016197 | endosomal transport | 0.04523009 |
| GO:0042430 | indole-containing compound metabolic process | 0.04574532 |
| GO:0046488 | phosphatidylinositol metabolic process | 0.04677503 |
| GO:0051702 | biological process involved in interaction with symbiont | 0.04780377 |
| GO:0046283 | anthocyanin-containing compound metabolic process | 0.04985834 |
| GO:0002253 | activation of immune response | 0.049912 |
